# Supplementary material for: The dynamic shape changes of the tongue base during respiration, chewing and swallowing
Source: PLoS One. 2025 Apr 14;20(4):e0315885. doi: 10.1371/journal.pone.0315885 (PMC11996074; doi:10.1371/journal.pone.0315885)
Supplement: S1 File — (PDF) [file pone.0315885.s001.pdf]

Sono respiration_Raw data.sav

	pig_code	ID_gender	Pair_ID	Cycle_total	P_or_V	onset_ref
1	5518	1	1	.000	0	0
2	5518	1	1	.000	0	0
3	5518	1	3	.000	0	0
4	5518	1	3	.000	0	0
5	5518	1	5	.000	0	0
6	5518	1	6	.000	0	0
7	5518	1	7	.000	0	0
8	5518	1	8	.000	0	0
9	5518	1	9	.000	0	0
10	5518	1	9	.000	0	0
11	5518	1	11	.000	0	0
12	5518	1	11	.000	0	0
13	5794	2	1	.000	0	0
14	5794	2	1	.000	0	0
15	5794	2	3	.000	0	0
16	5794	2	3	.000	0	0
17	5794	2	5	.000	0	0
18	5794	2	6	.000	0	0
19	5794	2	7	.000	0	0
20	5794	2	8	.000	0	0
21	5794	2	9	.000	0	0
22	5794	2	9	.000	0	0
23	5794	2	11	.000	0	0
24	5794	2	11	.000	0	0
25	5564	2	1	1.000	1	0
26	5564	2	1	1.000	1	0
27	5564	2	3	1.000	1	0
28	5564	2	3	1.000	1	0
29	5564	2	5	1.000	1	0
30	5564	2	6	1.000	2	0
31	5564	2	7	1.000	1	0
32	5564	2	8	1.000	2	0
33	5564	2	9	1.000	0	0
34	5564	2	9	1.000	1	0
35	5564	2	11	1.000	1	0

Sono respiration_Raw data.sav

	resp_duration	insp_duration	exp_duration	base_value	Peak_or_Valley	Amplitude
1	0	.000	.000	.000	.000	.000
2	0	.000	.000	.000	.000	.000
3	0	.000	.000	.000	.000	.000
4	0	.000	.000	.000	.000	.000
5	0	.000	.000	.000	.000	.000
6	0	.000	.000	.000	.000	.000
7	0	.000	.000	.000	.000	.000
8	0	.000	.000	.000	.000	.000
9	0	.000	.000	.000	.000	.000
10	0	.000	.000	.000	.000	.000
11	0	.000	.000	.000	.000	.000
12	0	.000	.000	.000	.000	.000
13	0	.000	.000	.000	.000	.000
14	0	.000	.000	.000	.000	.000
15	0	.000	.000	.000	.000	.000
16	0	.000	.000	.000	.000	.000
17	0	.000	.000	.000	.000	.000
18	0	.000	.000	.000	.000	.000
19	0	.000	.000	.000	.000	.000
20	0	.000	.000	.000	.000	.000
21	0	.000	.000	.000	.000	.000
22	0	.000	.000	.000	.000	.000
23	0	.000	.000	.000	.000	.000
24	0	.000	.000	.000	.000	.000
25	3	.629	2.691	-9.340	9.430	.090
26	3	.764	2.578	-17.070	17.500	.430
27	3	.659	2.668	-17.890	18.090	.200
28	3	.697	2.615	-10.460	10.950	.490
29	3	.510	2.869	-20.750	20.930	.180
30	3	.517	2.703	-32.660	-32.790	.130
31	3	.480	2.705	-25.710	25.870	.160
32	3	.442	2.892	27.860	-27.690	.170
33	0	.000	.000	.000	.000	.000
34	3	.644	2.653	-5.960	6.100	.140
35	3	.652	2.630	-6.830	6.930	.100

Sono respiration_Raw data.sav

	onset_percentage	duration_percentage	amplitude_percentage	P_V	onset
1	.0000	.000	.000	.000	.000
2	.0000	.000	.000	.000	.000
3	.0000	.000	.000	.000	.000
4	.0000	.000	.000	.000	.000
5	.0000	.000	.000	.000	.000
6	.0000	.000	.000	.000	.000
7	.0000	.000	.000	.000	.000
8	.0000	.000	.000	.000	.000
9	.0000	.000	.000	.000	.000
10	.0000	.000	.000	.000	.000
11	.0000	.000	.000	.000	.000
12	.0000	.000	.000	.000	.000
13	.0000	.000	.000	.000	.000
14	.0000	.000	.000	.000	.000
15	.0000	.000	.000	.000	.000
16	.0000	.000	.000	.000	.000
17	.0000	.000	.000	.000	.000
18	.0000	.000	.000	.000	.000
19	.0000	.000	.000	.000	.000
20	.0000	.000	.000	.000	.000
21	.0000	.000	.000	.000	.000
22	.0000	.000	.000	.000	.000
23	.0000	.000	.000	.000	.000
24	.0000	.000	.000	.000	.000
25	-9.3223	98.254	.954	.000	8.265
26	-13.7615	98.905	2.457	.000	8.115
27	-12.4297	98.461	1.106	.000	8.160
28	-12.6665	98.017	4.475	.000	8.152
29	.0000	100.000	.860	.000	8.580
30	-1.3613	95.294	-.396	.000	8.534
31	.0000	94.259	.618	.000	8.580
32	-13.5543	98.668	-.614	.000	8.122
33	.0000	.000	.000	.000	.000
34	-13.3175	97.573	2.295	.000	8.130
35	-8.8784	97.129	1.443	.000	8.280

	pig_code	ID_gender	Pair_ID	Cycle_total	P_or_V	onset_ref
36	5564	2	11	1.000	1	0
37	5564	2	1	2.000	1	0
38	5564	2	1	2.000	1	0
39	5564	2	3	2.000	1	0
40	5564	2	3	2.000	1	0
41	5564	2	5	2.000	1	0
42	5564	2	6	2.000	2	0
43	5564	2	7	2.000	1	0
44	5564	2	8	2.000	2	-1
45	5564	2	9	2.000	0	0
46	5564	2	9	2.000	1	0
47	5564	2	11	2.000	1	0
48	5564	2	11	2.000	1	-1
49	5564	2	1	3.000	1	0
50	5564	2	1	3.000	1	0
51	5564	2	3	3.000	1	0
52	5564	2	3	3.000	1	0
53	5564	2	5	3.000	1	0
54	5564	2	6	3.000	2	0
55	5564	2	7	3.000	1	0
56	5564	2	8	3.000	2	0
57	5564	2	9	3.000	0	0
58	5564	2	9	3.000	1	0
59	5564	2	11	3.000	1	0
60	5564	2	11	3.000	1	0
61	?	?	?	?	?	?
62	?	?	?	?	?	?
63	?	?	?	?	?	?
64	?	?	?	?	?	?
65	?	?	?	?	?	?
66	?	?	?	?	?	?
67	?	?	?	?	?	?
68	?	?	?	?	?	?
69	?	?	?	?	?	?
70	?	?	?	?	?	?

	resp_duration	insp_duration	exp_duration	base_value	Peak_or_Valley	Amplitude
36	3	.697	2.623	-9.760	9.840	.080
37	3	.570	2.562	-9.340	9.410	.070
38	3	.667	2.667	-17.070	17.500	.430
39	3	.651	2.616	-17.880	18.060	.180
40	3	.674	2.631	-10.460	10.940	.480
41	3	.352	2.810	-20.740	20.910	.170
42	3	.487	2.885	-32.680	-32.800	.120
43	3	.577	2.780	-25.720	25.860	.140
44	3	.495	2.839	27.870	-27.710	.160
45	0	.000	.000	.000	.000	.000
46	3	.674	2.675	-5.960	6.090	.130
47	3	.810	2.600	-6.800	6.910	.110
48	3	.720	2.660	-9.760	9.840	.080
49	3	.682	2.480	-9.350	9.420	.070
50	3	.577	2.645	-17.110	17.470	.360
51	3	.802	2.555	-17.910	18.050	.140
52	3	.599	2.608	-10.520	10.900	.380
53	3	.487	2.795	-20.750	20.920	.170
54	3	.457	2.740	-32.700	-32.780	.080
55	3	.502	2.758	-25.710	25.860	.150
56	3	.435	2.802	27.870	-27.720	.150
57	0	.000	.000	.000	.000	.000
58	3	.614	2.638	-5.970	6.080	.110
59	3	.652	2.518	-6.820	6.900	.080
60	3	.599	2.653	-9.770	9.820	.050
61	3	.682	2.518	-9.340	?	?
62	3	.569	2.653	-17.070	?	?
63	3	.697	2.457	-17.900	?	?
64	3	.637	2.570	-10.490	?	?
65	3	.472	2.810	-20.760	?	?
66	3	.480	2.584	-32.690	?	?
67	3	.510	2.802	-25.710	?	?
68	3	.352	2.863	27.840	?	?
69	0	.000	.000	.000	?	?
70	3	.644	2.586	-5.970	?	?

	onset_percentage	duration_percentage	amplitude_percentage	P_V	onset
36	-13.7615	98.254	.813	.000	8.115
37	-12.7767	99.051	.744	.000	11.547
38	-14.8956	105.440	2.457	.000	11.480
39	-14.4529	103.321	.997	.000	11.494
40	-14.4529	104.522	4.388	.000	11.494
41	.0000	100.000	.813	.000	11.951
42	-5.1866	106.641	-.366	.000	11.787
43	-6.6097	106.167	.541	.000	11.742
44	-16.3188	105.440	-.577	.000	11.435
45	.0000	.000	.000	.000	.000
46	-15.1486	105.914	2.135	.000	11.472
47	-15.1486	107.843	1.592	.000	11.472
48	-17.0462	106.894	.813	.000	11.412
49	-9.5673	96.344	.743	.000	14.800
50	-8.6533	98.172	2.061	.000	14.830
51	-14.1377	102.285	.776	.000	14.650
52	-9.1408	97.715	3.486	.000	14.814
53	.0000	100.000	.813	.000	15.114
54	-.2438	97.410	-.244	.000	15.106
55	-.2438	99.330	.580	.000	15.106
56	-10.9689	98.629	-.541	.000	14.754
57	.0000	.000	.000	.000	.000
58	-9.1408	99.086	1.809	.000	14.814
59	-4.7837	96.587	1.159	.000	14.957
60	-9.5978	99.086	.509	.000	14.799
61	-13.0104	97.502	.954	.000	17.961
62	-10.7252	98.172	2.290	.000	18.036
63	-10.2681	96.100	.831	.000	18.051
64	-10.7252	97.715	3.585	.000	18.036
65	.0000	100.000	.812	.000	18.388
66	-2.0719	93.358	-.517	.000	18.320
67	-2.0414	100.914	.580	.000	18.321
68	-11.6392	97.959	-.505	.000	18.006
69	.0000	.000	.000	.000	.000
70	-10.2681	98.416	1.809	.000	18.051

	pig_code	ID_gender	Pair_ID	Cycle_total	P_or_V	onset_ref
71	5564	2	11	4.000	1	0
72	5564	2	11	4.000	1	0
73	5564	2	1	5.000	1	0
74	5564	2	1	5.000	1	0
75	5564	2	3	5.000	1	0
76	5564	2	3	5.000	1	0
77	5564	2	5	5.000	1	0
78	5564	2	6	5.000	2	0
79	5564	2	7	5.000	1	0
80	5564	2	8	5.000	2	0
81	5564	2	9	5.000	0	0
82	5564	2	9	5.000	1	0
83	5564	2	11	5.000	1	0
84	5564	2	11	5.000	1	0
85	5564	2	1	6.000	1	0
86	5564	2	1	6.000	1	0
87	5564	2	3	6.000	1	-1
88	5564	2	3	6.000	1	-1
89	5564	2	5	6.000	1	0
90	5564	2	6	6.000	2	0
91	5564	2	7	6.000	1	0
92	5564	2	8	6.000	2	-1
93	5564	2	9	6.000	0	0
94	5564	2	9	6.000	1	-1
95	5564	2	11	6.000	1	0
96	5564	2	11	6.000	1	0
97	5564	2	1	7.000	1	0
98	5564	2	1	7.000	1	0
99	5564	2	3	7.000	1	0
100	5564	2	3	7.000	1	0
101	?	?	?	?	?	?
102	?	?	?	?	?	?
103	?	?	?	?	?	?
104	?	?	?	?	?	?
105	?	?	?	?	?	?

Sono respiration_Raw data.sav

	resp_duration	insp_duration	exp_duration	base_value	Peak_or_Valley	Amplitude
71	3	.689	2.548	-6.800	6.890	.090
72	3	.532	2.668	-9.760	9.830	.070
73	3	.689	2.616	-9.340	9.420	.080
74	3	.614	2.646	-17.090	17.490	.400
75	3	.674	2.518	-17.900	18.060	.160
76	3	.614	2.503	-10.510	10.940	.430
77	3	.390	2.900	-20.730	20.920	.190
78	4	.847	2.734	-32.670	-32.790	.120
79	3	.397	2.765	-25.710	25.850	.140
80	3	.427	2.645	27.840	-27.690	.150
81	0	.000	.000	.000	.000	.000
82	3	.629	2.645	-5.970	6.090	.120
83	3	.300	2.690	-6.820	6.910	.090
84	3	.525	2.780	-9.790	9.830	.040
85	3	.659	2.608	-9.350	9.410	.060
86	3	.584	2.668	-17.080	17.460	.380
87	3	.929	2.465	-17.910	18.030	.120
88	3	.689	2.690	-10.500	10.890	.390
89	3	.322	2.810	-20.760	20.910	.150
90	3	.555	2.480	-32.670	-32.760	.090
91	3	.510	2.850	-25.720	25.860	.140
92	3	.457	2.914	27.850	-27.700	.150
93	0	.000	.000	.000	.000	.000
94	3	.584	2.750	-5.970	6.080	.110
95	3	.659	2.548	-6.820	6.900	.080
96	3	.540	2.690	-9.760	9.830	.070
97	3	.599	2.601	-9.350	9.420	.070
98	3	.659	2.601	-17.070	17.440	.370
99	3	.644	2.586	-17.910	18.030	.120
100	3	.704	2.556	-10.510	10.890	.380
101	3	.420	2.997	-20.740	?	?
102	3	.390	2.630	-32.670	?	?
103	3	.465	2.884	-25.700	?	?
104	3	.427	2.720	27.840	?	?
105	0	.000	.000	.000	?	?

	onset_percentage	duration_percentage	amplitude_percentage	P_V	onset
71	-8.4400	98.629	1.306	.000	18.111
72	-10.2681	97.502	.712	.000	18.051
73	-13.2219	100.456	.849	.000	21.213
74	-11.8541	99.088	2.287	.000	21.258
75	-13.6778	97.021	.886	.000	21.198
76	-11.8541	94.742	3.931	.000	21.258
77	.0000	100.000	.908	.000	21.648
78	-14.1337	108.845	-.366	.000	21.183
79	-.4559	96.109	.542	.000	21.633
80	-12.0669	93.374	-.542	.000	21.251
81	.0000	.000	.000	.000	.000
82	-12.3100	99.514	1.970	.000	21.243
83	-2.7356	90.881	1.302	.000	21.558
84	-13.2219	100.456	.407	.000	21.213
85	-15.3257	104.310	.638	.000	24.525
86	-15.3257	103.831	2.176	.000	24.525
87	-20.8174	108.365	.666	.000	24.353
88	-19.8595	107.886	3.581	.000	24.383
89	.0000	100.000	.717	.000	25.005
90	-6.9604	96.903	-.275	.000	24.787
91	-7.4393	107.280	.541	.000	24.772
92	-21.9987	107.631	-.542	.000	24.316
93	.0000	.000	.000	.000	.000
94	-16.9860	106.450	1.809	.000	24.473
95	-14.3678	102.395	1.159	.000	24.555
96	-15.5492	103.129	.712	.000	24.518
97	-10.0966	93.649	.743	.000	27.815
98	-11.8818	95.405	2.122	.000	27.754
99	-11.4135	94.527	.666	.000	27.770
100	-11.8818	95.405	3.489	.000	27.754
101	.0000	100.000	.860	.000	28.160
102	-.4682	88.382	-.427	.000	28.144
103	-1.3169	98.010	.580	.000	28.115
104	-13.1694	92.098	-.505	.000	27.710
105	.0000	.000	.000	.000	.000

	pig_code	ID_gender	Pair_ID	Cycle_total	P_or_V	onset_ref
106	5564	2	9	7.000	1	0
107	5564	2	11	7.000	1	0
108	5564	2	11	7.000	1	0
109	5564	2	1	8.000	1	-1
110	5564	2	1	8.000	1	-1
111	5564	2	3	8.000	1	-1
112	5564	2	3	8.000	1	-1
113	5564	2	5	8.000	1	0
114	5564	2	6	8.000	2	0
115	5564	2	7	8.000	1	0
116	5564	2	8	8.000	2	-1
117	5564	2	9	8.000	0	0
118	5564	2	9	8.000	1	-1
119	5564	2	11	8.000	1	-1
120	5564	2	11	8.000	1	-1
121	5564	2	1	9.000	1	-1
122	5564	2	1	9.000	1	0
123	5564	2	3	9.000	1	0
124	5564	2	3	9.000	1	0
125	5564	2	5	9.000	1	0
126	5564	2	6	9.000	2	0
127	5564	2	7	9.000	1	0
128	5564	2	8	9.000	2	-1
129	5564	2	9	9.000	0	0
130	5564	2	9	9.000	1	0
131	5564	2	11	9.000	1	0
132	5564	2	11	9.000	1	0
133	5564	2	1	10.000	1	0
134	5564	2	1	10.000	1	0
135	5564	2	3	10.000	1	0
136	5564	2	3	10.000	1	0
137	5564	2	5	10.000	1	0
138	5564	2	6	10.000	2	0
139	5564	2	7	10.000	1	0
140	5564	2	8	10.000	2	0

	resp_duration	insp_duration	exp_duration	base_value	Peak_or_Valley	Amplitude
106	3	.659	2.601	-5.970	6.080	.110
107	3	.659	2.436	-6.810	6.900	.090
108	3	.644	2.630	-9.750	9.820	.070
109	3	.614	2.526	-9.340	9.420	.080
110	3	.614	2.660	-17.100	17.420	.320
111	3	.569	2.586	-17.900	18.040	.140
112	3	.569	2.691	-10.520	10.850	.330
113	3	.352	2.758	-20.770	20.920	.150
114	3	.525	2.570	-32.690	-32.790	.100
115	3	.262	2.938	-25.750	25.850	.100
116	3	.569	2.765	27.840	-27.700	.140
117	0	.000	.000	.000	.000	.000
118	3	.614	2.661	-5.980	6.060	.080
119	3	.719	2.555	-6.800	6.890	.090
120	3	.599	2.645	-9.750	9.810	.060
121	3	.787	2.525	-9.330	9.420	.090
122	3	.569	2.556	-17.080	17.510	.430
123	3	.614	2.586	-17.920	18.070	.150
124	3	.569	2.556	-10.520	10.940	.420
125	3	.322	2.743	-20.750	20.930	.180
126	3	.681	2.556	-32.650	-32.790	.140
127	3	.352	2.713	-25.710	25.860	.150
128	3	.794	2.436	27.830	-27.710	.120
129	0	.000	.000	.000	.000	.000
130	3	.569	2.551	-5.970	6.100	.130
131	3	.495	2.765	-6.810	6.900	.090
132	3	.540	2.615	-9.750	9.830	.080
133	3	.614	2.481	-9.330	9.440	.110
134	3	.614	2.601	-17.050	17.520	.470
135	3	.599	2.526	-17.900	18.100	.200
136	3	.689	2.526	-10.470	10.960	.490
137	3	.450	2.705	-20.730	20.920	.190
138	3	.607	2.690	-32.660	-32.760	.100
139	3	.435	2.547	-25.680	25.860	.180
140	3	.682	2.450	27.830	-27.720	.110

Sono respiration_Raw data.sav

	onset_percentage	duration_percentage	amplitude_percentage	P_V	onset
106	-11.8525	95.405	1.809	.000	27.755
107	-6.1457	90.577	1.304	.000	27.950
108	-11.4135	95.815	.713	.000	27.770
109	-16.5273	100.965	.849	.000	31.070
110	-19.0354	105.273	1.837	.000	30.992
111	-18.5531	96.365	.776	.000	31.007
112	-19.0354	104.823	3.041	.000	30.992
113	.0000	100.000	.717	.000	31.584
114	-10.8360	99.518	.305	.000	31.247
115	-2.8939	102.894	.387	.000	31.494
116	-23.6013	107.203	-.505	.000	30.850
117	.0000	.000	.000	.000	.000
118	-18.5531	105.305	1.320	.000	31.007
119	-17.1061	105.273	1.306	.000	31.052
120	-18.0707	104.309	.612	.000	31.022
121	-18.8254	108.059	.955	.000	34.132
122	-14.1925	101.958	2.456	.000	34.274
123	-14.6819	104.405	.830	.000	34.259
124	-14.1925	101.958	3.839	.000	34.274
125	.0000	100.000	.860	.000	34.709
126	-12.2349	105.612	-.427	.000	34.334
127	-.5220	100.000	.580	.000	34.693
128	-18.3361	105.383	-.433	.000	34.147
129	.0000	.000	.000	.000	.000
130	-14.6819	101.794	2.131	.000	34.259
131	-10.9951	106.362	1.304	.000	34.372
132	-14.6819	102.936	.814	.000	34.259
133	-9.7306	98.098	1.165	.000	37.459
134	-11.8859	101.902	2.683	.000	37.391
135	-9.0333	99.049	1.105	.000	37.481
136	-12.3613	101.902	4.471	.000	37.376
137	.0000	100.000	.908	.000	37.766
138	-6.1807	104.501	-.305	.000	37.571
139	-.2536	94.517	.696	.000	37.758
140	-12.5832	99.271	-.397	.000	37.369

Sono respiration_Raw data.sav

	pig_code	ID_gender	Pair_ID	Cycle_total	P_or_V	onset_ref
141	5564	2	9	10.000	0	0
142	5564	2	9	10.000	1	0
143	5564	2	11	10.000	1	0
144	5564	2	11	10.000	1	0
145	5564	2	1	11.000	1	0
146	5564	2	1	11.000	1	0
147	5564	2	3	11.000	1	0
148	5564	2	3	11.000	1	0
149	5564	2	5	11.000	1	0
150	5564	2	6	11.000	2	0
151	5564	2	7	11.000	1	0
152	5564	2	8	11.000	2	0
153	5564	2	9	11.000	0	0
154	5564	2	9	11.000	1	0
155	5564	2	11	11.000	1	0
156	5564	2	11	11.000	1	0
157	5564	2	1	12.000	1	0
158	5564	2	1	12.000	1	0
159	5564	2	3	12.000	1	0
160	5564	2	3	12.000	1	0
161	5564	2	5	12.000	1	0
162	5564	2	6	12.000	2	0
163	5564	2	7	12.000	1	0
164	5564	2	8	12.000	2	0
165	5564	2	9	12.000	0	0
166	5564	2	9	12.000	1	0
167	5564	2	11	12.000	1	0
168	5564	2	11	12.000	1	0
169	5564	2	1	13.000	1	0
170	5564	2	1	13.000	1	0
171	5564	2	3	13.000	1	0
172	5564	2	3	13.000	1	0
173	5564	2	5	13.000	1	0
174	5564	2	6	13.000	2	0
175	5564	2	7	13.000	1	0

Sono respiration_Raw data.sav

	resp_duration	insp_duration	exp_duration	base_value	Peak_or_Valley	Amplitude
141	0	.000	.000	.000	.000	.000
142	3	.689	2.556	-5.960	6.100	.140
143	3	.435	2.600	-6.800	6.920	.120
144	3	.659	2.571	-9.740	9.820	.080
145	3	.659	2.481	-9.340	9.420	.080
146	3	.644	2.353	-17.070	17.490	.420
147	3	40.538	-37.526	-17.910	18.090	.180
148	3	.532	2.465	-10.520	10.940	.420
149	3	.472	2.668	-20.760	20.910	.150
150	3	.397	2.773	-32.620	-32.750	.130
151	3	.622	2.668	-25.700	25.840	.140
152	4	.644	3.080	27.820	-27.660	.160
153	0	.000	.000	.000	.000	.000
154	3	.487	2.488	-5.980	6.090	.110
155	3	.689	2.233	-6.800	6.920	.120
156	3	.540	2.435	-9.730	9.800	.070
157	3	.712	2.450	-9.330	9.430	.100
158	3	.772	2.622	-17.020	17.470	.450
159	3	.809	2.451	-17.880	18.070	.190
160	3	.772	2.577	-10.460	10.900	.440
161	3	.525	2.727	-20.720	20.900	.180
162	3	.495	2.787	-32.630	-32.740	.110
163	3	.480	2.682	-25.690	25.840	.150
164	3	.510	2.705	27.810	-27.670	.140
165	0	.000	.000	.000	.000	.000
166	3	.757	2.592	-5.970	6.090	.120
167	3	.742	2.607	-6.790	6.880	.090
168	3	.779	2.630	-9.720	9.800	.080
169	3	.666	2.594	-9.340	9.420	.080
170	3	.547	2.593	-17.070	17.490	.420
171	3	.764	2.533	-17.900	18.060	.160
172	3	.637	2.555	-10.510	10.920	.410
173	3	.412	2.765	-20.730	20.920	.190
174	3	.382	2.743	-32.660	-32.760	.100
175	3	.502	2.847	-25.690	25.860	.170

Sono respiration_Raw data.sav

	onset_percentage	duration_percentage	amplitude_percentage	P_V	onset
141	.0000	.000	.000	.000	.000
142	-12.8368	102.853	2.295	.000	37.361
143	-4.2789	96.197	1.734	.000	37.631
144	-12.8368	102.377	.815	.000	37.361
145	-10.2866	100.000	.849	.000	40.583
146	-9.8089	95.446	2.401	.000	40.598
147	-10.2866	95.924	.995	.000	40.583
148	-10.2866	95.446	3.839	.000	40.583
149	.0000	100.000	.717	.000	40.906
150	-1.9108	100.955	-.397	.000	40.846
151	-4.7771	104.777	.542	.000	40.756
152	-12.6433	118.599	-.578	.000	40.509
153	.0000	.000	.000	.000	.000
154	-10.2866	94.745	1.806	.000	40.583
155	-7.4204	93.057	1.734	.000	40.673
156	-9.0764	94.745	.714	.000	40.621
157	-10.8549	97.232	1.060	.000	43.700
158	-14.0529	104.367	2.576	.000	43.596
159	-14.0529	100.246	1.051	.000	43.596
160	-14.0529	102.983	4.037	.000	43.596
161	.0000	100.000	.861	.000	44.053
162	-.7073	100.923	-.336	.000	44.030
163	.2153	97.232	.580	.000	44.060
164	-9.9016	98.862	-.506	.000	43.731
165	.0000	.000	.000	.000	.000
166	-14.0529	102.983	1.970	.000	43.596
167	-11.5314	102.983	1.308	.000	43.678
168	-14.0529	104.828	.816	.000	43.596
169	-12.5275	102.613	.849	.000	46.937
170	-10.8593	98.835	2.401	.000	46.990
171	-15.1086	103.777	.886	.000	46.855
172	-12.4961	100.472	3.755	.000	46.938
173	.0000	100.000	.908	.000	47.335
174	-.4721	98.363	-.305	.000	47.320
175	-3.7771	105.414	.657	.000	47.215

	pig_code	ID_gender	Pair_ID	Cycle_total	P_or_V	onset_ref
176	5564	2	8	13.000	2	0
177	5564	2	9	13.000	0	0
178	5564	2	9	13.000	1	0
179	5564	2	11	13.000	1	0
180	5564	2	11	13.000	1	0
181	5564	2	1	14.000	1	0
182	5564	2	1	14.000	1	0
183	5564	2	3	14.000	1	0
184	5564	2	3	14.000	1	0
185	5564	2	5	14.000	1	0
186	5564	2	6	14.000	2	0
187	5564	2	7	14.000	1	0
188	5564	2	8	14.000	2	0
189	5564	2	9	14.000	0	0
190	5564	2	9	14.000	1	0
191	5564	2	11	14.000	1	0
192	5564	2	11	14.000	1	0
193	5564	2	1	15.000	1	0
194	5564	2	1	15.000	1	0
195	5564	2	3	15.000	1	0
196	5564	2	3	15.000	1	0
197	5564	2	5	15.000	1	0
198	5564	2	6	15.000	2	0
199	5564	2	7	15.000	1	0
200	5564	2	8	15.000	2	-1
201	?	?	?	?	?	?
202	?	?	?	?	?	?
203	?	?	?	?	?	?
204	?	?	?	?	?	?
205	?	?	?	?	?	?
206	?	?	?	?	?	?
207	?	?	?	?	?	?
208	?	?	?	?	?	?
209	?	?	?	?	?	?
210	?	?	?	?	?	?

Sono respiration_Raw data.sav

	resp_duration	insp_duration	exp_duration	base_value	Peak_or_Valley	Amplitude
176	3	.240	2.945	27.810	-27.700	.110
177	0	.000	.000	-32.640	32.710	.070
178	3	.622	2.555	-5.980	6.100	.120
179	3	.555	2.705	-6.800	6.890	.090
180	3	.577	2.585	-9.730	9.810	.080
181	3	.622	2.518	-9.340	9.420	.080
182	3	.757	2.435	-17.120	17.470	.350
183	3	.674	2.458	-17.910	18.060	.150
184	3	.674	2.406	-10.530	10.910	.380
185	3	.397	2.795	-20.730	20.910	.180
186	3	.330	2.750	-32.640	-32.730	.090
187	3	.300	2.562	-25.710	25.850	.140
188	3	.315	2.600	27.810	-27.680	.130
189	0	.000	.000	.000	.000	.000
190	3	.674	2.436	-5.990	6.100	.110
191	3	.607	2.518	-6.810	6.900	.090
192	3	.622	2.555	-9.740	9.800	.060
193	3	.599	2.518	-9.340	9.410	.070
194	3	.644	2.586	-17.100	17.510	.410
195	3	.689	2.511	-17.920	18.080	.160
196	3	.629	2.571	-10.530	10.950	.420
197	3	.322	2.758	-20.750	20.900	.150
198	3	.397	2.623	-32.640	-32.710	.070
199	3	.577	2.698	-25.700	25.850	.150
200	3	.659	2.601	27.800	-27.670	.130
201	0	.000	.000	.000	?	?
202	3	.614	2.616	-5.990	?	?
203	3	.465	2.607	-6.810	?	?
204	3	.487	2.630	-9.740	?	?
205	0	.000	.000	.000	?	?
206	0	.000	.000	.000	?	?
207	0	.000	.000	.000	?	?
208	0	.000	.000	.000	?	?
209	2	.405	1.701	-18.810	?	?
210	0	.000	.000	.000	?	?

Sono respiration_Raw data.sav

	onset_percentage	duration_percentage	amplitude_percentage	P_V	onset
176	-11.5518	100.252	-.397	.000	46.968
177	-5.8117	98.052	.214	.000	53.510
178	-12.0239	100.000	1.967	.000	46.953
179	-9.4429	102.613	1.306	.000	47.035
180	-10.3872	99.528	.815	.000	47.005
181	-10.0877	98.371	.849	.000	50.190
182	-14.3170	100.000	2.003	.000	50.055
183	-11.7481	98.120	.831	.000	50.137
184	-11.7481	96.491	3.483	.000	50.137
185	.0000	100.000	.861	.000	50.512
186	-2.0990	96.491	.275	.000	50.445
187	2.5689	89.662	.542	.000	50.594
188	-11.7481	91.322	-.470	.000	50.137
189	.0000	.000	.000	.000	.000
190	-11.7481	97.431	1.803	.000	50.137
191	-6.5789	97.901	1.304	.000	50.302
192	-10.8083	99.530	.612	.000	50.167
193	-12.4026	101.201	.744	.000	53.307
194	-14.5779	104.870	2.342	.000	53.240
195	-15.0649	103.896	.885	.000	53.225
196	-14.5779	103.896	3.836	.000	53.240
197	.0000	100.000	.718	.000	53.689
198	-5.8117	98.052	.214	.000	53.510
199	-8.2792	106.331	.580	.000	53.434
200	-16.5260	105.844	-.470	.000	53.180
201	.0000	.000	.000	.000	.000
202	-14.1234	104.870	1.964	.000	53.254
203	-10.9740	99.740	1.304	.000	53.351
204	-10.4545	101.201	.612	.000	53.367
205	.0000	.000	.000	.000	.000
206	.0000	.000	.000	.000	.000
207	.0000	.000	.000	.000	.000
208	.0000	.000	.000	.000	.000
209	.0000	100.000	1.000	.000	3.529
210	.0000	.000	.000	.000	.000

Sono respiration_Raw data.sav

	pig_code	ID_gender	Pair_ID	Cycle_total	P_or_V	onset_ref
211	5773	2	7	1.000	1	0
212	5773	2	8	1.000	0	0
213	5773	2	9	1.000	0	0
214	5773	2	9	1.000	0	0
215	5773	2	11	1.000	1	0
216	5773	2	11	1.000	0	0
217	5773	2	1	2.000	0	0
218	5773	2	1	2.000	0	0
219	5773	2	3	2.000	0	0
220	5773	2	3	2.000	0	0
221	5773	2	5	2.000	1	0
222	5773	2	6	2.000	0	0
223	5773	2	7	2.000	1	1
224	5773	2	8	2.000	0	0
225	5773	2	9	2.000	0	0
226	5773	2	9	2.000	0	0
227	5773	2	11	2.000	1	0
228	5773	2	11	2.000	0	0
229	5773	2	1	3.000	0	0
230	5773	2	1	3.000	0	0
231	5773	2	3	3.000	0	0
232	5773	2	3	3.000	0	0
233	5773	2	5	3.000	1	0
234	5773	2	6	3.000	0	0
235	5773	2	7	3.000	1	0
236	5773	2	8	3.000	0	0
237	5773	2	9	3.000	0	0
238	5773	2	9	3.000	0	0
239	5773	2	11	3.000	1	0
240	5773	2	11	3.000	0	0
241	?	?	?	?	?	?
242	?	?	?	?	?	?
243	?	?	?	?	?	?
244	?	?	?	?	?	?
245	?	?	?	?	?	?

Sono respiration_Raw data.sav

	resp_duration	insp_duration	exp_duration	base_value	Peak_or_Valley	Amplitude
211	2	.457	1.986	-24.320	24.910	.590
212	0	.000	.000	.000	.000	.000
213	0	.000	.000	.000	.000	.000
214	0	.000	.000	.000	.000	.000
215	2	.689	1.633	-10.960	11.040	.080
216	0	.000	.000	.000	.000	.000
217	0	.000	.000	.000	.000	.000
218	0	.000	.000	.000	.000	.000
219	0	.000	.000	.000	.000	.000
220	0	.000	.000	.000	.000	.000
221	3	.877	1.798	-18.780	19.000	.220
222	0	.000	.000	.000	.000	.000
223	2	.375	1.985	-24.320	24.920	.600
224	0	.000	.000	.000	.000	.000
225	0	.000	.000	.000	.000	.000
226	0	.000	.000	.000	.000	.000
227	2	.734	1.694	-10.960	11.050	.090
228	0	.000	.000	.000	.000	.000
229	0	.000	.000	.000	.000	.000
230	0	.000	.000	.000	.000	.000
231	0	.000	.000	.000	.000	.000
232	0	.000	.000	.000	.000	.000
233	2	.659	1.754	-18.800	18.990	.190
234	0	.000	.000	.000	.000	.000
235	2	.487	1.823	-24.340	24.900	.560
236	0	.000	.000	.000	.000	.000
237	0	.000	.000	.000	.000	.000
238	0	.000	.000	.000	.000	.000
239	2	.712	1.499	-10.960	11.040	.080
240	0	.000	.000	.000	.000	.000
241	0	.000	.000	.000	?	?
242	0	.000	.000	.000	?	?
243	0	.000	.000	.000	?	?
244	0	.000	.000	.000	?	?
245	3	.832	1.723	-18.790	?	?

Sono respiration_Raw data.sav

	onset_percentage	duration_percentage	amplitude_percentage	P_V	onset
211	4.2735	116.002	2.369	.000	3.619
212	.0000	.000	.000	.000	.000
213	.0000	.000	.000	.000	.000
214	.0000	.000	.000	.000	.000
215	-20.9877	110.256	.725	.000	3.087
216	.0000	.000	.000	.000	.000
217	.0000	.000	.000	.000	.000
218	.0000	.000	.000	.000	.000
219	.0000	.000	.000	.000	.000
220	.0000	.000	.000	.000	.000
221	.0000	100.000	1.158	.000	5.620
222	.0000	.000	.000	.000	.000
223	18.7664	88.224	2.408	.000	6.122
224	.0000	.000	.000	.000	.000
225	.0000	.000	.000	.000	.000
226	.0000	.000	.000	.000	.000
227	-7.5514	90.766	.814	.000	5.418
228	.0000	.000	.000	.000	.000
229	.0000	.000	.000	.000	.000
230	.0000	.000	.000	.000	.000
231	.0000	.000	.000	.000	.000
232	.0000	.000	.000	.000	.000
233	.0000	100.000	1.001	.000	8.303
234	.0000	.000	.000	.000	.000
235	5.5533	95.731	2.249	.000	8.437
236	.0000	.000	.000	.000	.000
237	.0000	.000	.000	.000	.000
238	.0000	.000	.000	.000	.000
239	-18.6490	91.629	.725	.000	7.853
240	.0000	.000	.000	.000	.000
241	.0000	.000	.000	.000	.000
242	.0000	.000	.000	.000	.000
243	.0000	.000	.000	.000	.000
244	.0000	.000	.000	.000	.000
245	.0000	100.000	1.157	.000	10.281

Sono respiration_Raw data.sav

	pig_code	ID_gender	Pair_ID	Cycle_total	P_or_V	onset_ref
246	5773	2	6	4.000	0	0
247	5773	2	7	4.000	1	1
248	5773	2	8	4.000	0	0
249	5773	2	9	4.000	0	0
250	5773	2	9	4.000	0	0
251	5773	2	11	4.000	1	0
252	5773	2	11	4.000	0	0
253	5773	2	1	5.000	0	0
254	5773	2	1	5.000	0	0
255	5773	2	3	5.000	0	0
256	5773	2	3	5.000	0	0
257	5773	2	5	5.000	1	0
258	5773	2	6	5.000	0	0
259	5773	2	7	5.000	1	0
260	5773	2	8	5.000	0	0
261	5773	2	9	5.000	0	0
262	5773	2	9	5.000	0	0
263	5773	2	11	5.000	1	0
264	5773	2	11	5.000	0	0
265	5773	2	1	6.000	0	0
266	5773	2	1	6.000	0	0
267	5773	2	3	6.000	0	0
268	5773	2	3	6.000	0	0
269	5773	2	5	6.000	1	0
270	5773	2	6	6.000	0	0
271	5773	2	7	6.000	1	0
272	5773	2	8	6.000	0	0
273	5773	2	9	6.000	0	0
274	5773	2	9	6.000	0	0
275	5773	2	11	6.000	1	-1
276	5773	2	11	6.000	0	0
277	5773	2	1	7.000	0	0
278	5773	2	1	7.000	0	0
279	5773	2	3	7.000	0	0
280	5773	2	3	7.000	0	0

	resp_duration	insp_duration	exp_duration	base_value	Peak_or_Valley	Amplitude
246	0	.000	.000	.000	.000	.000
247	2	.390	1.970	-24.220	24.940	.720
248	0	.000	.000	.000	.000	.000
249	0	.000	.000	.000	.000	.000
250	0	.000	.000	.000	.000	.000
251	2	.704	1.686	-10.960	11.070	.110
252	0	.000	.000	.000	.000	.000
253	0	.000	.000	.000	.000	.000
254	0	.000	.000	.000	.000	.000
255	0	.000	.000	.000	.000	.000
256	0	.000	.000	.000	.000	.000
257	3	.741	1.784	-18.790	18.990	.200
258	0	.000	.000	.000	.000	.000
259	2	.405	1.798	-24.320	24.900	.580
260	0	.000	.000	.000	.000	.000
261	0	.000	.000	.000	.000	.000
262	0	.000	.000	.000	.000	.000
263	2	.772	1.491	-10.960	11.050	.090
264	0	.000	.000	.000	.000	.000
265	0	.000	.000	.000	.000	.000
266	0	.000	.000	.000	.000	.000
267	0	.000	.000	.000	.000	.000
268	0	.000	.000	.000	.000	.000
269	2	.247	1.844	-18.840	19.030	.190
270	0	.000	.000	.000	.000	.000
271	2	.405	1.940	-24.310	24.910	.600
272	0	.000	.000	.000	.000	.000
273	0	.000	.000	.000	.000	.000
274	0	.000	.000	.000	.000	.000
275	2	.727	1.633	-10.970	11.060	.090
276	0	.000	.000	.000	.000	.000
277	0	.000	.000	.000	.000	.000
278	0	.000	.000	.000	.000	.000
279	0	.000	.000	.000	.000	.000
280	0	.000	.000	.000	.000	.000

	onset_percentage	duration_percentage	amplitude_percentage	P_V	onset
246	.0000	.000	.000	.000	.000
247	19.6086	92.368	2.887	.000	10.782
248	.0000	.000	.000	.000	.000
249	.0000	.000	.000	.000	.000
250	.0000	.000	.000	.000	.000
251	-8.8063	93.542	.994	.000	10.056
252	.0000	.000	.000	.000	.000
253	.0000	.000	.000	.000	.000
254	.0000	.000	.000	.000	.000
255	.0000	.000	.000	.000	.000
256	.0000	.000	.000	.000	.000
257	.0000	100.000	1.053	.000	12.843
258	.0000	.000	.000	.000	.000
259	12.4752	87.248	2.329	.000	13.158
260	.0000	.000	.000	.000	.000
261	.0000	.000	.000	.000	.000
262	.0000	.000	.000	.000	.000
263	-16.3168	89.624	.814	.000	12.431
264	.0000	.000	.000	.000	.000
265	.0000	.000	.000	.000	.000
266	.0000	.000	.000	.000	.000
267	.0000	.000	.000	.000	.000
268	.0000	.000	.000	.000	.000
269	.0000	100.000	.998	.000	15.361
270	.0000	.000	.000	.000	.000
271	.0000	112.147	2.409	.000	15.361
272	.0000	.000	.000	.000	.000
273	.0000	.000	.000	.000	.000
274	.0000	.000	.000	.000	.000
275	-32.2334	112.865	.814	.000	14.687
276	.0000	.000	.000	.000	.000
277	.0000	.000	.000	.000	.000
278	.0000	.000	.000	.000	.000
279	.0000	.000	.000	.000	.000
280	.0000	.000	.000	.000	.000

Sono respiration_Raw data.sav

	pig_code	ID_gender	Pair_ID	Cycle_total	P_or_V	onset_ref
281	5773	2	5	7.000	1	0
282	5773	2	6	7.000	0	0
283	5773	2	7	7.000	1	0
284	5773	2	8	7.000	0	0
285	5773	2	9	7.000	0	0
286	5773	2	9	7.000	0	0
287	5773	2	11	7.000	1	0
288	5773	2	11	7.000	0	0
289	5773	2	1	8.000	0	0
290	5773	2	1	8.000	0	0
291	5773	2	3	8.000	0	0
292	5773	2	3	8.000	0	0
293	5773	2	5	8.000	1	0
294	5773	2	6	8.000	0	0
295	5773	2	7	8.000	1	0
296	5773	2	8	8.000	0	0
297	5773	2	9	8.000	0	0
298	5773	2	9	8.000	0	0
299	5773	2	11	8.000	1	0
300	5773	2	11	8.000	0	0
301	5773	2	1	9.000	0	0
302	5773	2	1	9.000	0	0
303	5773	2	3	9.000	0	0
304	5773	2	3	9.000	0	0
305	5773	2	5	9.000	1	0
306	5773	2	6	9.000	0	0
307	5773	2	7	9.000	1	0
308	5773	2	8	9.000	0	0
309	5773	2	9	9.000	0	0
310	5773	2	9	9.000	0	0
311	5773	2	11	9.000	1	-1
312	5773	2	11	9.000	0	0
313	5773	2	1	10.000	0	0
314	5773	2	1	10.000	0	0
315	5773	2	3	10.000	0	0

Sono respiration_Raw data.sav

	resp_duration	insp_duration	exp_duration	base_value	Peak_or_Valley	Amplitude
281	3	.660	1.873	-18.800	18.980	.180
282	0	.000	.000	.000	.000	.000
283	2	.405	1.925	-24.330	24.900	.570
284	0	.000	.000	.000	.000	.000
285	0	.000	.000	.000	.000	.000
286	0	.000	.000	.000	.000	.000
287	2	.734	1.619	-10.960	11.040	.080
288	0	.000	.000	.000	.000	.000
289	0	.000	.000	.000	.000	.000
290	0	.000	.000	.000	.000	.000
291	0	.000	.000	.000	.000	.000
292	0	.000	.000	.000	.000	.000
293	3	.787	1.761	-18.780	19.000	.220
294	0	.000	.000	.000	.000	.000
295	2	.405	1.828	-24.280	24.920	.640
296	0	.000	.000	.000	.000	.000
297	0	.000	.000	.000	.000	.000
298	0	.000	.000	.000	.000	.000
299	2	.727	1.551	-10.970	11.060	.090
300	0	.000	.000	.000	.000	.000
301	0	.000	.000	.000	.000	.000
302	0	.000	.000	.000	.000	.000
303	0	.000	.000	.000	.000	.000
304	0	.000	.000	.000	.000	.000
305	2	.360	1.723	-18.820	19.010	.190
306	0	.000	.000	.000	.000	.000
307	2	.405	2.083	-24.320	24.900	.580
308	0	.000	.000	.000	.000	.000
309	0	.000	.000	.000	.000	.000
310	0	.000	.000	.000	.000	.000
311	2	.712	1.723	-10.960	11.050	.090
312	0	.000	.000	.000	.000	.000
313	0	.000	.000	.000	.000	.000
314	0	.000	.000	.000	.000	.000
315	0	.000	.000	.000	.000	.000

Sono respiration_Raw data.sav

	onset_percentage	duration_percentage	amplitude_percentage	P_V	onset
281	.0000	100.000	.948	.000	17.444
282	.0000	.000	.000	.000	.000
283	9.4749	91.986	2.289	.000	17.684
284	.0000	.000	.000	.000	.000
285	.0000	.000	.000	.000	.000
286	.0000	.000	.000	.000	.000
287	-15.9495	92.894	.725	.000	17.040
288	.0000	.000	.000	.000	.000
289	.0000	.000	.000	.000	.000
290	.0000	.000	.000	.000	.000
291	.0000	.000	.000	.000	.000
292	.0000	.000	.000	.000	.000
293	.0000	100.000	1.158	.000	22.045
294	.0000	.000	.000	.000	.000
295	16.1695	87.637	2.568	.000	22.457
296	.0000	.000	.000	.000	.000
297	.0000	.000	.000	.000	.000
298	.0000	.000	.000	.000	.000
299	-9.4192	89.403	.814	.000	21.805
300	.0000	.000	.000	.000	.000
301	.0000	.000	.000	.000	.000
302	.0000	.000	.000	.000	.000
303	.0000	.000	.000	.000	.000
304	.0000	.000	.000	.000	.000
305	.0000	100.000	.999	.000	24.585
306	.0000	.000	.000	.000	.000
307	6.1450	119.443	2.329	.000	24.713
308	.0000	.000	.000	.000	.000
309	.0000	.000	.000	.000	.000
310	.0000	.000	.000	.000	.000
311	-24.4359	116.899	.814	.000	24.076
312	.0000	.000	.000	.000	.000
313	.0000	.000	.000	.000	.000
314	.0000	.000	.000	.000	.000
315	.0000	.000	.000	.000	.000

	pig_code	ID_gender	Pair_ID	Cycle_total	P_or_V	onset_ref
316	5773	2	3	10.000	0	0
317	5773	2	5	10.000	1	0
318	5773	2	6	10.000	0	0
319	5773	2	7	10.000	1	1
320	5773	2	8	10.000	0	0
321	?	2	9	10.000	0	0
322	5773	2	9	10.000	0	0
323	5773	2	11	10.000	1	0
324	5773	2	11	10.000	0	0
325	5773	2	1	11.000	0	0
326	5773	2	1	11.000	0	0
327	5773	2	3	11.000	0	0
328	5773	2	3	11.000	0	0
329	5773	2	5	11.000	1	0
330	5773	2	6	11.000	0	0
331	5773	2	7	11.000	1	0
332	5773	2	8	11.000	0	0
333	5773	2	9	11.000	0	0
334	5773	2	9	11.000	0	0
335	5773	2	11	11.000	1	0
336	5773	2	11	11.000	0	0
337	5773	2	1	12.000	0	0
338	5773	2	1	12.000	0	0
339	5773	2	3	12.000	0	0
340	5773	2	3	12.000	0	0
341	?	?	?	?	?	?
342	?	?	?	?	?	?
343	?	?	?	?	?	?
344	?	?	?	?	?	?
345	?	?	?	?	?	?
346	?	?	?	?	?	?
347	?	?	?	?	?	?
348	?	?	?	?	?	?
349	?	?	?	?	?	?
350	?	?	?	?	?	?

	resp_duration	insp_duration	exp_duration	base_value	Peak_or_Valley	Amplitude
316	0	.000	.000	.000	.000	.000
317	3	26.676	-24.016	-18.800	19.010	.210
318	0	.000	.000	.000	.000	.000
319	2	.367	1.873	-24.310	24.910	.600
320	0	.000	.000	.000	.000	.000
321	0	.000	.000	.000	.000	.000
322	0	.000	.000	.000	.000	.000
323	2	.757	1.558	-10.970	11.050	.080
324	0	.000	.000	.000	.000	.000
325	0	.000	.000	.000	.000	.000
326	0	.000	.000	.000	.000	.000
327	0	.000	.000	.000	.000	.000
328	0	.000	.000	.000	.000	.000
329	2	.337	1.761	-18.820	19.010	.190
330	0	.000	.000	.000	.000	.000
331	2	.510	1.910	-24.300	24.890	.590
332	0	.000	.000	.000	.000	.000
333	0	.000	.000	.000	.000	.000
334	0	.000	.000	.000	.000	.000
335	2	.629	1.634	-10.970	11.040	.070
336	0	.000	.000	.000	.000	.000
337	0	.000	.000	.000	.000	.000
338	0	.000	.000	.000	.000	.000
339	0	.000	.000	.000	.000	.000
340	0	.000	.000	.000	.000	.000
341	2	.802	1.296	-18.770	?	?
342	0	.000	.000	.000	?	?
343	2	.435	1.798	-24.300	?	?
344	0	.000	.000	.000	?	?
345	0	.000	.000	.000	?	?
346	0	.000	.000	.000	?	?
347	2	.712	1.551	-10.970	?	?
348	0	.000	.000	.000	?	?
349	0	.000	.000	.000	?	?
350	0	.000	.000	.000	?	?

Sono respiration_Raw data.sav

	onset_percentage	duration_percentage	amplitude_percentage	P_V	onset
316	.0000	.000	.000	.000	.000
317	.0000	100.000	1.105	.000	26.676
318	.0000	.000	.000	.000	.000
319	18.8722	84.211	2.409	.000	27.178
320	.0000	.000	.000	.000	.000
321	.0000	.000	.000	.000	.000
322	.0000	.000	.000	.000	.000
323	-5.3383	87.030	.724	.000	26.534
324	.0000	.000	.000	.000	.000
325	.0000	.000	.000	.000	.000
326	.0000	.000	.000	.000	.000
327	.0000	.000	.000	.000	.000
328	.0000	.000	.000	.000	.000
329	.0000	100.000	.999	.000	29.359
330	.0000	.000	.000	.000	.000
331	2.4786	115.348	2.370	.000	29.411
332	.0000	.000	.000	.000	.000
333	.0000	.000	.000	.000	.000
334	.0000	.000	.000	.000	.000
335	-23.2602	107.865	.634	.000	28.871
336	.0000	.000	.000	.000	.000
337	.0000	.000	.000	.000	.000
338	.0000	.000	.000	.000	.000
339	.0000	.000	.000	.000	.000
340	.0000	.000	.000	.000	.000
341	.0000	100.000	1.159	.000	31.449
342	.0000	.000	.000	.000	.000
343	18.2078	106.435	2.370	.000	31.831
344	.0000	.000	.000	.000	.000
345	.0000	.000	.000	.000	.000
346	.0000	.000	.000	.000	.000
347	-13.9180	107.865	.724	.000	31.157
348	.0000	.000	.000	.000	.000
349	.0000	.000	.000	.000	.000
350	.0000	.000	.000	.000	.000

Sono respiration_Raw data.sav

	pig_code	ID_gender	Pair_ID	Cycle_total	P_or_V	onset_ref
351	5773	2	3	13.000	0	0
352	5773	2	3	13.000	0	0
353	5773	2	5	13.000	1	0
354	5773	2	6	13.000	0	0
355	5773	2	7	13.000	1	1
356	5773	2	8	13.000	0	0
357	5773	2	9	13.000	0	0
358	5773	2	9	13.000	0	0
359	5773	2	11	13.000	1	0
360	5773	2	11	13.000	0	0
361	5773	2	1	14.000	0	0
362	5773	2	1	14.000	0	0
363	5773	2	3	14.000	0	0
364	5773	2	3	14.000	0	0
365	5773	2	5	14.000	1	0
366	5773	2	6	14.000	0	0
367	5773	2	7	14.000	1	0
368	5773	2	8	14.000	0	0
369	5773	2	9	14.000	0	0
370	5773	2	9	14.000	0	0
371	5773	2	11	14.000	1	0
372	5773	2	11	14.000	0	0
373	5773	2	1	15.000	0	0
374	5773	2	1	15.000	0	0
375	5773	2	3	15.000	0	0
376	5773	2	3	15.000	0	0
377	5773	2	5	15.000	1	0
378	5773	2	6	15.000	0	0
379	5773	2	7	15.000	1	0
380	5773	2	8	15.000	0	0
381	5773	2	9	15.000	0	0
382	5773	2	9	15.000	0	0
383	5773	2	11	15.000	1	0
384	5773	2	11	15.000	0	0
385	5824	1	1	1.000	0	0

	resp_duration	insp_duration	exp_duration	base_value	Peak_or_Valley	Amplitude
351	0	.000	.000	.000	.000	.000
352	0	.000	.000	.000	.000	.000
353	3	.757	1.768	-18.800	19.020	.220
354	0	.000	.000	.000	.000	.000
355	2	.412	2.016	-24.310	24.890	.580
356	0	.000	.000	.000	.000	.000
357	0	.000	.000	.000	.000	.000
358	0	.000	.000	.000	.000	.000
359	2	.712	1.731	-10.970	11.050	.080
360	0	.000	.000	.000	.000	.000
361	0	.000	.000	.000	.000	.000
362	0	.000	.000	.000	.000	.000
363	0	.000	.000	.000	.000	.000
364	0	.000	.000	.000	.000	.000
365	2	.847	1.596	-18.800	19.020	.220
366	0	.000	.000	.000	.000	.000
367	2	.397	1.814	-24.200	24.940	.740
368	0	.000	.000	.000	.000	.000
369	0	.000	.000	.000	.000	.000
370	0	.000	.000	.000	.000	.000
371	2	.697	1.558	-10.970	11.070	.100
372	0	.000	.000	.000	.000	.000
373	0	.000	.000	.000	.000	.000
374	0	.000	.000	.000	.000	.000
375	0	.000	.000	.000	.000	.000
376	0	.000	.000	.000	.000	.000
377	2	.712	1.311	-18.810	19.000	.190
378	0	.000	.000	.000	.000	.000
379	2	.465	1.843	-24.310	24.910	.600
380	0	.000	.000	.000	.000	.000
381	0	.000	.000	.000	?	?
382	0	.000	.000	.000	?	?
383	2	.697	1.581	-10.960	?	?
384	0	.000	.000	.000	?	?
385	0	.000	.000	.000	?	?

	onset_percentage	duration_percentage	amplitude_percentage	P_V	onset
351	.0000	.000	.000	.000	.000
352	.0000	.000	.000	.000	.000
353	.0000	100.000	1.157	.000	33.540
354	.0000	.000	.000	.000	.000
355	21.6634	96.158	2.330	.000	34.087
356	.0000	.000	.000	.000	.000
357	.0000	.000	.000	.000	.000
358	.0000	.000	.000	.000	.000
359	-4.7525	96.752	.724	.000	33.420
360	.0000	.000	.000	.000	.000
361	.0000	.000	.000	.000	.000
362	.0000	.000	.000	.000	.000
363	.0000	.000	.000	.000	.000
364	.0000	.000	.000	.000	.000
365	.0000	100.000	1.157	.000	36.080
366	.0000	.000	.000	.000	.000
367	17.1920	90.503	2.967	.000	36.500
368	.0000	.000	.000	.000	.000
369	.0000	.000	.000	.000	.000
370	.0000	.000	.000	.000	.000
371	-8.8825	92.305	.903	.000	35.863
372	.0000	.000	.000	.000	.000
373	.0000	.000	.000	.000	.000
374	.0000	.000	.000	.000	.000
375	.0000	.000	.000	.000	.000
376	.0000	.000	.000	.000	.000
377	.0000	100.000	1.000	.000	38.500
378	.0000	.000	.000	.000	.000
379	10.7761	114.088	2.409	.000	38.718
380	.0000	.000	.000	.000	.000
381	.0000	.000	.000	.000	.000
382	.0000	.000	.000	.000	.000
383	-18.4874	112.605	.725	.000	38.126
384	.0000	.000	.000	.000	.000
385	.0000	.000	.000	.000	.000

Sono respiration_Raw data.sav

	pig_code	ID_gender	Pair_ID	Cycle_total	P_or_V	onset_ref
386	5824	1	1	1.000	0	0
387	5824	1	3	1.000	0	0
388	5824	1	3	1.000	0	0
389	5824	1	5	1.000	1	0
390	5824	1	6	1.000	2	0
391	5824	1	7	1.000	0	0
392	5824	1	8	1.000	2	0
393	5824	1	9	1.000	0	0
394	5824	1	9	1.000	0	0
395	5824	1	11	1.000	1	0
396	5824	1	11	1.000	0	0
397	5824	1	1	2.000	0	0
398	5824	1	1	2.000	0	0
399	5824	1	3	2.000	0	0
400	5824	1	3	2.000	0	0
401	5824	1	5	2.000	1	0
402	5824	1	6	2.000	2	0
403	5824	1	7	2.000	0	0
404	5824	1	8	2.000	2	0
405	5824	1	9	2.000	0	0
406	5824	1	9	2.000	0	0
407	5824	1	11	2.000	1	0
408	5824	1	11	2.000	0	0
409	5824	1	1	3.000	0	0
410	5824	1	1	3.000	0	0
411	5824	1	3	3.000	0	0
412	5824	1	3	3.000	0	0
413	5824	1	5	3.000	1	0
414	5824	1	6	3.000	2	0
415	5824	1	7	3.000	0	0
416	5824	1	8	3.000	2	0
417	5824	1	9	3.000	0	0
418	5824	1	9	3.000	0	0
419	5824	1	11	3.000	1	0
420	5824	1	11	3.000	0	0

Sono respiration_Raw data.sav

	resp_duration	insp_duration	exp_duration	base_value	Peak_or_Valley	Amplitude
386	0	.000	.000	.000	.000	.000
387	0	.000	.000	.000	.000	.000
388	0	.000	.000	.000	.000	.000
389	2	.944	.749	-30.210	30.230	.020
390	2	.922	.659	33.120	-32.990	.130
391	0	.000	.000	.000	.000	.000
392	2	.994	.699	33.320	-33.270	.050
393	0	.000	.000	.000	.000	.000
394	0	.000	.000	.000	.000	.000
395	2	.989	.682	7.340	7.300	.040
396	0	.000	.000	.000	.000	.000
397	0	.000	.000	.000	.000	.000
398	0	.000	.000	.000	.000	.000
399	0	.000	.000	.000	.000	.000
400	0	.000	.000	.000	.000	.000
401	2	.862	1.094	-30.210	30.250	.040
402	2	.652	1.191	33.130	-33.060	.070
403	0	.000	.000	.000	.000	.000
404	2	1.079	.824	33.310	-33.270	.040
405	0	.000	.000	.000	.000	.000
406	0	.000	.000	.000	.000	.000
407	2	1.266	.690	7.340	7.320	.020
408	0	.000	.000	.000	.000	.000
409	0	.000	.000	.000	.000	.000
410	0	.000	.000	.000	.000	.000
411	0	.000	.000	.000	.000	.000
412	0	.000	.000	.000	.000	.000
413	2	.944	.997	-30.210	30.250	.040
414	2	.794	.907	7.340	-7.320	.020
415	0	.000	.000	.000	.000	.000
416	2	.996	.795	33.310	-33.270	.040
417	0	.000	.000	.000	.000	.000
418	0	.000	.000	.000	.000	.000
419	2	.959	.922	7.340	7.320	.020
420	0	.000	.000	.000	.000	.000

Sono respiration_Raw data.sav

	onset_percentage	duration_percentage	amplitude_percentage	P_V	onset
386	.0000	.000	.000	.000	.000
387	.0000	.000	.000	.000	.000
388	.0000	.000	.000	.000	.000
389	.0000	100.000	.066	.000	1.888
390	-3.9575	93.385	-.394	.000	1.821
391	.0000	.000	.000	.000	.000
392	-3.9575	100.000	-.150	.000	1.821
393	.0000	.000	.000	.000	.000
394	.0000	.000	.000	.000	.000
395	-1.7720	98.701	.548	.000	1.858
396	.0000	.000	.000	.000	.000
397	.0000	.000	.000	.000	.000
398	.0000	.000	.000	.000	.000
399	.0000	.000	.000	.000	.000
400	.0000	.000	.000	.000	.000
401	.0000	100.000	.132	.000	4.016
402	-10.3272	94.223	-.212	.000	3.814
403	.0000	.000	.000	.000	.000
404	-6.8507	97.290	-.120	.000	3.882
405	.0000	.000	.000	.000	.000
406	.0000	.000	.000	.000	.000
407	-6.8507	100.000	.273	.000	3.882
408	.0000	.000	.000	.000	.000
409	.0000	.000	.000	.000	.000
410	.0000	.000	.000	.000	.000
411	.0000	.000	.000	.000	.000
412	.0000	.000	.000	.000	.000
413	.0000	100.000	.132	.000	6.219
414	-2.3184	87.635	-.273	.000	6.174
415	.0000	.000	.000	.000	.000
416	-3.0912	92.272	-.120	.000	6.159
417	.0000	.000	.000	.000	.000
418	.0000	.000	.000	.000	.000
419	-5.7702	96.909	.273	.000	6.107
420	.0000	.000	.000	.000	.000

Sono respiration_Raw data.sav

	pig_code	ID_gender	Pair_ID	Cycle_total	P_or_V	onset_ref
421	5824	1	1	4.000	0	0
422	5824	1	1	4.000	0	0
423	5824	1	3	4.000	0	0
424	5824	1	3	4.000	0	0
425	5824	1	5	4.000	1	0
426	5824	1	6	4.000	2	0
427	5824	1	7	4.000	0	0
428	5824	1	8	4.000	2	0
429	5824	1	9	4.000	0	0
430	5824	1	9	4.000	0	0
431	5824	1	11	4.000	1	0
432	5824	1	11	4.000	0	0
433	5824	1	1	5.000	0	0
434	5824	1	1	5.000	0	0
435	5824	1	3	5.000	0	0
436	5824	1	3	5.000	0	0
437	5824	1	5	5.000	1	0
438	5824	1	6	5.000	2	0
439	5824	1	7	5.000	0	0
440	5824	1	8	5.000	2	0
441	5824	1	9	5.000	0	0
442	5824	1	9	5.000	0	0
443	5824	1	11	5.000	1	0
444	5824	1	11	5.000	0	0
445	5824	1	1	6.000	0	0
446	5824	1	1	6.000	0	0
447	5824	1	3	6.000	0	0
448	5824	1	3	6.000	0	0
449	5824	1	5	6.000	1	0
450	5824	1	6	6.000	2	0
451	5824	1	7	6.000	0	0
452	5824	1	8	6.000	2	0
453	5824	1	9	6.000	0	0
454	5824	1	9	6.000	0	0
455	5824	1	11	6.000	1	0

	resp_duration	insp_duration	exp_duration	base_value	Peak_or_Valley	Amplitude
421	0	.000	.000	.000	.000	.000
422	0	.000	.000	.000	.000	.000
423	0	.000	.000	.000	.000	.000
424	0	.000	.000	.000	.000	.000
425	2	.906	1.245	-30.210	30.230	.020
426	1	.974	.502	33.120	-32.970	.150
427	0	.000	.000	.000	.000	.000
428	2	1.169	.771	33.320	-33.250	.070
429	0	.000	.000	.000	.000	.000
430	0	.000	.000	.000	.000	.000
431	2	.779	1.072	7.340	7.300	.040
432	0	.000	.000	.000	.000	.000
433	0	.000	.000	.000	.000	.000
434	0	.000	.000	.000	.000	.000
435	0	.000	.000	.000	.000	.000
436	0	.000	.000	.000	.000	.000
437	2	.959	.914	-30.210	30.230	.020
438	2	.944	.660	33.120	-32.970	.150
439	0	.000	.000	.000	.000	.000
440	2	.967	.689	33.320	-33.250	.070
441	0	.000	.000	.000	.000	.000
442	0	.000	.000	.000	.000	.000
443	2	.899	.764	7.340	7.300	.040
444	0	.000	.000	.000	.000	.000
445	0	.000	.000	.000	.000	.000
446	0	.000	.000	.000	.000	.000
447	0	.000	.000	.000	.000	.000
448	0	.000	.000	.000	.000	.000
449	2	.922	1.161	-30.200	30.230	.030
450	2	.495	1.056	33.070	-32.970	.100
451	0	.000	.000	.000	.000	.000
452	2	1.229	.704	33.310	-33.260	.050
453	0	.000	.000	.000	.000	.000
454	0	.000	.000	.000	.000	.000
455	2	1.424	.614	7.340	7.320	.020

	onset_percentage	duration_percentage	amplitude_percentage	P_V	onset
421	.0000	.000	.000	.000	.000
422	.0000	.000	.000	.000	.000
423	.0000	.000	.000	.000	.000
424	.0000	.000	.000	.000	.000
425	.0000	100.000	.066	.000	8.415
426	.4649	68.619	-.455	.000	8.415
427	.0000	.000	.000	.000	.000
428	.6974	90.191	-.211	.000	8.430
429	.0000	.000	.000	.000	.000
430	.0000	.000	.000	.000	.000
431	.6974	86.053	.548	.000	8.430
432	.0000	.000	.000	.000	.000
433	.0000	.000	.000	.000	.000
434	.0000	.000	.000	.000	.000
435	.0000	.000	.000	.000	.000
436	.0000	.000	.000	.000	.000
437	.0000	100.000	.066	.000	10.768
438	-1.6017	85.638	-.455	.000	10.738
439	.0000	.000	.000	.000	.000
440	.5339	88.414	-.211	.000	10.768
441	.0000	.000	.000	.000	.000
442	.0000	.000	.000	.000	.000
443	.5339	88.788	.548	.000	10.768
444	.0000	.000	.000	.000	.000
445	.0000	.000	.000	.000	.000
446	.0000	.000	.000	.000	.000
447	.0000	.000	.000	.000	.000
448	.0000	.000	.000	.000	.000
449	.0000	100.000	.099	.000	12.986
450	7.9213	74.460	-.303	.000	13.151
451	.0000	.000	.000	.000	.000
452	-7.2012	92.799	-.150	.000	12.836
453	.0000	.000	.000	.000	.000
454	.0000	.000	.000	.000	.000
455	-9.7456	97.840	.273	.000	12.783

	pig_code	ID_gender	Pair_ID	Cycle_total	P_or_V	onset_ref
456	5824	1	11	6.000	0	0
457	5824	1	1	7.000	0	0
458	5824	1	1	7.000	0	0
459	5824	1	3	7.000	0	0
460	5824	1	3	7.000	0	0
461	5824	1	5	7.000	1	0
462	5824	1	6	7.000	2	0
463	5824	1	7	7.000	0	0
464	5824	1	8	7.000	2	0
465	5824	1	9	7.000	0	0
466	5824	1	9	7.000	0	0
467	5824	1	11	7.000	1	0
468	5824	1	11	7.000	0	0
469	5824	1	1	8.000	0	0
470	5824	1	1	8.000	0	0
471	5824	1	3	8.000	0	0
472	5824	1	3	8.000	0	0
473	5824	1	5	8.000	1	0
474	5824	1	6	8.000	2	0
475	5824	1	7	8.000	0	0
476	5824	1	8	8.000	2	0
477	5824	1	9	8.000	0	0
478	5824	1	9	8.000	0	0
479	5824	1	11	8.000	1	0
480	5824	1	11	8.000	0	0
481	?	?	?	?	?	?
482	?	?	?	?	?	?
483	?	?	?	?	?	?
484	?	?	?	?	?	?
485	?	?	?	?	?	?
486	?	?	?	?	?	?
487	?	?	?	?	?	?
488	?	?	?	?	?	?
489	?	?	?	?	?	?
490	?	?	?	?	?	?

	resp_duration	insp_duration	exp_duration	base_value	Peak_or_Valley	Amplitude
456	0	.000	.000	.000	.000	.000
457	0	.000	.000	.000	.000	.000
458	0	.000	.000	.000	.000	.000
459	0	.000	.000	.000	.000	.000
460	0	.000	.000	.000	.000	.000
461	2	1.094	.809	-30.200	30.230	.030
462	1	.577	.906	33.040	-32.900	.140
463	0	.000	.000	.000	.000	.000
464	2	1.176	.645	33.320	-33.270	.050
465	0	.000	.000	.000	.000	.000
466	0	.000	.000	.000	.000	.000
467	2	1.251	.525	7.350	7.320	.030
468	0	.000	.000	.000	.000	.000
469	0	.000	.000	.000	.000	.000
470	0	.000	.000	.000	.000	.000
471	0	.000	.000	.000	.000	.000
472	0	.000	.000	.000	.000	.000
473	2	1.206	.847	-30.200	30.220	.020
474	2	.847	.704	33.120	-32.990	.130
475	0	.000	.000	.000	.000	.000
476	2	1.206	.795	33.320	-33.270	.050
477	0	.000	.000	.000	.000	.000
478	0	.000	.000	.000	.000	.000
479	2	.652	1.019	7.340	7.310	.030
480	0	.000	.000	.000	.000	.000
481	0	.000	.000	.000	?	?
482	0	.000	.000	.000	?	?
483	0	.000	.000	.000	?	?
484	0	.000	.000	.000	?	?
485	2	1.138	.833	-30.190	?	?
486	1	.862	.569	33.120	?	?
487	0	.000	.000	.000	?	?
488	2	1.050	.793	33.320	?	?
489	0	.000	.000	.000	?	?
490	0	.000	.000	.000	?	?

Sono respiration_Raw data.sav

	onset_percentage	duration_percentage	amplitude_percentage	P_V	onset
456	.0000	.000	.000	.000	.000
457	.0000	.000	.000	.000	.000
458	.0000	.000	.000	.000	.000
459	.0000	.000	.000	.000	.000
460	.0000	.000	.000	.000	.000
461	.0000	100.000	.099	.000	15.264
462	-7.0941	77.930	-.426	.000	15.129
463	.0000	.000	.000	.000	.000
464	.5255	95.691	-.150	.000	15.166
465	.0000	.000	.000	.000	.000
466	.0000	.000	.000	.000	.000
467	-7.0941	93.326	.410	.000	15.129
468	.0000	.000	.000	.000	.000
469	.0000	.000	.000	.000	.000
470	.0000	.000	.000	.000	.000
471	.0000	.000	.000	.000	.000
472	.0000	.000	.000	.000	.000
473	.0000	100.000	.066	.000	17.399
474	-5.8451	75.548	-.394	.000	17.279
475	.0000	.000	.000	.000	.000
476	-5.1145	97.467	-.150	.000	17.294
477	.0000	.000	.000	.000	.000
478	.0000	.000	.000	.000	.000
479	-4.3838	81.393	.410	.000	17.309
480	.0000	.000	.000	.000	.000
481	.0000	.000	.000	.000	.000
482	.0000	.000	.000	.000	.000
483	.0000	.000	.000	.000	.000
484	.0000	.000	.000	.000	.000
485	.0000	100.000	.099	.000	19.745
486	-1.9280	72.603	-.455	.000	19.707
487	.0000	.000	.000	.000	.000
488	.0000	93.506	-.180	.000	19.639
489	.0000	.000	.000	.000	.000
490	.0000	.000	.000	.000	.000

	pig_code	ID_gender	Pair_ID	Cycle_total	P_or_V	onset_ref
491	5824	1	11	9.000	1	0
492	5824	1	11	9.000	0	0
493	5824	1	1	10.000	0	0
494	5824	1	1	10.000	0	0
495	5824	1	3	10.000	0	0
496	5824	1	3	10.000	0	0
497	5824	1	5	10.000	1	0
498	5824	1	6	10.000	2	0
499	5824	1	7	10.000	0	0
500	5824	1	8	10.000	2	0
501	5824	1	9	10.000	0	0
502	5824	1	9	10.000	0	0
503	5824	1	11	10.000	1	0
504	5824	1	11	10.000	0	0
505	5824	1	1	11.000	0	0
506	5824	1	1	11.000	0	0
507	5824	1	3	11.000	0	0
508	5824	1	3	11.000	0	0
509	5824	1	5	11.000	1	0
510	5824	1	6	11.000	2	0
511	5824	1	7	11.000	0	0
512	5824	1	8	11.000	2	0
513	5824	1	9	11.000	0	0
514	5824	1	9	11.000	0	0
515	5824	1	11	11.000	1	0
516	5824	1	11	11.000	0	0
517	5824	1	1	12.000	0	0
518	5824	1	1	12.000	0	0
519	5824	1	3	12.000	0	0
520	5824	1	3	12.000	0	0
521	?	?	?	?	?	?
522	?	?	?	?	?	?
523	?	?	?	?	?	?
524	?	?	?	?	?	?
525	?	?	?	?	?	?

Sono respiration_Raw data.sav

	resp_duration	insp_duration	exp_duration	base_value	Peak_or_Valley	Amplitude
491	2	1.049	.824	7.340	7.310	.030
492	0	.000	.000	.000	.000	.000
493	0	.000	.000	.000	.000	.000
494	0	.000	.000	.000	.000	.000
495	0	.000	.000	.000	.000	.000
496	0	.000	.000	.000	.000	.000
497	2	1.012	.929	-30.190	30.220	.030
498	2	.937	.614	33.090	-32.960	.130
499	0	.000	.000	.000	.000	.000
500	2	1.004	.727	33.320	-33.270	66.590
501	0	.000	.000	.000	.000	.000
502	0	.000	.000	.000	.000	.000
503	2	1.146	.532	7.340	7.300	.040
504	0	.000	.000	.000	.000	.000
505	0	.000	.000	.000	.000	.000
506	0	.000	.000	.000	.000	.000
507	0	.000	.000	.000	.000	.000
508	0	.000	.000	.000	.000	.000
509	2	1.102	.891	-30.190	30.220	.030
510	2	.555	1.371	33.110	-33.040	.070
511	0	.000	.000	.000	.000	.000
512	2	1.191	.735	33.320	-33.270	.050
513	0	.000	.000	.000	.000	.000
514	0	.000	.000	.000	.000	.000
515	2	1.236	.705	7.340	7.320	.020
516	0	.000	.000	.000	.000	.000
517	0	.000	.000	.000	.000	.000
518	0	.000	.000	.000	.000	.000
519	0	.000	.000	.000	.000	.000
520	0	.000	.000	.000	.000	.000
521	2	1.274	.869	-30.200	?	?
522	2	.622	1.364	33.130	?	?
523	0	.000	.000	.000	?	?
524	2	1.191	.787	33.320	?	?
525	0	.000	.000	.000	?	?

Sono respiration_Raw data.sav

	onset_percentage	duration_percentage	amplitude_percentage	P_V	onset
491	-9.5383	95.028	.410	.000	19.557
492	.0000	.000	.000	.000	.000
493	.0000	.000	.000	.000	.000
494	.0000	.000	.000	.000	.000
495	.0000	.000	.000	.000	.000
496	.0000	.000	.000	.000	.000
497	.0000	100.000	.099	.000	21.918
498	3.8640	79.907	-.394	.000	21.993
499	.0000	.000	.000	.000	.000
500	-3.5033	89.181	-2.150	.000	21.850
501	.0000	.000	.000	.000	.000
502	.0000	.000	.000	.000	.000
503	-.7728	86.450	.548	.000	21.903
504	.0000	.000	.000	.000	.000
505	.0000	.000	.000	.000	.000
506	.0000	.000	.000	.000	.000
507	.0000	.000	.000	.000	.000
508	.0000	.000	.000	.000	.000
509	.0000	100.000	.099	.000	24.165
510	-12.3934	96.638	-.212	.000	23.918
511	.0000	.000	.000	.000	.000
512	-9.3828	96.638	-.150	.000	23.978
513	.0000	.000	.000	.000	.000
514	.0000	.000	.000	.000	.000
515	-7.1249	97.391	.273	.000	24.023
516	.0000	.000	.000	.000	.000
517	.0000	.000	.000	.000	.000
518	.0000	.000	.000	.000	.000
519	.0000	.000	.000	.000	.000
520	.0000	.000	.000	.000	.000
521	.0000	100.000	.066	.000	26.331
522	-6.2996	92.674	-.394	.000	26.196
523	.0000	.000	.000	.000	.000
524	-2.4265	92.301	-.150	.000	26.279
525	.0000	.000	.000	.000	.000

	pig_code	ID_gender	Pair_ID	Cycle_total	P_or_V	onset_ref
526	5824	1	9	12.000	0	0
527	5824	1	11	12.000	1	0
528	5824	1	11	12.000	0	0
529	5824	1	1	13.000	0	0
530	5824	1	1	13.000	0	0
531	5824	1	3	13.000	0	0
532	5824	1	3	13.000	0	0
533	5824	1	5	13.000	1	0
534	5824	1	6	13.000	2	0
535	5824	1	7	13.000	0	0
536	5824	1	8	13.000	2	0
537	5824	1	9	13.000	0	0
538	5824	1	9	13.000	0	0
539	5824	1	11	13.000	1	0
540	5824	1	11	13.000	0	0
541	5824	1	1	14.000	0	0
542	5824	1	1	14.000	0	0
543	5824	1	3	14.000	0	0
544	5824	1	3	14.000	0	0
545	5824	1	5	14.000	1	0
546	5824	1	6	14.000	2	0
547	5824	1	7	14.000	0	0
548	5824	1	8	14.000	2	0
549	5824	1	9	14.000	0	0
550	5824	1	9	14.000	0	0
551	5824	1	11	14.000	1	0
552	5824	1	11	14.000	0	0
553	5824	1	1	15.000	0	0
554	5824	1	1	15.000	0	0
555	5824	1	3	15.000	0	0
556	5824	1	3	15.000	0	0
557	5824	1	5	15.000	1	0
558	5824	1	6	15.000	2	0
559	5824	1	7	15.000	0	0
560	5824	1	8	15.000	2	0

Sono respiration_Raw data.sav

	resp_duration	insp_duration	exp_duration	base_value	Peak_or_Valley	Amplitude
526	0	.000	.000	.000	.000	.000
527	2	1.206	.765	7.350	7.320	.030
528	0	.000	.000	.000	.000	.000
529	0	.000	.000	.000	.000	.000
530	0	.000	.000	.000	.000	.000
531	0	.000	.000	.000	.000	.000
532	0	.000	.000	.000	.000	.000
533	2	1.139	.832	-30.190	30.220	.030
534	1	.607	.809	33.110	-32.990	.120
535	0	.000	.000	.000	.000	.000
536	2	1.131	.705	33.320	-33.270	.050
537	0	.000	.000	.000	.000	.000
538	0	.000	.000	.000	.000	.000
539	2	1.087	.794	7.350	7.320	.030
540	0	.000	.000	.000	.000	.000
541	0	.000	.000	.000	.000	.000
542	0	.000	.000	.000	.000	.000
543	0	.000	.000	.000	.000	.000
544	0	.000	.000	.000	.000	.000
545	2	1.176	.990	-30.190	30.210	.020
546	1	.779	.660	33.110	-32.970	.140
547	0	.000	.000	.000	.000	.000
548	2	1.131	.840	33.330	-33.270	.060
549	0	.000	.000	.000	.000	.000
550	0	.000	.000	.000	.000	.000
551	2	1.176	.810	7.340	7.300	.040
552	0	.000	.000	.000	.000	.000
553	0	.000	.000	.000	.000	.000
554	0	.000	.000	.000	.000	.000
555	0	.000	.000	.000	.000	.000
556	0	.000	.000	.000	.000	.000
557	2	1.251	.856	-30.180	30.210	.030
558	2	.877	.719	33.090	-32.970	.120
559	0	.000	.000	.000	.000	.000
560	2	1.154	.832	33.330	-33.270	.060

Sono respiration_Raw data.sav

	onset_percentage	duration_percentage	amplitude_percentage	P_V	onset
526	.0000	.000	.000	.000	.000
527	-4.1997	91.974	.410	.000	26.241
528	.0000	.000	.000	.000	.000
529	.0000	.000	.000	.000	.000
530	.0000	.000	.000	.000	.000
531	.0000	.000	.000	.000	.000
532	.0000	.000	.000	.000	.000
533	.0000	100.000	.099	.000	28.684
534	-6.0883	71.842	-.364	.000	28.564
535	.0000	.000	.000	.000	.000
536	-4.9214	93.151	-.150	.000	28.587
537	.0000	.000	.000	.000	.000
538	.0000	.000	.000	.000	.000
539	-4.9214	95.434	.410	.000	28.587
540	.0000	.000	.000	.000	.000
541	.0000	.000	.000	.000	.000
542	.0000	.000	.000	.000	.000
543	.0000	.000	.000	.000	.000
544	.0000	.000	.000	.000	.000
545	.0000	100.000	.066	.000	30.887
546	-1.3850	66.436	-.425	.000	30.857
547	.0000	.000	.000	.000	.000
548	-1.3850	90.997	-.180	.000	30.857
549	.0000	.000	.000	.000	.000
550	.0000	.000	.000	.000	.000
551	-1.3850	91.690	.548	.000	30.857
552	.0000	.000	.000	.000	.000
553	.0000	.000	.000	.000	.000
554	.0000	.000	.000	.000	.000
555	.0000	.000	.000	.000	.000
556	.0000	.000	.000	.000	.000
557	.0000	100.000	.099	.000	33.165
558	-.7119	75.748	-.364	.000	33.150
559	.0000	.000	.000	.000	.000
560	-1.7561	94.257	-.180	.000	33.128

Sono respiration_Raw data.sav

	pig_code	ID_gender	Pair_ID	Cycle_total	P_or_V	onset_ref
561	5824	1	9	15.000	0	0
562	5824	1	9	15.000	0	0
563	5824	1	11	15.000	1	0
564	5824	1	11	15.000	0	0
565	6487	2	1	1.000	1	0
566	6487	2	1	1.000	1	0
567	6487	2	3	1.000	0	0
568	6487	2	3	1.000	0	0
569	6487	2	5	1.000	1	0
570	6487	2	6	1.000	0	0
571	6487	2	7	1.000	0	0
572	6487	2	8	1.000	0	0
573	6487	2	9	1.000	1	0
574	6487	2	9	1.000	1	0
575	6487	2	11	1.000	0	0
576	6487	2	11	1.000	0	0
577	6487	2	1	2.000	1	0
578	6487	2	1	2.000	1	0
579	6487	2	3	2.000	0	0
580	6487	2	3	2.000	0	0
581	6487	2	5	2.000	1	0
582	6487	2	6	2.000	0	0
583	6487	2	7	2.000	0	0
584	6487	2	8	2.000	0	0
585	6487	2	9	2.000	1	0
586	6487	2	9	2.000	1	0
587	6487	2	11	2.000	0	0
588	6487	2	11	2.000	0	0
589	6487	2	1	3.000	1	0
590	6487	2	1	3.000	1	0
591	6487	2	3	3.000	0	0
592	6487	2	3	3.000	0	0
593	6487	2	5	3.000	1	0
594	6487	2	6	3.000	0	0
595	6487	2	7	3.000	0	0

	resp_duration	insp_duration	exp_duration	base_value	Peak_or_Valley	Amplitude
561	0	.000	.000	.000	.000	.000
562	0	.000	.000	.000	.000	.000
563	2	.997	.861	7.340	7.300	.040
564	0	.000	.000	.000	.000	.000
565	3	.232	2.562	-6.330	6.540	.210
566	3	.322	2.825	-30.160	30.580	.420
567	0	.000	.000	.000	.000	.000
568	0	.000	.000	.000	.000	.000
569	3	.217	2.630	-29.430	29.490	.060
570	0	.000	.000	.000	.000	.000
571	0	.000	.000	.000	.000	.000
572	0	.000	.000	.000	.000	.000
573	3	.337	2.346	19.030	18.580	.450
574	3	.322	2.735	-31.400	31.840	.440
575	0	.000	.000	.000	.000	.000
576	0	.000	.000	.000	.000	.000
577	3	.217	2.383	-6.330	6.530	.200
578	3	.330	2.248	-30.160	30.560	.400
579	0	.000	.000	.000	.000	.000
580	0	.000	.000	.000	.000	.000
581	3	.277	2.398	-29.430	29.490	.060
582	0	.000	.000	.000	.000	.000
583	0	.000	.000	.000	.000	.000
584	0	.000	.000	.000	.000	.000
585	2	.337	1.971	19.040	18.590	.450
586	3	.300	2.375	-31.400	31.820	.420
587	0	.000	.000	.000	.000	.000
588	0	.000	.000	.000	.000	.000
589	3	.277	2.713	-6.330	6.520	.190
590	3	.337	2.833	-30.150	30.560	.410
591	0	.000	.000	.000	.000	.000
592	0	.000	.000	.000	.000	.000
593	3	.270	2.794	-29.420	29.490	.070
594	0	.000	.000	.000	.000	.000
595	0	.000	.000	.000	.000	.000

	onset_percentage	duration_percentage	amplitude_percentage	P_V	onset
561	.0000	.000	.000	.000	.000
562	.0000	.000	.000	.000	.000
563	2.8477	88.182	.548	.000	33.225
564	.0000	.000	.000	.000	.000
565	-1.0537	98.138	3.211	.000	4.279
566	-1.3347	110.537	1.373	.000	4.271
567	.0000	.000	.000	.000	.000
568	.0000	.000	.000	.000	.000
569	.0000	100.000	.203	.000	4.309
570	.0000	.000	.000	.000	.000
571	.0000	.000	.000	.000	.000
572	.0000	.000	.000	.000	.000
573	-2.6344	94.240	2.422	.000	4.234
574	-1.8616	107.376	1.382	.000	4.256
575	.0000	.000	.000	.000	.000
576	.0000	.000	.000	.000	.000
577	.8598	78.339	3.063	.000	7.958
578	.2991	119.134	1.309	.000	7.943
579	.0000	.000	.000	.000	.000
580	.0000	.000	.000	.000	.000
581	.0000	100.000	.203	.000	7.935
582	.0000	.000	.000	.000	.000
583	.0000	.000	.000	.000	.000
584	.0000	.000	.000	.000	.000
585	-12.5981	86.280	2.421	.000	7.598
586	.2991	100.000	1.320	.000	7.943
587	.0000	.000	.000	.000	.000
588	.0000	.000	.000	.000	.000
589	-1.4360	97.585	2.914	.000	11.293
590	-1.2076	103.460	1.342	.000	11.300
591	.0000	.000	.000	.000	.000
592	.0000	.000	.000	.000	.000
593	.0000	100.000	.237	.000	11.337
594	.0000	.000	.000	.000	.000
595	.0000	.000	.000	.000	.000

Sono respiration_Raw data.sav

	pig_code	ID_gender	Pair_ID	Cycle_total	P_or_V	onset_ref
596	6487	2	8	3.000	0	0
597	6487	2	9	3.000	1	0
598	6487	2	9	3.000	1	0
599	6487	2	11	3.000	0	0
600	6487	2	11	3.000	0	0
601	6487	2	1	4.000	1	0
602	6487	2	1	4.000	1	0
603	6487	2	3	4.000	0	0
604	6487	2	3	4.000	0	0
605	6487	2	5	4.000	1	0
606	6487	2	6	4.000	0	0
607	6487	2	7	4.000	0	0
608	6487	2	8	4.000	0	0
609	6487	2	9	4.000	1	0
610	6487	2	9	4.000	1	0
611	6487	2	11	4.000	0	0
612	6487	2	11	4.000	0	0
613	6487	2	1	5.000	1	0
614	6487	2	1	5.000	1	0
615	6487	2	3	5.000	0	0
616	6487	2	3	5.000	0	0
617	6487	2	5	5.000	1	0
618	6487	2	6	5.000	0	0
619	6487	2	7	5.000	0	0
620	6487	2	8	5.000	0	0
621	?	?	?	?	?	?
622	?	?	?	?	?	?
623	?	?	?	?	?	?
624	?	?	?	?	?	?
625	?	?	?	?	?	?
626	?	?	?	?	?	?
627	?	?	?	?	?	?
628	?	?	?	?	?	?
629	?	?	?	?	?	?
630	?	?	?	?	?	?

Sono respiration_Raw data.sav

	resp_duration	insp_duration	exp_duration	base_value	Peak_or_Valley	Amplitude
596	0	.000	.000	.000	.000	.000
597	3	.375	2.442	19.030	18.590	.450
598	3	.300	2.840	-31.400	31.830	.430
599	0	.000	.000	.000	.000	.000
600	0	.000	.000	.000	.000	.000
601	3	.300	2.615	-6.330	6.520	.190
602	3	.315	2.547	-30.150	30.550	.400
603	0	.000	.000	.000	.000	.000
604	0	.000	.000	.000	.000	.000
605	3	.277	2.668	-29.430	29.490	.060
606	0	.000	.000	.000	.000	.000
607	0	.000	.000	.000	.000	.000
608	0	.000	.000	.000	.000	.000
609	3	.390	2.315	19.030	18.590	.440
610	3	.337	2.713	-31.400	31.810	.410
611	0	.000	.000	.000	.000	.000
612	0	.000	.000	.000	.000	.000
613	3	.277	2.488	-6.310	6.520	.210
614	3	.315	2.517	-30.150	30.560	.410
615	0	.000	.000	.000	.000	.000
616	0	.000	.000	.000	.000	.000
617	3	.202	2.533	-29.430	29.480	29.480
618	0	.000	.000	.000	.000	.000
619	0	.000	.000	.000	.000	.000
620	0	.000	.000	.000	.000	.000
621	2	.330	2.128	19.030	?	?
622	3	.367	2.383	-31.390	?	?
623	0	.000	.000	.000	?	?
624	0	.000	.000	.000	?	?
625	3	.277	2.728	-6.310	?	?
626	3	.322	2.660	-30.140	?	?
627	0	.000	.000	.000	?	?
628	0	.000	.000	.000	?	?
629	3	.255	2.772	-29.420	?	?
630	0	.000	.000	.000	?	?

	onset_percentage	duration_percentage	amplitude_percentage	P_V	onset
596	.0000	.000	.000	.000	.000
597	-1.7298	91.939	2.422	.000	11.284
598	-.7180	102.480	1.351	.000	11.315
599	.0000	.000	.000	.000	.000
600	.0000	.000	.000	.000	.000
601	.3396	101.852	2.914	.000	15.159
602	.3396	97.182	1.309	.000	15.159
603	.0000	.000	.000	.000	.000
604	.0000	.000	.000	.000	.000
605	.0000	100.000	.203	.000	15.159
606	.0000	.000	.000	.000	.000
607	.0000	.000	.000	.000	.000
608	.0000	.000	.000	.000	.000
609	-1.0187	91.851	2.367	.000	15.129
610	.3396	103.565	1.289	.000	15.159
611	.0000	.000	.000	.000	.000
612	.0000	.000	.000	.000	.000
613	-.5484	101.097	3.221	.000	18.808
614	.3656	103.547	1.342	.000	18.823
615	.0000	.000	.000	.000	.000
616	.0000	.000	.000	.000	.000
617	.0000	100.000	1.000	.000	18.823
618	.0000	.000	.000	.000	.000
619	.0000	.000	.000	.000	.000
620	.0000	.000	.000	.000	.000
621	-1.0969	89.872	2.422	.000	18.793
622	.2925	100.548	1.382	.000	18.831
623	.0000	.000	.000	.000	.000
624	.0000	.000	.000	.000	.000
625	.3304	99.273	3.369	.000	22.322
626	.3304	98.513	1.374	.000	22.322
627	.0000	.000	.000	.000	.000
628	.0000	.000	.000	.000	.000
629	.0000	100.000	.237	.000	22.322
630	.0000	.000	.000	.000	.000

Sono respiration_Raw data.sav

	pig_code	ID_gender	Pair_ID	Cycle_total	P_or_V	onset_ref
631	6487	2	7	6.000	0	0
632	6487	2	8	6.000	0	0
633	6487	2	9	6.000	1	0
634	6487	2	9	6.000	1	0
635	6487	2	11	6.000	0	0
636	6487	2	11	6.000	0	0
637	6487	2	1	7.000	1	0
638	6487	2	1	7.000	1	0
639	6487	2	3	7.000	0	0
640	6487	2	3	7.000	0	0
641	6487	2	5	7.000	1	0
642	6487	2	6	7.000	0	0
643	6487	2	7	7.000	0	0
644	6487	2	8	7.000	0	0
645	6487	2	9	7.000	1	0
646	6487	2	9	7.000	1	0
647	6487	2	11	7.000	0	0
648	6487	2	11	7.000	0	0
649	6487	2	1	8.000	1	0
650	6487	2	1	8.000	1	0
651	6487	2	3	8.000	0	0
652	6487	2	3	8.000	0	0
653	6487	2	5	8.000	1	0
654	6487	2	6	8.000	0	0
655	6487	2	7	8.000	0	0
656	6487	2	8	8.000	0	0
657	6487	2	9	8.000	1	0
658	6487	2	9	8.000	1	0
659	6487	2	11	8.000	0	0
660	6487	2	11	8.000	0	0
661	?	?	?	?	?	?
662	?	?	?	?	?	?
663	?	?	?	?	?	?
664	?	?	?	?	?	?
665	?	?	?	?	?	?

	resp_duration	insp_duration	exp_duration	base_value	Peak_or_Valley	Amplitude
631	0	.000	.000	.000	.000	.000
632	0	.000	.000	.000	.000	.000
633	3	.345	2.390	19.030	18.590	.440
634	3	.330	2.502	-31.390	31.820	.430
635	0	.000	.000	.000	.000	.000
636	0	.000	.000	.000	.000	.000
637	3	.285	2.817	-6.330	6.530	.200
638	3	.330	2.750	-30.150	30.570	.420
639	0	.000	.000	.000	.000	.000
640	0	.000	.000	.000	.000	.000
641	3	.254	2.886	-29.440	29.490	.050
642	0	.000	.000	.000	.000	.000
643	0	.000	.000	.000	.000	.000
644	0	.000	.000	.000	.000	.000
645	3	.382	2.428	19.030	18.570	.460
646	3	.315	2.577	-31.400	31.830	.430
647	0	.000	.000	.000	.000	.000
648	0	.000	.000	.000	.000	.000
649	3	.352	2.151	-6.320	6.550	.230
650	3	.315	2.240	-30.150	30.560	.410
651	0	.000	.000	.000	.000	.000
652	0	.000	.000	.000	.000	.000
653	3	.262	2.398	-29.430	29.490	.060
654	0	.000	.000	.000	.000	.000
655	0	.000	.000	.000	.000	.000
656	0	.000	.000	.000	.000	.000
657	2	.322	2.008	19.040	18.650	.390
658	2	.315	2.113	-31.400	31.830	.430
659	0	.000	.000	.000	.000	.000
660	0	.000	.000	.000	.000	.000
661	3	.367	2.427	-6.340	?	?
662	3	.367	2.705	-30.140	?	?
663	0	.000	.000	.000	?	?
664	0	.000	.000	.000	?	?
665	3	.300	2.742	-29.420	?	?

	onset_percentage	duration_percentage	amplitude_percentage	P_V	onset
631	.0000	.000	.000	.000	.000
632	.0000	.000	.000	.000	.000
633	-.7268	90.353	2.367	.000	22.300
634	.7598	93.558	1.351	.000	22.345
635	.0000	.000	.000	.000	.000
636	.0000	.000	.000	.000	.000
637	-1.6561	98.790	3.063	.000	26.062
638	-1.1783	98.089	1.374	.000	26.077
639	.0000	.000	.000	.000	.000
640	.0000	.000	.000	.000	.000
641	.0000	100.000	.170	.000	26.114
642	.0000	.000	.000	.000	.000
643	.0000	.000	.000	.000	.000
644	.0000	.000	.000	.000	.000
645	-1.1783	89.490	2.477	.000	26.077
646	-1.1783	92.102	1.351	.000	26.077
647	.0000	.000	.000	.000	.000
648	.0000	.000	.000	.000	.000
649	1.1278	94.098	3.511	.000	29.958
650	1.4286	96.053	1.342	.000	29.966
651	.0000	.000	.000	.000	.000
652	.0000	.000	.000	.000	.000
653	.0000	100.000	.203	.000	29.928
654	.0000	.000	.000	.000	.000
655	.0000	.000	.000	.000	.000
656	.0000	.000	.000	.000	.000
657	.5639	87.594	2.091	.000	29.943
658	1.4286	91.278	1.351	.000	29.966
659	.0000	.000	.000	.000	.000
660	.0000	.000	.000	.000	.000
661	.2630	91.847	2.910	.000	33.330
662	.3287	100.986	1.374	.000	33.322
663	.0000	.000	.000	.000	.000
664	.0000	.000	.000	.000	.000
665	.0000	100.000	.204	.000	33.322

Sono respiration_Raw data.sav

	pig_code	ID_gender	Pair_ID	Cycle_total	P_or_V	onset_ref
666	6487	2	6	9.000	0	0
667	6487	2	7	9.000	0	0
668	6487	2	8	9.000	0	0
669	6487	2	9	9.000	1	0
670	6487	2	9	9.000	1	0
671	6487	2	11	9.000	0	0
672	6487	2	11	9.000	0	0
673	6487	2	1	10.000	1	0
674	6487	2	1	10.000	1	0
675	6487	2	3	10.000	0	0
676	6487	2	3	10.000	0	0
677	6487	2	5	10.000	1	0
678	6487	2	6	10.000	0	0
679	6487	2	7	10.000	0	0
680	6487	2	8	10.000	0	0
681	6487	2	9	10.000	1	0
682	6487	2	9	10.000	1	0
683	6487	2	11	10.000	0	0
684	6487	2	11	10.000	0	0
685	6487	2	1	11.000	1	0
686	6487	2	1	11.000	1	0
687	6487	2	3	11.000	0	0
688	6487	2	3	11.000	0	0
689	6487	2	5	11.000	1	0
690	6487	2	6	11.000	0	0
691	6487	2	7	11.000	0	0
692	6487	2	8	11.000	0	0
693	6487	2	9	11.000	1	0
694	6487	2	9	11.000	1	0
695	6487	2	11	11.000	0	0
696	6487	2	11	11.000	0	0
697	6487	2	1	12.000	1	0
698	6487	2	1	12.000	1	0
699	6487	2	3	12.000	0	0
700	6487	2	3	12.000	0	0

	resp_duration	insp_duration	exp_duration	base_value	Peak_or_Valley	Amplitude
666	0	.000	.000	.000	.000	.000
667	0	.000	.000	.000	.000	.000
668	0	.000	.000	.000	.000	.000
669	3	.375	2.172	18.990	18.540	.450
670	3	.322	2.488	-31.390	31.820	.430
671	0	.000	.000	.000	.000	.000
672	0	.000	.000	.000	.000	.000
673	3	.367	2.256	-6.320	6.540	.220
674	3	.367	2.495	-30.130	30.540	.410
675	0	.000	.000	.000	.000	.000
676	0	.000	.000	.000	.000	.000
677	3	.330	2.480	-29.430	29.500	.070
678	0	.000	.000	.000	.000	.000
679	0	.000	.000	.000	.000	.000
680	0	.000	.000	.000	.000	.000
681	3	.360	2.225	18.990	18.530	.460
682	3	.292	2.466	-31.390	31.820	.430
683	0	.000	.000	.000	.000	.000
684	0	.000	.000	.000	.000	.000
685	3	.315	2.495	-6.330	6.540	.210
686	3	.352	2.533	-30.140	30.550	.410
687	0	.000	.000	.000	.000	.000
688	0	.000	.000	.000	.000	.000
689	3	.307	2.518	-29.440	29.490	.050
690	0	.000	.000	.000	.000	.000
691	0	.000	.000	.000	.000	.000
692	0	.000	.000	.000	.000	.000
693	3	.360	2.338	18.980	18.530	.450
694	3	.322	2.428	-31.390	31.820	.430
695	0	.000	.000	.000	.000	.000
696	0	.000	.000	.000	.000	.000
697	3	.337	2.413	-6.310	6.520	.210
698	3	.367	2.226	-30.140	30.550	.410
699	0	.000	.000	.000	.000	.000
700	0	.000	.000	.000	.000	.000

Sono respiration_Raw data.sav

	onset_percentage	duration_percentage	amplitude_percentage	P_V	onset
666	.0000	.000	.000	.000	.000
667	.0000	.000	.000	.000	.000
668	.0000	.000	.000	.000	.000
669	-.9533	83.728	2.427	.000	33.293
670	-.4931	92.373	1.351	.000	33.307
671	.0000	.000	.000	.000	.000
672	.0000	.000	.000	.000	.000
673	-.5338	93.345	3.364	.000	36.934
674	-.2491	101.851	1.343	.000	36.942
675	.0000	.000	.000	.000	.000
676	.0000	.000	.000	.000	.000
677	.0000	100.000	.237	.000	36.949
678	.0000	.000	.000	.000	.000
679	.0000	.000	.000	.000	.000
680	.0000	.000	.000	.000	.000
681	-1.3167	91.993	2.482	.000	36.912
682	-1.0676	98.149	1.351	.000	36.919
683	.0000	.000	.000	.000	.000
684	.0000	.000	.000	.000	.000
685	-.8142	99.469	3.211	.000	40.568
686	-.5310	102.124	1.342	.000	40.576
687	.0000	.000	.000	.000	.000
688	.0000	.000	.000	.000	.000
689	.0000	100.000	.170	.000	40.591
690	.0000	.000	.000	.000	.000
691	.0000	.000	.000	.000	.000
692	.0000	.000	.000	.000	.000
693	-1.5929	95.504	2.428	.000	40.546
694	-.5310	97.345	1.351	.000	40.576
695	.0000	.000	.000	.000	.000
696	.0000	.000	.000	.000	.000
697	-.5367	98.390	3.221	.000	44.173
698	.3578	92.773	1.342	.000	44.188
699	.0000	.000	.000	.000	.000
700	.0000	.000	.000	.000	.000

Sono respiration_Raw data.sav

	pig_code	ID_gender	Pair_ID	Cycle_total	P_or_V	onset_ref
701	6487	2	5	12.000	1	0
702	6487	2	6	12.000	0	0
703	6487	2	7	12.000	0	0
704	6487	2	8	12.000	0	0
705	6487	2	9	12.000	1	0
706	6487	2	9	12.000	1	0
707	6487	2	11	12.000	0	0
708	6487	2	11	12.000	0	0
709	6487	2	1	13.000	1	0
710	6487	2	1	13.000	1	-3
711	6487	2	3	13.000	0	0
712	6487	2	3	13.000	0	0
713	6487	2	5	13.000	1	0
714	6487	2	6	13.000	0	0
715	6487	2	7	13.000	0	0
716	6487	2	8	13.000	0	0
717	6487	2	9	13.000	1	-3
718	6487	2	9	13.000	1	-3
719	6487	2	11	13.000	0	0
720	6487	2	11	13.000	0	0
721	6487	2	1	14.000	1	0
722	6487	2	1	14.000	1	0
723	6487	2	3	14.000	0	0
724	6487	2	3	14.000	0	0
725	6487	2	5	14.000	1	0
726	6487	2	6	14.000	0	0
727	6487	2	7	14.000	0	0
728	6487	2	8	14.000	0	0
729	6487	2	9	14.000	1	0
730	6487	2	9	14.000	1	0
731	6487	2	11	14.000	0	0
732	6487	2	11	14.000	0	0
733	6487	2	1	15.000	1	0
734	6487	2	1	15.000	1	0
735	6487	2	3	15.000	0	0

	resp_duration	insp_duration	exp_duration	base_value	Peak_or_Valley	Amplitude
701	3	.315	2.480	-29.420	29.490	.070
702	0	.000	.000	.000	.000	.000
703	0	.000	.000	.000	.000	.000
704	0	.000	.000	.000	.000	.000
705	2	.375	2.105	18.980	18.530	.450
706	3	.300	2.240	-31.690	31.820	.130
707	0	.000	.000	.000	.000	.000
708	0	.000	.000	.000	.000	.000
709	3	.285	2.645	-6.340	6.530	.190
710	3	.352	2.570	-30.130	30.550	.420
711	0	.000	.000	.000	.000	.000
712	0	.000	.000	.000	.000	.000
713	3	.254	2.720	-29.450	29.480	.030
714	0	.000	.000	.000	.000	.000
715	0	.000	.000	.000	.000	.000
716	0	.000	.000	.000	.000	.000
717	3	.367	2.301	18.990	18.520	.470
718	3	.300	2.502	-31.390	31.820	.430
719	0	.000	.000	.000	.000	.000
720	0	.000	.000	.000	.000	.000
721	3	.255	2.480	-6.330	6.540	.210
722	3	.375	2.465	-30.140	30.550	.410
723	0	.000	.000	.000	.000	.000
724	0	.000	.000	.000	.000	.000
725	3	.240	2.682	-29.440	29.490	.050
726	0	.000	.000	.000	.000	.000
727	0	.000	.000	.000	.000	.000
728	0	.000	.000	.000	.000	.000
729	3	.352	2.188	18.990	18.530	.460
730	3	.330	2.435	-31.380	31.820	.440
731	0	.000	.000	.000	.000	.000
732	0	.000	.000	.000	.000	.000
733	3	.255	2.503	-6.340	6.540	.200
734	3	.330	2.390	-30.130	30.550	.420
735	0	.000	.000	.000	.000	.000

Sono respiration_Raw data.sav

	onset_percentage	duration_percentage	amplitude_percentage	P_V	onset
701	.0000	100.000	.237	.000	44.188
702	.0000	.000	.000	.000	.000
703	.0000	.000	.000	.000	.000
704	.0000	.000	.000	.000	.000
705	-.5367	88.730	2.428	.000	44.173
706	1.0733	90.877	.409	.000	44.218
707	.0000	.000	.000	.000	.000
708	.0000	.000	.000	.000	.000
709	.3362	98.521	2.910	.000	47.732
710	-9.7646	98.252	1.375	.000	47.732
711	.0000	.000	.000	.000	.000
712	.0000	.000	.000	.000	.000
713	.0000	100.000	.102	.000	50.699
714	.0000	.000	.000	.000	.000
715	.0000	.000	.000	.000	.000
716	.0000	.000	.000	.000	.000
717	-10.2690	89.711	2.538	.000	47.717
718	-10.5044	94.217	1.351	.000	47.710
719	.0000	.000	.000	.000	.000
720	.0000	.000	.000	.000	.000
721	.7529	93.600	3.211	.000	51.501
722	.5133	97.194	1.342	.000	51.494
723	.0000	.000	.000	.000	.000
724	.0000	.000	.000	.000	.000
725	.0000	100.000	.170	.000	51.479
726	.0000	.000	.000	.000	.000
727	.0000	.000	.000	.000	.000
728	.0000	.000	.000	.000	.000
729	-.5133	86.927	2.482	.000	51.464
730	-.2738	94.627	1.383	.000	51.471
731	.0000	.000	.000	.000	.000
732	.0000	.000	.000	.000	.000
733	-.2847	98.149	3.058	.000	55.180
734	-.8185	96.797	1.375	.000	55.165
735	.0000	.000	.000	.000	.000

Sono respiration_Raw data.sav

	pig_code	ID_gender	Pair_ID	Cycle_total	P_or_V	onset_ref
736	6487	2	3	15.000	0	0
737	6487	2	5	15.000	1	0
738	6487	2	6	15.000	0	0
739	6487	2	7	15.000	0	0
740	6487	2	8	15.000	0	0
741	6487	2	9	15.000	1	0
742	6487	2	9	15.000	1	0
743	6487	2	11	15.000	0	0
744	6487	2	11	15.000	0	0
745	6238	1	1	1.000	0	0
746	6238	1	1	1.000	1	0
747	6238	1	3	1.000	0	0
748	6238	1	3	1.000	0	0
749	6238	1	5	1.000	1	0
750	6238	1	6	1.000	0	0
751	6238	1	7	1.000	0	0
752	6238	1	8	1.000	2	0
753	6238	1	9	1.000	0	0
754	6238	1	9	1.000	0	0
755	6238	1	11	1.000	0	0
756	6238	1	11	1.000	1	0
757	6238	1	1	2.000	0	0
758	6238	1	1	2.000	1	0
759	6238	1	3	2.000	0	0
760	6238	1	3	2.000	0	0
761	?	?	?	?	?	?
762	?	?	?	?	?	?
763	?	?	?	?	?	?
764	?	?	?	?	?	?
765	?	?	?	?	?	?
766	?	?	?	?	?	?
767	?	?	?	?	?	?
768	?	?	?	?	?	?
769	?	?	?	?	?	?
770	?	?	?	?	?	?

	resp_duration	insp_duration	exp_duration	base_value	Peak_or_Valley	Amplitude
736	0	.000	.000	.000	.000	.000
737	3	.255	2.555	-29.440	29.500	.060
738	0	.000	.000	.000	.000	.000
739	0	.000	.000	.000	.000	.000
740	0	.000	.000	.000	.000	.000
741	2	.345	2.135	18.990	18.540	.450
742	3	.292	2.353	-31.380	-31.820	-63.200
743	0	.000	.000	.000	.000	.000
744	0	.000	.000	.000	.000	.000
745	0	.000	.000	.000	.000	.000
746	2	1.131	.757	-11.790	11.810	.020
747	0	.000	.000	.000	.000	.000
748	0	.000	.000	.000	.000	.000
749	2	1.109	.712	-26.250	26.280	.030
750	0	.000	.000	.000	.000	.000
751	0	.000	.000	.000	.000	.000
752	2	1.214	.412	13.530	-13.480	.050
753	0	.000	.000	.000	.000	.000
754	0	.000	.000	.000	.000	.000
755	0	.000	.000	.000	.000	.000
756	2	1.199	.502	15.970	15.920	.050
757	0	.000	.000	.000	.000	.000
758	2	.952	.704	-11.810	11.820	.010
759	0	.000	.000	.000	.000	.000
760	0	.000	.000	.000	.000	.000
761	2	.944	.787	-26.250	?	?
762	0	.000	.000	.000	?	?
763	0	.000	.000	.000	?	?
764	2	.974	.607	13.520	?	?
765	0	.000	.000	.000	?	?
766	0	.000	.000	.000	?	?
767	0	.000	.000	.000	?	?
768	2	1.154	.457	15.960	?	?
769	0	.000	.000	.000	?	?
770	2	.944	.817	-11.790	?	?

	onset_percentage	duration_percentage	amplitude_percentage	P_V	onset
736	.0000	.000	.000	.000	.000
737	.0000	100.000	.203	.000	55.188
738	.0000	.000	.000	.000	.000
739	.0000	.000	.000	.000	.000
740	.0000	.000	.000	.000	.000
741	-2.1352	88.256	2.427	.000	55.128
742	-1.6014	94.128	-1.986	.000	55.143
743	.0000	.000	.000	.000	.000
744	.0000	.000	.000	.000	.000
745	.0000	.000	.000	.000	.000
746	-3.6793	103.679	.169	.000	1.716
747	.0000	.000	.000	.000	.000
748	.0000	.000	.000	.000	.000
749	.0000	100.000	.114	.000	1.783
750	.0000	.000	.000	.000	.000
751	.0000	.000	.000	.000	.000
752	-.3844	89.292	-.371	.000	1.776
753	.0000	.000	.000	.000	.000
754	.0000	.000	.000	.000	.000
755	.0000	.000	.000	.000	.000
756	-2.0319	93.410	.314	.000	1.746
757	.0000	.000	.000	.000	.000
758	4.3328	95.667	.085	.000	3.784
759	.0000	.000	.000	.000	.000
760	.0000	.000	.000	.000	.000
761	.0000	100.000	.114	.000	3.709
762	.0000	.000	.000	.000	.000
763	.0000	.000	.000	.000	.000
764	-1.2709	91.334	-1.046	.000	3.687
765	.0000	.000	.000	.000	.000
766	.0000	.000	.000	.000	.000
767	.0000	.000	.000	.000	.000
768	-2.1375	93.068	.314	.000	3.672
769	.0000	.000	.000	.000	.000
770	1.6474	96.705	.254	.000	5.627

	pig_code	ID_gender	Pair_ID	Cycle_total	P_or_V	onset_ref
771	6238	0	3	3.000	0	0
772	6238	0	3	3.000	0	0
773	6238	0	5	3.000	1	0
774	6238	0	6	3.000	0	0
775	6238	0	7	3.000	0	0
776	6238	0	8	3.000	2	6
777	6238	0	9	3.000	0	0
778	6238	0	9	3.000	0	0
779	6238	0	11	3.000	0	0
780	6238	0	11	3.000	1	0
781	6238	0	1	4.000	0	0
782	6238	0	1	4.000	1	0
783	6238	0	3	4.000	0	0
784	6238	0	3	4.000	0	0
785	6238	0	5	4.000	1	0
786	6238	0	6	4.000	0	0
787	6238	0	7	4.000	0	0
788	6238	0	8	4.000	2	0
789	6238	0	9	4.000	0	0
790	6238	0	9	4.000	0	0
791	6238	0	11	4.000	0	0
792	6238	0	11	4.000	1	0
793	6238	0	1	5.000	0	0
794	6238	0	1	5.000	1	0
795	6238	0	3	5.000	0	0
796	6238	0	3	5.000	0	0
797	6238	0	5	5.000	1	0
798	6238	0	6	5.000	0	0
799	6238	0	7	5.000	0	0
800	6238	0	8	5.000	2	0
801	?	?	?	?	?	?
802	?	?	?	?	?	?
803	?	?	?	?	?	?
804	?	?	?	?	?	?
805	?	?	?	?	?	?

Sono respiration_Raw data.sav

	resp_duration	insp_duration	exp_duration	base_value	Peak_or_Valley	Amplitude
771	0	.000	.000	.000	.000	.000
772	0	.000	.000	.000	.000	.000
773	2	1.109	.712	-26.250	26.280	.030
774	0	.000	.000	.000	.000	.000
775	0	.000	.000	.000	.000	.000
776	2	1.109	.555	13.520	-13.480	.040
777	0	.000	.000	.000	.000	.000
778	0	.000	.000	.000	.000	.000
779	0	.000	.000	.000	.000	.000
780	2	1.244	.479	15.970	15.900	.070
781	0	.000	.000	.000	.000	.000
782	2	1.049	.734	-11.820	11.830	.010
783	0	.000	.000	.000	.000	.000
784	0	.000	.000	.000	.000	.000
785	2	.982	.764	-26.250	26.280	.030
786	0	.000	.000	.000	.000	.000
787	0	.000	.000	.000	.000	.000
788	2	1.116	.458	13.520	-13.480	.040
789	0	.000	.000	.000	.000	.000
790	0	.000	.000	.000	.000	.000
791	0	.000	.000	.000	.000	.000
792	2	1.214	.509	15.970	15.900	.070
793	0	.000	.000	.000	.000	.000
794	2	.907	.682	-11.790	11.830	.040
795	0	.000	.000	.000	.000	.000
796	0	.000	.000	.000	.000	.000
797	2	.944	.877	-26.250	26.280	.030
798	0	.000	.000	.000	.000	.000
799	0	.000	.000	.000	.000	.000
800	2	1.049	.510	13.530	-13.490	.040
801	0	.000	.000	.000	?	?
802	0	.000	.000	.000	?	?
803	0	.000	.000	.000	?	?
804	2	1.079	.480	15.970	?	?
805	0	.000	.000	.000	?	?

Sono respiration_Raw data.sav

	onset_percentage	duration_percentage	amplitude_percentage	P_V	onset
771	.0000	.000	.000	.000	.000
772	.0000	.000	.000	.000	.000
773	.0000	100.000	.114	.000	5.597
774	.0000	.000	.000	.000	.000
775	.0000	.000	.000	.000	.000
776	30.6332	91.378	-.297	.000	5.612
777	.0000	.000	.000	.000	.000
778	.0000	.000	.000	.000	.000
779	.0000	.000	.000	.000	.000
780	-1.6474	94.618	.440	.000	5.567
781	.0000	.000	.000	.000	.000
782	-.8591	102.119	.085	.000	7.598
783	.0000	.000	.000	.000	.000
784	.0000	.000	.000	.000	.000
785	.0000	100.000	.114	.000	7.613
786	.0000	.000	.000	.000	.000
787	.0000	.000	.000	.000	.000
788	3.0355	90.149	-.297	.000	7.666
789	.0000	.000	.000	.000	.000
790	.0000	.000	.000	.000	.000
791	.0000	.000	.000	.000	.000
792	-3.8373	98.683	.440	.000	7.546
793	.0000	.000	.000	.000	.000
794	9.8847	96.081	.338	.000	9.704
795	.0000	.000	.000	.000	.000
796	.0000	.000	.000	.000	.000
797	.0000	100.000	.114	.000	9.524
798	.0000	.000	.000	.000	.000
799	.0000	.000	.000	.000	.000
800	2.0319	85.612	-.297	.000	9.561
801	.0000	.000	.000	.000	.000
802	.0000	.000	.000	.000	.000
803	.0000	.000	.000	.000	.000
804	.8237	85.612	.440	.000	9.539
805	.0000	.000	.000	.000	.000

Sono respiration_Raw data.sav

	pig_code	ID_gender	Pair_ID	Cycle_total	P_or_V	onset_ref
806	6238	0	1	6.000	1	0
807	6238	0	3	6.000	0	0
808	6238	0	3	6.000	0	0
809	6238	0	5	6.000	1	0
810	6238	0	6	6.000	0	0
811	6238	0	7	6.000	0	0
812	6238	0	8	6.000	2	0
813	6238	0	9	6.000	0	0
814	6238	0	9	6.000	0	0
815	6238	0	11	6.000	0	0
816	6238	0	11	6.000	1	0
817	6238	0	1	7.000	0	0
818	6238	0	1	7.000	1	0
819	6238	0	3	7.000	0	0
820	6238	0	3	7.000	0	0
821	6238	0	5	7.000	1	0
822	6238	0	6	7.000	0	0
823	6238	0	7	7.000	0	0
824	6238	0	8	7.000	2	0
825	6238	0	9	7.000	0	0
826	6238	0	9	7.000	0	0
827	6238	0	11	7.000	0	0
828	6238	0	11	7.000	1	0
829	6238	0	1	8.000	0	0
830	6238	0	1	8.000	1	0
831	6238	0	3	8.000	0	0
832	6238	0	3	8.000	0	0
833	6238	0	5	8.000	1	0
834	6238	0	6	8.000	0	0
835	6238	0	7	8.000	0	0
836	6238	0	8	8.000	2	0
837	6238	0	9	8.000	0	0
838	6238	0	9	8.000	0	0
839	6238	0	11	8.000	0	0
840	6238	0	11	8.000	1	0

Sono respiration_Raw data.sav

	resp_duration	insp_duration	exp_duration	base_value	Peak_or_Valley	Amplitude
806	2	1.027	.764	-11.790	11.810	.020
807	0	.000	.000	.000	.000	.000
808	0	.000	.000	.000	.000	.000
809	2	1.004	.727	-26.250	26.270	.020
810	0	.000	.000	.000	.000	.000
811	0	.000	.000	.000	.000	.000
812	2	1.109	.614	13.530	-13.480	.050
813	0	.000	.000	.000	.000	.000
814	0	.000	.000	.000	.000	.000
815	0	.000	.000	.000	.000	.000
816	2	1.184	.539	15.960	15.900	.060
817	0	.000	.000	.000	.000	.000
818	2	1.169	.689	-11.790	11.830	.040
819	0	.000	.000	.000	.000	.000
820	0	.000	.000	.000	.000	.000
821	2	1.064	.854	-26.260	26.280	.020
822	0	.000	.000	.000	.000	.000
823	0	.000	.000	.000	.000	.000
824	2	1.176	.473	13.520	-13.490	.030
825	0	.000	.000	.000	.000	.000
826	0	.000	.000	.000	.000	.000
827	0	.000	.000	.000	.000	.000
828	2	1.236	.562	15.970	15.900	.070
829	0	.000	.000	.000	.000	.000
830	2	1.057	.711	-11.800	11.810	.010
831	0	.000	.000	.000	.000	.000
832	0	.000	.000	.000	.000	.000
833	2	.884	.817	-26.250	26.280	26.280
834	0	.000	.000	.000	.000	.000
835	0	.000	.000	.000	.000	.000
836	1	.967	.509	13.530	-13.480	.050
837	0	.000	.000	.000	.000	.000
838	0	.000	.000	.000	.000	.000
839	0	.000	.000	.000	.000	.000
840	2	1.057	.472	15.970	15.900	.070

	onset_percentage	duration_percentage	amplitude_percentage	P_V	onset
806	-2.5419	103.466	.169	.000	11.420
807	.0000	.000	.000	.000	.000
808	.0000	.000	.000	.000	.000
809	.0000	100.000	.076	.000	11.464
810	.0000	.000	.000	.000	.000
811	.0000	.000	.000	.000	.000
812	-5.6037	99.538	-.371	.000	11.367
813	.0000	.000	.000	.000	.000
814	.0000	.000	.000	.000	.000
815	.0000	.000	.000	.000	.000
816	-5.1415	99.538	.377	.000	11.375
817	.0000	.000	.000	.000	.000
818	1.9812	96.872	.338	.000	13.473
819	.0000	.000	.000	.000	.000
820	.0000	.000	.000	.000	.000
821	.0000	100.000	.076	.000	13.435
822	.0000	.000	.000	.000	.000
823	.0000	.000	.000	.000	.000
824	1.1992	85.975	-.222	.000	13.458
825	.0000	.000	.000	.000	.000
826	.0000	.000	.000	.000	.000
827	.0000	.000	.000	.000	.000
828	-4.6924	93.743	.440	.000	13.345
829	.0000	.000	.000	.000	.000
830	-3.1158	103.939	.085	.000	15.436
831	.0000	.000	.000	.000	.000
832	.0000	.000	.000	.000	.000
833	.0000	100.000	1.000	.000	15.489
834	.0000	.000	.000	.000	.000
835	.0000	.000	.000	.000	.000
836	.4115	86.772	-.371	.000	15.496
837	.0000	.000	.000	.000	.000
838	.0000	.000	.000	.000	.000
839	.0000	.000	.000	.000	.000
840	-2.6455	89.888	.440	.000	15.444

	pig_code	ID_gender	Pair_ID	Cycle_total	P_or_V	onset_ref
841	6238	0	1	9.000	0	0
842	6238	0	1	9.000	1	0
843	6238	0	3	9.000	0	0
844	6238	0	3	9.000	0	0
845	6238	0	5	9.000	1	0
846	6238	0	6	9.000	0	0
847	6238	0	7	9.000	0	0
848	6238	0	8	9.000	2	0
849	6238	0	9	9.000	0	0
850	6238	0	9	9.000	0	0
851	6238	0	11	9.000	0	0
852	6238	0	11	9.000	1	0
853	6238	0	1	10.000	0	0
854	6238	0	1	10.000	1	0
855	6238	0	3	10.000	0	0
856	6238	0	3	10.000	0	0
857	6238	0	5	10.000	1	0
858	6238	0	6	10.000	0	0
859	6238	0	7	10.000	0	0
860	6238	0	8	10.000	2	0
861	6238	0	9	10.000	0	0
862	6238	0	9	10.000	0	0
863	6238	0	11	10.000	0	0
864	6238	0	11	10.000	1	0
865	6238	0	1	11.000	0	0
866	6238	0	1	11.000	1	0
867	6238	0	3	11.000	0	0
868	6238	0	3	11.000	0	0
869	6238	0	5	11.000	1	0
870	6238	0	6	11.000	0	0
871	6238	0	7	11.000	0	0
872	6238	0	8	11.000	2	0
873	6238	0	9	11.000	0	0
874	6238	0	9	11.000	0	0
875	6238	0	11	11.000	0	0

	resp_duration	insp_duration	exp_duration	base_value	Peak_or_Valley	Amplitude
841	0	.000	.000	.000	.000	.000
842	2	1.079	.749	-11.790	11.810	.020
843	0	.000	.000	.000	.000	.000
844	0	.000	.000	.000	.000	.000
845	2	.877	.884	-26.250	26.280	.030
846	0	.000	.000	.000	.000	.000
847	0	.000	.000	.000	.000	.000
848	2	1.146	.488	13.520	-13.480	.040
849	0	.000	.000	.000	.000	.000
850	0	.000	.000	.000	.000	.000
851	0	.000	.000	.000	.000	.000
852	2	1.146	.532	15.960	15.910	.050
853	0	.000	.000	.000	.000	.000
854	2	.817	.832	-11.800	11.820	.020
855	0	.000	.000	.000	.000	.000
856	0	.000	.000	.000	.000	.000
857	2	.944	.772	-26.240	26.280	.040
858	0	.000	.000	.000	.000	.000
859	0	.000	.000	.000	.000	.000
860	1	1.012	.442	13.530	-13.500	.030
861	0	.000	.000	.000	.000	.000
862	0	.000	.000	.000	.000	.000
863	0	.000	.000	.000	.000	.000
864	2	1.064	.532	15.970	15.900	.070
865	0	.000	.000	.000	.000	.000
866	2	1.176	.667	-11.790	11.820	.030
867	0	.000	.000	.000	.000	.000
868	0	.000	.000	.000	.000	.000
869	2	1.124	.794	-26.250	26.280	.030
870	0	.000	.000	.000	.000	.000
871	0	.000	.000	.000	.000	.000
872	2	1.281	.495	13.520	-13.490	.030
873	0	.000	.000	.000	.000	.000
874	0	.000	.000	.000	.000	.000
875	0	.000	.000	.000	.000	.000

Sono respiration_Raw data.sav

	onset_percentage	duration_percentage	amplitude_percentage	P_V	onset
841	.0000	.000	.000	.000	.000
842	-4.6564	103.805	.169	.000	17.272
843	.0000	.000	.000	.000	.000
844	.0000	.000	.000	.000	.000
845	.0000	100.000	.114	.000	17.354
846	.0000	.000	.000	.000	.000
847	.0000	.000	.000	.000	.000
848	-1.2493	92.788	-.297	.000	17.332
849	.0000	.000	.000	.000	.000
850	.0000	.000	.000	.000	.000
851	.0000	.000	.000	.000	.000
852	-2.9529	95.287	.314	.000	17.302
853	.0000	.000	.000	.000	.000
854	4.8368	96.096	.169	.000	19.318
855	.0000	.000	.000	.000	.000
856	.0000	.000	.000	.000	.000
857	.0000	100.000	.152	.000	19.235
858	.0000	.000	.000	.000	.000
859	.0000	.000	.000	.000	.000
860	4.3706	84.732	-.222	.000	19.310
861	.0000	.000	.000	.000	.000
862	.0000	.000	.000	.000	.000
863	.0000	.000	.000	.000	.000
864	.4662	93.007	.440	.000	19.243
865	.0000	.000	.000	.000	.000
866	6.6736	96.090	.254	.000	21.236
867	.0000	.000	.000	.000	.000
868	.0000	.000	.000	.000	.000
869	.0000	100.000	.114	.000	21.108
870	.0000	.000	.000	.000	.000
871	.0000	.000	.000	.000	.000
872	1.5641	92.596	-.222	.000	21.138
873	.0000	.000	.000	.000	.000
874	.0000	.000	.000	.000	.000
875	.0000	.000	.000	.000	.000

	pig_code	ID_gender	Pair_ID	Cycle_total	P_or_V	onset_ref
876	6238	0	11	11.000	1	0
877	6238	0	1	12.000	0	0
878	6238	0	1	12.000	1	0
879	6238	0	3	12.000	0	0
880	6238	0	3	12.000	0	0
881	?	?	?	?	?	?
882	?	?	?	?	?	?
883	6238	0	7	12.000	0	0
884	6238	0	8	12.000	2	0
885	6238	0	9	12.000	0	0
886	6238	0	9	12.000	0	0
887	6238	0	11	12.000	0	0
888	6238	0	11	12.000	1	0
889	6238	0	1	13.000	0	0
890	6238	0	1	13.000	1	0
891	6238	0	3	13.000	0	0
892	6238	0	3	13.000	0	0
893	6238	0	5	13.000	1	0
894	6238	0	6	13.000	0	0
895	6238	0	7	13.000	0	0
896	6238	0	8	13.000	2	0
897	6238	0	9	13.000	0	0
898	6238	0	9	13.000	0	0
899	6238	0	11	13.000	0	0
900	6238	0	11	13.000	1	0
901	?	?	?	?	?	?
902	?	?	?	?	?	?
903	?	?	?	?	?	?
904	?	?	?	?	?	?
905	?	?	?	?	?	?
906	?	?	?	?	?	?
907	?	?	?	?	?	?
908	?	?	?	?	?	?
909	?	?	?	?	?	?
910	?	?	?	?	?	?

Sono respiration_Raw data.sav

	resp_duration	insp_duration	exp_duration	base_value	Peak_or_Valley	Amplitude
876	2	1.289	.539	15.970	15.900	.070
877	0	.000	.000	.000	.000	.000
878	2	1.012	.884	-11.800	11.810	.010
879	0	.000	.000	.000	.000	.000
880	0	.000	.000	.000	.000	.000
881	2	1.057	.779	-26.250	26.280	.030
882	0	.000	.000	.000	.000	.000
883	0	.000	.000	.000	.000	.000
884	2	1.139	.510	13.530	-13.490	.040
885	0	.000	.000	.000	.000	.000
886	0	.000	.000	.000	.000	.000
887	0	.000	.000	.000	.000	.000
888	2	1.094	.592	15.970	15.900	.070
889	0	.000	.000	.000	.000	.000
890	2	.922	.869	-11.810	11.820	.010
891	0	.000	.000	.000	.000	.000
892	0	.000	.000	.000	.000	.000
893	2	.884	.839	-26.250	26.280	.030
894	0	.000	.000	.000	.000	.000
895	0	.000	.000	.000	.000	.000
896	2	1.087	.494	13.530	-13.480	.050
897	0	.000	.000	.000	.000	.000
898	0	.000	.000	.000	.000	.000
899	0	.000	.000	.000	.000	.000
900	2	1.019	.562	15.970	15.900	.070
901	0	.000	.000	.000	?	?
902	2	1.109	.757	-11.790	?	?
903	0	.000	.000	.000	?	?
904	0	.000	.000	.000	?	?
905	2	1.057	.726	-26.250	?	?
906	0	.000	.000	.000	?	?
907	0	.000	.000	.000	?	?
908	2	1.094	.555	13.530	?	?
909	0	.000	.000	.000	?	?
910	0	.000	.000	.000	?	?

Sono respiration_Raw data.sav

	onset_percentage	duration_percentage	amplitude_percentage	P_V	onset
876	.4171	95.308	.440	.000	21.116
877	.0000	.000	.000	.000	.000
878	-3.2680	103.268	.085	.000	23.177
879	.0000	.000	.000	.000	.000
880	.0000	.000	.000	.000	.000
881	.0000	100.000	.114	.000	23.237
882	.0000	.000	.000	.000	.000
883	.0000	.000	.000	.000	.000
884	.3813	89.815	-.297	.000	23.244
885	.0000	.000	.000	.000	.000
886	.0000	.000	.000	.000	.000
887	.0000	.000	.000	.000	.000
888	.0000	91.830	.440	.000	23.214
889	.0000	.000	.000	.000	.000
890	-4.3529	103.947	.085	.000	25.207
891	.0000	.000	.000	.000	.000
892	.0000	.000	.000	.000	.000
893	.0000	100.000	.114	.000	25.282
894	.0000	.000	.000	.000	.000
895	.0000	.000	.000	.000	.000
896	-5.2234	91.759	-.371	.000	25.192
897	.0000	.000	.000	.000	.000
898	.0000	.000	.000	.000	.000
899	.0000	.000	.000	.000	.000
900	-3.4823	91.759	.440	.000	25.222
901	.0000	.000	.000	.000	.000
902	2.1312	104.655	.254	.000	27.126
903	.0000	.000	.000	.000	.000
904	.0000	.000	.000	.000	.000
905	.0000	100.000	.076	.000	27.088
906	.0000	.000	.000	.000	.000
907	.0000	.000	.000	.000	.000
908	-2.0752	92.485	-.371	.000	27.051
909	.0000	.000	.000	.000	.000
910	.0000	.000	.000	.000	.000

	pig_code	ID_gender	Pair_ID	Cycle_total	P_or_V	onset_ref
911	6238	0	11	14.000	0	0
912	6238	0	11	14.000	1	0
913	6238	0	1	15.000	0	0
914	6238	0	1	15.000	1	0
915	6238	0	3	15.000	0	0
916	6238	0	3	15.000	0	0
917	6238	0	5	15.000	1	0
918	6238	0	6	15.000	0	0
919	6238	0	7	15.000	0	0
920	6238	0	8	15.000	2	0
921	6238	0	9	15.000	0	0
922	6238	0	9	15.000	0	0
923	6238	0	11	15.000	0	0
924	6238	0	11	15.000	1	0
925	6238	1	1	15.000	0	0
926	6238	1	1	15.000	1	0
927	6238	1	3	15.000	0	0
928	6238	1	4	15.000	0	0
929	6238	1	5	15.000	1	0
930	6238	1	6	15.000	0	0
931	6238	1	7	15.000	0	0
932	6238	1	8	15.000	2	0
933	6238	1	9	15.000	0	0
934	6238	1	10	15.000	0	0
935	6238	1	11	15.000	0	0
936	6238	1	11	15.000	1	0
937	.	.	.	.	.	.
938	.	.	.	.	.	.

	resp_duration	insp_duration	exp_duration	base_value	Peak_or_Valley	Amplitude
911	0	.000	.000	.000	.000	.000
912	2	1.116	.585	15.970	15.900	.070
913	0	.000	.000	.000	.000	.000
914	2	1.094	.689	-11.790	11.810	.020
915	0	.000	.000	.000	.000	.000
916	0	.000	.000	.000	.000	.000
917	2	1.042	.741	-26.250	26.280	.030
918	0	.000	.000	.000	.000	.000
919	0	.000	.000	.000	.000	.000
920	2	1.072	.562	13.530	-13.480	.050
921	0	.000	.000	.000	.000	.000
922	0	.000	.000	.000	.000	.000
923	0	.000	.000	.000	.000	.000
924	2	1.034	.532	15.970	15.900	.070
925	0	.000	.000	.000	.000	.000
926	2	1.094	.689	-11.790	11.810	.020
927	0	.000	.000	.000	.000	.000
928	0	.000	.000	.000	.000	.000
929	2	1.042	.741	-26.250	26.280	.030
930	0	.000	.000	.000	.000	.000
931	0	.000	.000	.000	.000	.000
932	2	1.072	.562	13.530	-13.480	.050
933	0	.000	.000	.000	.000	.000
934	0	.000	.000	.000	.000	.000
935	0	.000	.000	.000	.000	.000
936	2	1.034	.532	15.970	15.900	.070
937	.	.	.	.	.	.
938	.	.	.	.	.	.

	onset_percentage	duration_percentage	amplitude_percentage	P_V	onset
911	.0000	.000	.000	.000	.000
912	-3.7577	95.401	.440	.000	27.021
913	.0000	.000	.000	.000	.000
914	-2.0752	100.000	.169	.000	29.029
915	.0000	.000	.000	.000	.000
916	.0000	.000	.000	.000	.000
917	.0000	100.000	.114	.000	29.066
918	.0000	.000	.000	.000	.000
919	.0000	.000	.000	.000	.000
920	-3.3651	91.643	-.371	.000	29.006
921	.0000	.000	.000	.000	.000
922	.0000	.000	.000	.000	.000
923	.0000	.000	.000	.000	.000
924	.0000	87.830	.440	.000	29.036
925	.0000	.000	.000	.000	.000
926	-2.0752	100.000	.169	.000	29.029
927	.0000	.000	.000	.000	.000
928	.0000	.000	.000	.000	.000
929	.0010	100.000	.114	.000	29.066
930	.0000	.000	.000	.000	.000
931	.0000	.000	.000	.000	.000
932	-3.3651	91.643	-.371	.000	29.006
933	.0000	.000	.000	.000	.000
934	.0000	.000	.000	.000	.000
935	.0000	.000	.000	.000	.000
936	.0000	87.830	-.440	.000	29.036
937	.	.	.	.	.
938	.	.	.	.	.
