# Supplementary material for: The dynamic shape changes of the tongue base during respiration, chewing and swallowing
Source: PLoS One. 2025 Apr 14;20(4):e0315885. doi: 10.1371/journal.pone.0315885 (PMC11996074; doi:10.1371/journal.pone.0315885)
Supplement: S2 File — (PDF) [file pone.0315885.s002.pdf]

Sono Mastication_Raw data.sav

	Pig_chw	Gender_chw	Pair_chw	Cycles	P_V_chw	Onset_chw
1	5518	1	1.00	1.00	0	.00
2	5518	1	2.00	1.00	0	.00
3	5518	1	3.00	1.00	2	-.11
4	5518	1	4.00	1.00	0	.00
5	5518	1	5.00	1.00	1	.00
6	5518	1	6.00	1.00	0	.00
7	5518	1	7.00	1.00	1	.02
8	5518	1	8.00	1.00	0	.00
9	5518	1	9.00	1.00	0	.00
10	5518	1	10.00	1.00	0	.00
11	5518	1	11.00	1.00	0	.00
12	5518	1	12.00	1.00	0	.00
13	5518	1	1.00	2.00	0	.00
14	5518	1	2.00	2.00	0	.00
15	5518	1	3.00	2.00	2	-.13
16	5518	1	4.00	2.00	0	.00
17	5518	1	5.00	2.00	1	.00
18	5518	1	6.00	2.00	0	.00
19	5518	1	7.00	2.00	1	.01
20	5518	1	8.00	2.00	0	.00
21	5518	1	9.00	2.00	0	.00
22	5518	1	10.00	2.00	0	.00
23	5518	1	11.00	2.00	0	.00
24	5518	1	12.00	2.00	0	.00
25	5518	1	1.00	3.00	0	.00
26	5518	1	2.00	3.00	0	.00
27	5518	1	3.00	3.00	2	-.08
28	5518	1	4.00	3.00	0	.00
29	5518	1	5.00	3.00	1	.00
30	5518	1	6.00	3.00	0	.00
31	5518	1	7.00	3.00	1	-.02
32	5518	1	8.00	3.00	0	.00
33	5518	1	9.00	3.00	0	.00
34	5518	1	10.00	3.00	0	.00
35	5518	1	11.00	3.00	0	.00

Sono Mastication_Raw data.sav

	Duration_chw	P_or_V_value_chw	Base_value_chw	Amplitude	onset_percentage_chw	duration_percentage_chw
1	.00	.00	.00	.00	.00	.00
2	.00	.00	.00	.00	.00	.00
3	.67	-12.13	13.49	1.36	-18.24	108.63
4	.00	.00	.00	.00	.00	.00
5	.61	20.17	-16.68	3.49	.02	100.00
6	.00	.00	.00	.00	.00	.00
7	.53	22.23	-18.84	3.39	3.75	86.65
8	.00	.00	.00	.00	.00	.00
9	.00	.00	.00	.00	.00	.00
10	.00	.00	.00	.00	.00	.00
11	.00	.00	.00	.00	.00	.00
12	.00	.00	.00	.00	.00	.00
13	.00	.00	.00	.00	.00	.00
14	.00	.00	.00	.00	.00	.00
15	.75	-12.15	13.64	1.49	-19.88	111.13
16	.00	.00	.00	.00	.00	.00
17	.67	19.96	-16.96	3.00	.02	100.00
18	.00	.00	.00	.00	.00	.00
19	.64	21.94	-18.96	2.98	1.48	95.55
20	.00	.00	.00	.00	.00	.00
21	.00	.00	.00	.00	.00	.00
22	.00	.00	.00	.00	.00	.00
23	.00	.00	.00	.00	.00	.00
24	.00	.00	.00	.00	.00	.00
25	.00	.00	.00	.00	.00	.00
26	.00	.00	.00	.00	.00	.00
27	.64	-12.46	13.84	1.38	-14.39	110.40
28	.00	.00	.00	.00	.00	.00
29	.58	19.79	-16.86	2.93	.02	100.00
30	.00	.00	.00	.00	.00	.00
31	.54	21.56	-19.20	2.36	-2.60	93.59
32	.00	.00	.00	.00	.00	.00
33	.00	.00	.00	.00	.00	.00
34	.00	.00	.00	.00	.00	.00
35	.00	.00	.00	.00	.00	.00

Sono Mastication_Raw data.sav

	amplitude_perc	chewing_side	Pig_code_sw	gender_sw	Pair_sw	Sw_episodes
1	.00	.00	5518	1	1.00	2.00
2	.00	.00	5518	1	2.00	2.00
3	-11.21	2.00	5518	1	3.00	2.00
4	.00	.00	5518	1	4.00	2.00
5	17.30	5.00	5518	1	5.00	2.00
6	.00	.00	5518	1	6.00	2.00
7	15.25	5.00	5518	1	7.00	2.00
8	.00	.00	5518	1	8.00	2.00
9	.00	.00	5518	1	9.00	2.00
10	.00	.00	5518	1	10.00	2.00
11	.00	.00	5518	1	11.00	2.00
12	.00	.00	5518	1	12.00	2.00
13	.00	.00	5773	2	1.00	3.00
14	.00	.00	5773	2	2.00	3.00
15	-12.26	2.00	5773	2	3.00	3.00
16	.00	.00	5773	2	4.00	3.00
17	15.03	5.00	5773	2	5.00	3.00
18	.00	.00	5773	2	6.00	3.00
19	13.58	5.00	5773	2	7.00	3.00
20	.00	.00	5773	2	8.00	3.00
21	.00	.00	5773	2	9.00	3.00
22	.00	.00	5773	2	10.00	3.00
23	.00	.00	5773	2	11.00	3.00
24	.00	.00	5773	2	12.00	3.00
25	.00	.00	5794	2	1.00	2.00
26	.00	.00	5794	2	2.00	2.00
27	-11.08	2.00	5794	2	3.00	2.00
28	.00	.00	5794	2	4.00	2.00
29	14.81	5.00	5794	2	5.00	2.00
30	.00	.00	5794	2	6.00	2.00
31	10.95	5.00	5794	2	7.00	2.00
32	.00	.00	5794	2	8.00	2.00
33	.00	.00	5794	2	9.00	2.00
34	.00	.00	5794	2	10.00	2.00
35	.00	.00	5794	2	11.00	2.00

	P_V_sw	Onset_sw	duration_sw	P_or_V_value_sw	Base_value_sw	Range
1	0	.00	.00	.00	.00	.00
2	0	.00	.00	.00	.00	.00
3	2	.06	.45	-12.15	12.66	.51
4	0	.00	.00	.00	.00	.00
5	1	.00	.54	19.97	-17.78	2.19
6	0	.00	.00	.00	.00	.00
7	1	-.06	.54	22.27	-20.03	2.24
8	0	.00	.00	.00	.00	.00
9	1	.11	.63	6.54	-6.01	.53
10	0	.00	.00	.00	.00	.00
11	0	.00	.00	.00	.00	.00
12	0	.00	.00	.00	.00	.00
13	1	-.06	.77	8.14	-6.95	1.19
14	0	.00	.00	.00	.00	.00
15	0	.00	.00	.00	.00	.00
16	1	.01	.62	19.34	-19.09	.25
17	1	.00	.74	18.35	-17.42	.93
18	0	.00	.00	.00	.00	.00
19	1	-.01	.48	24.24	-23.14	1.10
20	0	.00	.00	.00	.00	.00
21	0	.00	?	?	?	?
22	0	.00	?	?	?	?
23	0	.00	?	?	?	?
24	0	.00	?	?	?	?
25	1	-.03	?	?	?	?
26	1	-.09	?	?	?	?
27	2	-.13	?	?	?	?
28	1	.00	?	?	?	?
29	1	.00	?	?	?	?
30	1	-.05	?	?	?	?
31	1	-.05	?	?	?	?
32	0	.00	?	?	?	?
33	1	-.02	?	?	?	?
34	1	-.11	?	?	?	?
35	0	.00	?	?	?	?

	onset_percent_sw	duration_percent_sw	Range_percent	Pause_before_sw	Pause_after_sw
1	.00	.00	.00	.00	.00
2	.00	.00	.00	.00	.00
3	11.61	85.20	-4.16	6.91	15.01
4	.00	.00	.00	.00	.00
5	.19	102.01	10.96	7.73	16.20
6	.00	.00	.00	.00	.00
7	-12.53	101.97	10.05	7.51	15.89
8	.00	.00	.00	.00	.00
9	18.45	114.99	8.10	3.54	3.31
10	.00	.00	.00	.00	.00
11	.00	.00	.00	.00	.00
12	.00	.00	.00	.00	.00
13	-8.03	104.86	14.62	2.16	1.23
14	.00	.00	.00	.00	.00
15	.00	.00	.00	.00	.00
16	1.25	72.56	1.27	2.03	1.21
17	.14	100.00	5.05	2.32	1.49
18	.00	.00	.00	.00	.00
19	-1.89	68.43	4.54	1.91	1.50
20	.00	.00	.00	.00	.00
21	.00	.00	.00	.00	.00
22	.00	.00	.00	.00	.00
23	.00	.00	.00	.00	.00
24	.00	.00	.00	.00	.00
25	-3.20	86.34	11.30	2.30	1.89
26	-11.00	106.30	2.80	1.77	1.41
27	-17.44	121.53	.60	.77	.61
28	.11	100.00	12.12	2.15	1.72
29	.14	100.00	3.76	3.19	1.09
30	-4.80	101.60	15.46	2.31	1.81
31	-7.60	112.94	4.62	1.60	1.20
32	.00	.00	.00	.00	.00
33	-2.04	110.22	1.16	.97	1.14
34	-14.39	120.62	7.67	1.60	1.23
35	.00	.00	.00	.00	.00

Sono Mastication_Raw data.sav

	Pig_chw	Gender_chw	Pair_chw	Cycles	P_V_chw	Onset_chw
36	5518	1	12.00	3.00	0	.00
37	5518	1	1.00	4.00	0	.00
38	5518	1	2.00	4.00	0	.00
39	5518	1	3.00	4.00	2	-.09
40	5518	1	4.00	4.00	0	.00
41	5518	1	5.00	4.00	1	.00
42	5518	1	6.00	4.00	0	.00
43	5518	1	7.00	4.00	1	-.08
44	5518	1	8.00	4.00	0	.00
45	5518	1	9.00	4.00	0	.00
46	5518	1	10.00	4.00	0	.00
47	5518	1	11.00	4.00	0	.00
48	5518	1	12.00	4.00	0	.00
49	5518	1	1.00	5.00	0	.00
50	5518	1	2.00	5.00	0	.00
51	5518	1	3.00	5.00	2	-.17
52	5518	1	4.00	5.00	0	.00
53	5518	1	5.00	5.00	1	.00
54	5518	1	6.00	5.00	0	.00
55	5518	1	7.00	5.00	1	.10
56	5518	1	8.00	5.00	0	.00
57	5518	1	9.00	5.00	0	.00
58	5518	1	10.00	5.00	0	.00
59	5518	1	11.00	5.00	0	.00
60	5518	1	12.00	5.00	0	.00
61	5518	1	1.00	6.00	0	.00
62	5518	1	2.00	6.00	0	.00
63	5518	1	3.00	6.00	2	-.13
64	5518	1	4.00	6.00	0	.00
65	5518	1	5.00	6.00	1	.00
66	5518	1	6.00	6.00	0	.00
67	5518	1	7.00	6.00	1	-.03
68	5518	1	8.00	6.00	0	.00
69	5518	1	9.00	6.00	0	.00
70	5518	1	10.00	6.00	0	.00

	Duration_chw	P_or_V_value_chw	Base_value_chw	Amplitude	onset_percentage_chw	duration_percentage_chw
36	.00	.00	.00	.00	.00	.00
37	.00	.00	.00	.00	.00	.00
38	.00	.00	.00	.00	.00	.00
39	.62	-12.57	14.12	1.55	-13.35	92.29
40	.00	.00	.00	.00	.00	.00
41	.67	19.82	-16.66	3.16	.02	100.00
42	.00	.00	.00	.00	.00	.00
43	.59	21.50	-19.00	2.50	-11.13	87.83
44	.00	.00	.00	.00	.00	.00
45	.00	.00	.00	.00	.00	.00
46	.00	.00	.00	.00	.00	.00
47	.00	.00	.00	.00	.00	.00
48	.00	.00	.00	.00	.00	.00
49	.00	.00	.00	.00	.00	.00
50	.00	.00	.00	.00	.00	.00
51	.79	-11.90	13.94	2.04	-24.57	111.79
52	.00	.00	.00	.00	.00	.00
53	.70	20.40	-16.63	3.77	.01	100.00
54	.00	.00	.00	.00	.00	.00
55	.52	22.39	-18.63	3.76	14.77	73.44
56	.00	.00	.00	.00	.00	.00
57	.00	.00	.00	.00	.00	.00
58	.00	.00	.00	.00	.00	.00
59	.00	.00	.00	.00	.00	.00
60	.00	.00	.00	.00	.00	.00
61	.00	.00	.00	.00	.00	.00
62	.00	.00	.00	.00	.00	.00
63	.71	-12.55	13.97	1.42	-20.68	115.96
64	.00	.00	.00	.00	.00	.00
65	.61	19.84	-16.67	3.17	.02	100.00
66	.00	.00	.00	.00	.00	.00
67	.61	21.53	-18.77	2.76	-4.89	98.86
68	.00	.00	.00	.00	.00	.00
69	.00	.00	.00	.00	.00	.00
70	.00	.00	.00	.00	.00	.00

Sono Mastication_Raw data.sav

	amplitude_perc	chewing_side	Pig_code_sw	gender_sw	Pair_sw	Sw_episodes
36	.00	.00	5794	2	12.00	2.00
37	.00	.00	6487	2	1.00	2.00
38	.00	.00	6487	2	2.00	2.00
39	-12.33	2.00	6487	2	3.00	2.00
40	.00	.00	6487	2	4.00	2.00
41	15.94	5.00	6487	2	5.00	2.00
42	.00	.00	6487	2	6.00	2.00
43	11.63	5.00	6487	2	7.00	2.00
44	.00	.00	6487	2	8.00	2.00
45	.00	.00	6487	2	9.00	2.00
46	.00	.00	6487	2	10.00	2.00
47	.00	.00	6487	2	11.00	2.00
48	.00	.00	6487	2	12.00	2.00
49	.00	.00	6238	1	1.00	1.00
50	.00	.00	6238	1	2.00	1.00
51	-17.14	4.00	6238	1	3.00	1.00
52	.00	.00	6238	1	4.00	1.00
53	18.48	5.00	6238	1	5.00	1.00
54	.00	.00	6238	1	6.00	1.00
55	16.79	5.00	6238	1	7.00	1.00
56	.00	.00	6238	1	8.00	1.00
57	.00	.00	6238	1	9.00	1.00
58	.00	.00	6238	1	10.00	1.00
59	.00	.00	6238	1	11.00	1.00
60	.00	.00	6238	1	12.00	1.00
61	.00	.00	5824	1	1.00	.00
62	.00	.00	5824	1	2.00	.00
63	-11.32	4.00	5824	1	3.00	.00
64	.00	.00	5824	1	4.00	.00
65	15.98	5.00	5824	1	5.00	.00
66	.00	.00	5824	1	6.00	.00
67	12.82	5.00	5824	1	7.00	.00
68	.00	.00	5824	1	8.00	.00
69	.00	.00	5824	1	9.00	.00
70	.00	.00	5824	1	10.00	.00

	P_V_sw	Onset_sw	duration_sw	P_or_V_value_sw	Base_value_sw	Range
36	0	.00	.00	.00	.00	.00
37	0	.00	.00	.00	.00	.00
38	0	.05	.56	37.49	.26	37.74
39	0	.00	.00	.00	.00	.00
40	0	.00	.00	.00	.00	.00
41	0	.00	.60	29.34	-27.32	2.03
42	0	.00	.00	.00	.00	.00
43	0	.00	.00	.00	.00	.00
44	0	.00	.00	.00	.00	.00
45	0	-.03	.60	1.78	.57	2.35
46	0	.00	.00	.00	.00	.00
47	0	.00	.00	.00	.00	.00
48	0	.00	.00	.00	.00	.00
49	0	.00	.00	.00	.00	.00
50	0	.00	.00	.00	.00	.00
51	2	-.05	.64	-12.43	13.85	1.42
52	1	-.04	.53	16.41	-15.49	.92
53	1	.00	.62	23.65	-21.13	2.52
54	1	.07	.55	27.93	-25.76	2.17
55	0	.00	.00	.00	.00	.00
56	1	-.02	.57	13.95	-12.08	1.87
57	0	.00	.00	.00	.00	.00
58	0	.00	.00	.00	.00	.00
59	1	-.04	.64	5.09	-4.59	.50
60	2	-.05	.62	-12.97	14.94	1.98
61	0	.00	?	?	?	?
62	0	.00	?	?	?	?
63	0	.00	?	?	?	?
64	0	.00	?	?	?	?
65	0	.00	?	?	?	?
66	0	.00	?	?	?	?
67	0	.00	?	?	?	?
68	0	.00	?	?	?	?
69	0	.00	?	?	?	?
70	0	.00	?	?	?	?

	onset_percent_sw	duration_percent_sw	Range_percent	Pause_before_sw	Pause_after_sw
36	.00	.00	.00	.00	.00
37	.00	.00	.00	.00	.00
38	9.92	91.49	101.87	.94	2.56
39	.00	.00	.00	.00	.00
40	.00	.00	.00	.00	.00
41	.17	100.00	6.92	.85	2.56
42	.00	.00	.00	.00	.00
43	.00	.00	.00	.00	.00
44	.00	.00	.00	.00	.00
45	-2.20	98.89	-10.07	.82	2.83
46	.00	.00	.00	.00	.00
47	.00	.00	.00	.00	.00
48	.00	.00	.00	.00	.00
49	.00	.00	.00	.00	.00
50	.00	.00	.00	.00	.00
51	-8.21	103.50	-11.42	2.18	2.08
52	-6.80	85.41	5.60	1.45	1.39
53	.16	100.00	10.66	1.47	1.83
54	13.13	88.13	7.77	1.91	1.51
55	.00	.00	.00	.00	.00
56	-3.85	92.18	13.39	1.49	1.26
57	.00	.00	.00	.00	.00
58	.00	.00	.00	.00	.00
59	-6.32	103.76	9.84	1.17	1.54
60	-7.90	99.45	-15.22	1.19	2.04
61	.00	.00	.00	.00	.00
62	.00	.00	.00	.00	.00
63	.00	.00	.00	.00	.00
64	.00	.00	.00	.00	.00
65	.00	.00	.00	.00	.00
66	.00	.00	.00	.00	.00
67	.00	.00	.00	.00	.00
68	.00	.00	.00	.00	.00
69	.00	.00	.00	.00	.00
70	.00	.00	.00	.00	.00

Sono Mastication_Raw data.sav

	Pig_chw	Gender_chw	Pair_chw	Cycles	P_V_chw	Onset_chw
71	5518	1	11.00	6.00	0	.00
72	5518	1	12.00	6.00	0	.00
73	5518	1	1.00	7.00	0	.00
74	5518	1	2.00	7.00	0	.00
75	5518	1	3.00	7.00	2	-.09
76	5518	1	4.00	7.00	0	.00
77	5518	1	5.00	7.00	1	.00
78	5518	1	6.00	7.00	0	.00
79	5518	1	7.00	7.00	1	.02
80	5518	1	8.00	7.00	0	.00
81	5518	1	9.00	7.00	0	.00
82	5518	1	10.00	7.00	0	.00
83	5518	1	11.00	7.00	0	.00
84	5518	1	12.00	7.00	0	.00
85	5518	1	1.00	8.00	0	.00
86	5518	1	2.00	8.00	0	.00
87	5518	1	3.00	8.00	2	-.04
88	5518	1	4.00	8.00	0	.00
89	5518	1	5.00	8.00	1	.00
90	5518	1	6.00	8.00	0	.00
91	5518	1	7.00	8.00	1	-.03
92	5518	1	8.00	8.00	0	.00
93	5518	1	9.00	8.00	0	.00
94	5518	1	10.00	8.00	0	.00
95	5518	1	11.00	8.00	0	.00
96	5518	1	12.00	8.00	0	.00
97	5518	1	1.00	9.00	0	.00
98	5518	1	2.00	9.00	0	.00
99	5518	1	3.00	9.00	2	-.07
100	5518	1	4.00	9.00	0	.00
101	?	?	?	?	?	?
102	?	?	?	?	?	?
103	?	?	?	?	?	?
104	?	?	?	?	?	?
105	?	?	?	?	?	?

	Duration_chw	P_or_V_value_chw	Base_value_chw	Amplitude	onset_percentage_chw	duration_percentage_chw
71	.00	.00	.00	.00	.00	.00
72	.00	.00	.00	.00	.00	.00
73	.00	.00	.00	.00	.00	.00
74	.00	.00	.00	.00	.00	.00
75	.70	-12.11	14.10	1.99	-12.91	100.00
76	.00	.00	.00	.00	.00	.00
77	.70	20.38	-16.39	3.99	.01	100.00
78	.00	.00	.00	.00	.00	.00
79	.58	22.67	-18.66	4.01	2.15	83.79
80	.00	.00	.00	.00	.00	.00
81	.00	.00	.00	.00	.00	.00
82	.00	.00	.00	.00	.00	.00
83	.00	.00	.00	.00	.00	.00
84	.00	.00	.00	.00	.00	.00
85	.00	.00	.00	.00	.00	.00
86	.00	.00	.00	.00	.00	.00
87	.64	-12.88	13.95	1.07	-5.57	94.43
88	.00	.00	.00	.00	.00	.00
89	.68	20.09	-16.97	3.12	.02	100.00
90	.00	.00	.00	.00	.00	.00
91	.67	22.05	-19.39	2.66	-4.40	98.83
92	.00	.00	.00	.00	.00	.00
93	.00	.00	.00	.00	.00	.00
94	.00	.00	.00	.00	.00	.00
95	.00	.00	.00	.00	.00	.00
96	.00	.00	.00	.00	.00	.00
97	.00	.00	.00	.00	.00	.00
98	.00	.00	.00	.00	.00	.00
99	.67	-12.26	14.09	1.83	-10.09	100.00
100	.00	.00	.00	.00	.00	.00
101	.67	20.43	-17.05	3.38	?	?
102	.00	.00	.00	.00	?	?
103	.67	22.79	-19.17	3.62	?	?
104	.00	.00	.00	.00	?	?
105	.00	.00	.00	.00	?	?

	amplitude_perc	chewing_side	Pig_code_sw	gender_sw	Pair_sw	Sw_episodes
71	.00	.00	5824	1	11.00	.00
72	.00	.00	5824	1	12.00	.00
73	.00	.00	5564	2	1.00	.00
74	.00	.00	5564	2	2.00	.00
75	-16.43	4.00	5564	2	3.00	.00
76	.00	.00	5564	2	4.00	.00
77	19.58	5.00	5564	2	5.00	.00
78	.00	.00	5564	2	6.00	.00
79	17.69	5.00	5564	2	7.00	.00
80	.00	.00	5564	2	8.00	.00
81	.00	.00	5564	2	9.00	.00
82	.00	.00	5564	2	10.00	.00
83	.00	.00	5564	2	11.00	.00
84	.00	.00	5564	2	12.00	.00
85	.00	.00	5907	1	1.00	.00
86	.00	.00	5907	1	2.00	.00
87	-8.31	4.00	5907	1	3.00	.00
88	.00	.00	5907	1	4.00	.00
89	15.53	5.00	5907	1	5.00	.00
90	.00	.00	5907	1	6.00	.00
91	12.06	5.00	5907	1	7.00	.00
92	.00	.00	5907	1	8.00	.00
93	.00	.00	5907	1	9.00	.00
94	.00	.00	5907	1	10.00	.00
95	.00	.00	5907	1	11.00	.00
96	.00	.00	5907	1	12.00	.00
97	.00	.00	.	.	.	.
98	.00	.00	.	.	.	.
99	-14.93	4.00	.	.	.	.
100	.00	.00	.	.	.	.
101	16.54	5.00	.	.	.	.
102	.00	.00	.	.	.	.
103	15.88	5.00	.	.	.	.
104	.00	.00	.	.	.	.
105	.00	.00	.	.	.	.

Sono Mastication_Raw data.sav

	P_V_sw	Onset_sw	duration_sw	P_or_V_value_sw	Base_value_sw	Range
71	0	.00	.00	.00	.00	.00
72	0	.00	.00	.00	.00	.00
73	0	.00	.00	.00	.00	.00
74	0	.00	.00	.00	.00	.00
75	0	.00	.00	.00	.00	.00
76	0	.00	.00	.00	.00	.00
77	0	.00	.00	.00	.00	.00
78	0	.00	.00	.00	.00	.00
79	0	.00	.00	.00	.00	.00
80	0	.00	.00	.00	.00	.00
81	0	.00	.00	.00	.00	.00
82	0	.00	.00	.00	.00	.00
83	0	.00	.00	.00	.00	.00
84	0	.00	.00	.00	.00	.00
85	0	.00	.00	.00	.00	.00
86	0	.00	.00	.00	.00	.00
87	0	.00	.00	.00	.00	.00
88	0	.00	.00	.00	.00	.00
89	0	.00	.00	.00	.00	.00
90	0	.00	.00	.00	.00	.00
91	0	.00	.00	.00	.00	.00
92	0	.00	.00	.00	.00	.00
93	0	.00	.00	.00	.00	.00
94	0	.00	.00	.00	.00	.00
95	0	.00	.00	.00	.00	.00
96	0	.00	.00	.00	.00	.00
97	.	.	.	.	.	.
98	.	.	.	.	.	.
99	.	.	.	.	.	.
100	.	.	.	.	.	.
101	.	.	?	?	?	?
102	.	.	?	?	?	?
103	.	.	?	?	?	?
104	.	.	?	?	?	?
105	.	.	?	?	?	?

	onset_percent_sw	duration_percent_sw	Range_percent	Pause_before_sw	Pause_after_sw
71	.00	.00	.00	.00	.00
72	.00	.00	.00	.00	.00
73	.00	.00	.00	.00	.00
74	.00	.00	.00	.00	.00
75	.00	.00	.00	.00	.00
76	.00	.00	.00	.00	.00
77	.00	.00	.00	.00	.00
78	.00	.00	.00	.00	.00
79	.00	.00	.00	.00	.00
80	.00	.00	.00	.00	.00
81	.00	.00	.00	.00	.00
82	.00	.00	.00	.00	.00
83	.00	.00	.00	.00	.00
84	.00	.00	.00	.00	.00
85	.00	.00	.00	.00	.00
86	.00	.00	.00	.00	.00
87	.00	.00	.00	.00	.00
88	.00	.00	.00	.00	.00
89	.00	.00	.00	.00	.00
90	.00	.00	.00	.00	.00
91	.00	.00	.00	.00	.00
92	.00	.00	.00	.00	.00
93	.00	.00	.00	.00	.00
94	.00	.00	.00	.00	.00
95	.00	.00	.00	.00	.00
96	.00	.00	.00	.00	.00
97	.	.	.	.	.
98	.	.	.	.	.
99	.	.	.	.	.
100	.	.	.	.	.
101	.	.	.	.	.
102	.	.	.	.	.
103	.	.	.	.	.
104	.	.	.	.	.
105	.	.	.	.	.

Sono Mastication_Raw data.sav

	Pig_chw	Gender_chw	Pair_chw	Cycles	P_V_chw	Onset_chw
106	5518	1	10.00	9.00	0	.00
107	5518	1	11.00	9.00	0	.00
108	5518	1	12.00	9.00	0	.00
109	5518	1	1.00	10.00	0	.00
110	5518	1	2.00	10.00	0	.00
111	5518	1	3.00	10.00	2	-.17
112	5518	1	4.00	10.00	0	.00
113	5518	1	5.00	10.00	1	.00
114	5518	1	6.00	10.00	0	.00
115	5518	1	7.00	10.00	1	.01
116	5518	1	8.00	10.00	0	.00
117	5518	1	9.00	10.00	0	.00
118	5518	1	10.00	10.00	0	.00
119	5518	1	11.00	10.00	0	.00
120	5518	1	12.00	10.00	0	.00
121	5518	1	1.00	11.00	0	.00
122	5518	1	2.00	11.00	0	.00
123	5518	1	3.00	11.00	2	-.14
124	5518	1	4.00	11.00	0	.00
125	5518	1	5.00	11.00	1	.00
126	5518	1	6.00	11.00	0	.00
127	5518	1	7.00	11.00	1	-.16
128	5518	1	8.00	11.00	0	.00
129	5518	1	9.00	11.00	0	.00
130	5518	1	10.00	11.00	0	.00
131	5518	1	11.00	11.00	0	.00
132	5518	1	12.00	11.00	0	.00
133	5518	1	1.00	12.00	0	.00
134	5518	1	2.00	12.00	0	.00
135	5518	1	3.00	12.00	2	-.07
136	5518	1	4.00	12.00	0	.00
137	5518	1	5.00	12.00	1	.00
138	5518	1	6.00	12.00	0	.00
139	5518	1	7.00	12.00	1	.01
140	5518	1	8.00	12.00	0	.00

	Duration_chw	P_or_V_value_c hw	Base_value_c hw	Amplitude	onset_percen tage_chw	duration_perc entage_chw
106	.00	.00	.00	.00	.00	.00
107	.00	.00	.00	.00	.00	.00
108	.00	.00	.00	.00	.00	.00
109	.00	.00	.00	.00	.00	.00
110	.00	.00	.00	.00	.00	.00
111	.61	-12.41	13.96	1.55	-35.83	127.92
112	.00	.00	.00	.00	.00	.00
113	.48	20.54	-17.31	3.23	.02	100.00
114	.00	.00	.00	.00	.00	.00
115	.43	22.69	-19.66	3.03	2.08	88.96
116	.00	.00	.00	.00	.00	.00
117	.00	.00	.00	.00	.00	.00
118	.00	.00	.00	.00	.00	.00
119	.00	.00	.00	.00	.00	.00
120	.00	.00	.00	.00	.00	.00
121	.00	.00	.00	.00	.00	.00
122	.00	.00	.00	.00	.00	.00
123	.54	-13.45	14.47	1.02	-27.47	104.45
124	.00	.00	.00	.00	.00	.00
125	.52	20.14	-17.25	2.89	.02	100.00
126	.00	.00	.00	.00	.00	.00
127	.67	22.19	-19.73	2.46	-30.37	130.37
128	.00	.00	.00	.00	.00	.00
129	.00	.00	.00	.00	.00	.00
130	.00	.00	.00	.00	.00	.00
131	.00	.00	.00	.00	.00	.00
132	.00	.00	.00	.00	.00	.00
133	.00	.00	.00	.00	.00	.00
134	.00	.00	.00	.00	.00	.00
135	.58	-12.95	14.63	1.68	-10.91	93.97
136	.00	.00	.00	.00	.00	.00
137	.61	20.74	-17.50	3.24	.02	100.00
138	.00	.00	.00	.00	.00	.00
139	.52	23.03	-19.64	3.39	1.63	84.20
140	.00	.00	.00	.00	.00	.00

	amplitude_perc	chewing_side	Pig_code_sw	gender_sw	Pair_sw	Sw_episodes
106	.00	.00	.	.	.	.
107	.00	.00	.	.	.	.
108	.00	.00	.	.	.	.
109	.00	.00	.	.	.	.
110	.00	.00	.	.	.	.
111	-12.49	2.00	.	.	.	.
112	.00	.00	.	.	.	.
113	15.73	5.00	.	.	.	.
114	.00	.00	.	.	.	.
115	13.35	5.00	.	.	.	.
116	.00	.00	.	.	.	.
117	.00	.00	.	.	.	.
118	.00	.00	.	.	.	.
119	.00	.00	.	.	.	.
120	.00	.00	.	.	.	.
121	.00	.00	.	.	.	.
122	.00	.00	.	.	.	.
123	-7.58	2.00	.	.	.	.
124	.00	.00	.	.	.	.
125	14.35	5.00	.	.	.	.
126	.00	.00	.	.	.	.
127	11.09	5.00	.	.	.	.
128	.00	.00	.	.	.	.
129	.00	.00	.	.	.	.
130	.00	.00	.	.	.	.
131	.00	.00	.	.	.	.
132	.00	.00	.	.	.	.
133	.00	.00	.	.	.	.
134	.00	.00	.	.	.	.
135	-12.97	4.00	.	.	.	.
136	.00	.00	.	.	.	.
137	15.62	5.00	.	.	.	.
138	.00	.00	.	.	.	.
139	14.72	5.00	.	.	.	.
140	.00	.00	.	.	.	.

	P_V_sw	Onset_sw	duration_sw	P_or_V_value_sw	Base_value_sw	Range
106	.	.	.	.	.	.
107	.	.	.	.	.	.
108	.	.	.	.	.	.
109	.	.	.	.	.	.
110	.	.	.	.	.	.
111	.	.	.	.	.	.
112	.	.	.	.	.	.
113	.	.	.	.	.	.
114	.	.	.	.	.	.
115	.	.	.	.	.	.
116	.	.	.	.	.	.
117	.	.	.	.	.	.
118	.	.	.	.	.	.
119	.	.	.	.	.	.
120	.	.	.	.	.	.
121	.	.	.	.	.	.
122	.	.	.	.	.	.
123	.	.	.	.	.	.
124	.	.	.	.	.	.
125	.	.	.	.	.	.
126	.	.	.	.	.	.
127	.	.	.	.	.	.
128	.	.	.	.	.	.
129	.	.	.	.	.	.
130	.	.	.	.	.	.
131	.	.	.	.	.	.
132	.	.	.	.	.	.
133	.	.	.	.	.	.
134	.	.	.	.	.	.
135	.	.	.	.	.	.
136	.	.	.	.	.	.
137	.	.	.	.	.	.
138	.	.	.	.	.	.
139	.	.	.	.	.	.
140	.	.	.	.	.	.

	onset_percent_sw	duration_percent_sw	Range_percent	Pause_before_sw	Pause_after_sw
106	.	.	.	.	.
107	.	.	.	.	.
108	.	.	.	.	.
109	.	.	.	.	.
110	.	.	.	.	.
111	.	.	.	.	.
112	.	.	.	.	.
113	.	.	.	.	.
114	.	.	.	.	.
115	.	.	.	.	.
116	.	.	.	.	.
117	.	.	.	.	.
118	.	.	.	.	.
119	.	.	.	.	.
120	.	.	.	.	.
121	.	.	.	.	.
122	.	.	.	.	.
123	.	.	.	.	.
124	.	.	.	.	.
125	.	.	.	.	.
126	.	.	.	.	.
127	.	.	.	.	.
128	.	.	.	.	.
129	.	.	.	.	.
130	.	.	.	.	.
131	.	.	.	.	.
132	.	.	.	.	.
133	.	.	.	.	.
134	.	.	.	.	.
135	.	.	.	.	.
136	.	.	.	.	.
137	.	.	.	.	.
138	.	.	.	.	.
139	.	.	.	.	.
140	.	.	.	.	.

Sono Mastication_Raw data.sav

	Pig_chw	Gender_chw	Pair_chw	Cycles	P_V_chw	Onset_chw
141	5518	1	9.00	12.00	0	.00
142	5518	1	10.00	12.00	0	.00
143	5518	1	11.00	12.00	0	.00
144	5518	1	12.00	12.00	0	.00
145	5518	1	1.00	13.00	0	.00
146	5518	1	2.00	13.00	0	.00
147	5518	1	3.00	13.00	0	.00
148	5518	1	4.00	13.00	0	.00
149	5518	1	5.00	13.00	1	.00
150	5518	1	6.00	13.00	0	.00
151	5518	1	7.00	13.00	1	-.08
152	5518	1	8.00	13.00	0	.00
153	5518	1	9.00	13.00	0	.00
154	5518	1	10.00	13.00	0	.00
155	5518	1	11.00	13.00	0	.00
156	5518	1	12.00	13.00	0	.00
157	5518	1	1.00	17.00	0	.00
158	5518	1	2.00	17.00	0	.00
159	5518	1	3.00	17.00	2	.13
160	5518	1	4.00	17.00	0	.00
161	5518	1	5.00	17.00	1	.00
162	5518	1	6.00	17.00	0	.00
163	5518	1	7.00	17.00	1	-.01
164	5518	1	8.00	17.00	0	.00
165	5518	1	9.00	17.00	0	.00
166	5518	1	10.00	17.00	0	.00
167	5518	1	11.00	17.00	0	.00
168	5518	1	12.00	17.00	0	.00
169	5773	2	1.00	1.00	2	.13
170	5773	2	2.00	1.00	0	.00
171	5773	2	3.00	1.00	0	.00
172	5773	2	4.00	1.00	0	.00
173	5773	2	5.00	1.00	1	.00
174	5773	2	6.00	1.00	0	.00
175	5773	2	7.00	1.00	1	.01

Sono Mastication_Raw data.sav

	Duration_chw	P_or_V_value_chw	Base_value_chw	Amplitude	onset_percentage_chw	duration_percentage_chw
141	.00	.00	.00	.00	.00	.00
142	.00	.00	.00	.00	.00	.00
143	.00	.00	.00	.00	.00	.00
144	.00	.00	.00	.00	.00	.00
145	.00	.00	.00	.00	.00	.00
146	.00	.00	.00	.00	.00	.00
147	.00	.00	.00	.00	.00	.00
148	.00	.00	.00	.00	.00	.00
149	.73	20.36	-17.93	2.43	.01	100.00
150	.00	.00	.00	.00	.00	.00
151	.73	14.31	-13.52	.79	-11.28	100.96
152	.00	.00	.00	.00	.00	.00
153	.00	.00	.00	.00	.00	.00
154	.00	.00	.00	.00	.00	.00
155	.00	.00	.00	.00	.00	.00
156	.00	.00	.00	.00	.00	.00
157	.00	.00	.00	.00	.00	.00
158	.00	.00	.00	.00	.00	.00
159	.49	-12.65	13.91	1.26	19.27	73.90
160	.00	.00	.00	.00	.00	.00
161	.66	20.24	-17.54	2.70	.02	100.00
162	.00	.00	.00	.00	.00	.00
163	.63	22.38	-20.02	2.36	-1.21	95.45
164	.00	.00	.00	.00	.00	.00
165	.00	.00	.00	.00	.00	.00
166	.00	.00	.00	.00	.00	.00
167	.00	.00	.00	.00	.00	.00
168	.00	.00	.00	.00	.00	.00
169	.38	11.03	-8.37	2.66	31.37	122.15
170	.00	.00	.00	.00	.00	.00
171	.00	.00	.00	.00	.00	.00
172	.00	.00	.00	.00	.00	.00
173	.31	21.22	-18.57	2.65	.03	100.00
174	.00	.00	.00	.00	.00	.00
175	.31	27.78	-24.19	3.59	3.26	100.00

	amplitude_perc	chewing_side	Pig_code_sw	gender_sw	Pair_sw	Sw_episodes
141	.00	.00	.	.	.	.
142	.00	.00	.	.	.	.
143	.00	.00	.	.	.	.
144	.00	.00	.	.	.	.
145	.00	.00	.	.	.	.
146	.00	.00	.	.	.	.
147	.00	.00	.	.	.	.
148	.00	.00	.	.	.	.
149	11.94	5.00	.	.	.	.
150	.00	.00	.	.	.	.
151	5.52	5.00	.	.	.	.
152	.00	.00	.	.	.	.
153	.00	.00	.	.	.	.
154	.00	.00	.	.	.	.
155	.00	.00	.	.	.	.
156	.00	.00	.	.	.	.
157	.00	.00	.	.	.	.
158	.00	.00	.	.	.	.
159	-9.96	2.00	.	.	.	.
160	.00	.00	.	.	.	.
161	13.34	5.00	.	.	.	.
162	.00	.00	.	.	.	.
163	10.55	5.00	.	.	.	.
164	.00	.00	.	.	.	.
165	.00	.00	.	.	.	.
166	.00	.00	.	.	.	.
167	.00	.00	.	.	.	.
168	.00	.00	.	.	.	.
169	-24.12	2.00	.	.	.	.
170	.00	1.00	.	.	.	.
171	.00	2.00	.	.	.	.
172	.00	1.00	.	.	.	.
173	12.49	5.00	.	.	.	.
174	.00	5.00	.	.	.	.
175	12.92	5.00	.	.	.	.

Sono Mastication_Raw data.sav

	P_V_sw	Onset_sw	duration_sw	P_or_V_value_sw	Base_value_sw	Range
141	.	.	.	.	.	.
142	.	.	.	.	.	.
143	.	.	.	.	.	.
144	.	.	.	.	.	.
145	.	.	.	.	.	.
146	.	.	.	.	.	.
147	.	.	.	.	.	.
148	.	.	.	.	.	.
149	.	.	.	.	.	.
150	.	.	.	.	.	.
151	.	.	.	.	.	.
152	.	.	.	.	.	.
153	.	.	.	.	.	.
154	.	.	.	.	.	.
155	.	.	.	.	.	.
156	.	.	.	.	.	.
157	.	.	.	.	.	.
158	.	.	.	.	.	.
159	.	.	.	.	.	.
160	.	.	.	.	.	.
161	.	.	.	.	.	.
162	.	.	.	.	.	.
163	.	.	.	.	.	.
164	.	.	.	.	.	.
165	.	.	.	.	.	.
166	.	.	.	.	.	.
167	.	.	.	.	.	.
168	.	.	.	.	.	.
169	.	.	.	.	.	.
170	.	.	.	.	.	.
171	.	.	.	.	.	.
172	.	.	.	.	.	.
173	.	.	.	.	.	.
174	.	.	.	.	.	.
175	.	.	.	.	.	.

Sono Mastication_Raw data.sav

	onset_percent_sw	duration_percent_sw	Range_percent	Pause_before_sw	Pause_after_sw
141	.	.	.	.	.
142	.	.	.	.	.
143	.	.	.	.	.
144	.	.	.	.	.
145	.	.	.	.	.
146	.	.	.	.	.
147	.	.	.	.	.
148	.	.	.	.	.
149	.	.	.	.	.
150	.	.	.	.	.
151	.	.	.	.	.
152	.	.	.	.	.
153	.	.	.	.	.
154	.	.	.	.	.
155	.	.	.	.	.
156	.	.	.	.	.
157	.	.	.	.	.
158	.	.	.	.	.
159	.	.	.	.	.
160	.	.	.	.	.
161	.	.	.	.	.
162	.	.	.	.	.
163	.	.	.	.	.
164	.	.	.	.	.
165	.	.	.	.	.
166	.	.	.	.	.
167	.	.	.	.	.
168	.	.	.	.	.
169	.	.	.	.	.
170	.	.	.	.	.
171	.	.	.	.	.
172	.	.	.	.	.
173	.	.	.	.	.
174	.	.	.	.	.
175	.	.	.	.	.

	Pig_chw	Gender_chw	Pair_chw	Cycles	P_V_chw	Onset_chw
176	5773	2	8.00	1.00	0	.00
177	5773	2	9.00	1.00	0	.00
178	5773	2	10.00	1.00	2	-.04
179	5773	2	11.00	1.00	0	.00
180	5773	2	12.00	1.00	0	.00
181	5773	2	1.00	2.00	2	.08
182	5773	2	2.00	2.00	0	.00
183	5773	2	3.00	2.00	0	.00
184	5773	2	4.00	2.00	0	.00
185	5773	2	5.00	2.00	1	.00
186	5773	2	6.00	2.00	0	.00
187	5773	2	7.00	2.00	1	-.02
188	5773	2	8.00	2.00	0	.00
189	5773	2	9.00	2.00	0	.00
190	5773	2	10.00	2.00	0	.00
191	5773	2	11.00	2.00	0	.00
192	5773	2	12.00	2.00	0	.00
193	5773	2	1.00	3.00	2	.20
194	5773	2	2.00	3.00	0	.00
195	5773	2	3.00	3.00	0	.00
196	5773	2	4.00	3.00	0	.00
197	5773	2	5.00	3.00	1	.00
198	5773	2	6.00	3.00	0	.00
199	5773	2	7.00	3.00	1	-.01
200	5773	2	8.00	3.00	0	.00
201	?	?	?	?	?	?
202	?	?	?	?	?	?
203	?	?	?	?	?	?
204	?	?	?	?	?	?
205	?	?	?	?	?	?
206	?	?	?	?	?	?
207	?	?	?	?	?	?
208	?	?	?	?	?	?
209	?	?	?	?	?	?
210	?	?	?	?	?	?

	Duration_chw	P_or_V_value_chw	Base_value_chw	Amplitude	onset_percentage_chw	duration_percentage_chw
176	.00	.00	.00	.00	.00	.00
177	.00	.00	.00	.00	.00	.00
178	.32	-8.32	9.15	.83	-12.05	104.89
179	.00	.00	.00	.00	.00	.00
180	.00	.00	.00	.00	.00	.00
181	.52	10.40	-7.70	2.70	23.58	146.88
182	.00	.00	.00	.00	.00	.00
183	.00	.00	.00	.00	.00	.00
184	.00	.00	.00	.00	.00	.00
185	.35	21.96	-18.18	3.78	.03	100.00
186	.00	.00	.00	.00	.00	.00
187	.36	28.38	-23.06	5.32	-4.26	102.27
188	.00	.00	.00	.00	.00	.00
189	.00	.00	.00	.00	.00	.00
190	.00	.00	.00	.00	.00	.00
191	.00	.00	.00	.00	.00	.00
192	.00	.00	.00	.00	.00	.00
193	.41	10.53	-8.13	2.40	29.27	98.30
194	.00	.00	.00	.00	.00	.00
195	.00	.00	.00	.00	.00	.00
196	.00	.00	.00	.00	.00	.00
197	.41	21.73	-19.50	2.23	.02	100.00
198	.00	.00	.00	.00	.00	.00
199	.44	27.85	-24.22	3.63	-1.70	107.28
200	.00	.00	.00	.00	.00	.00
201	.00	.00	.00	.00	?	?
202	.00	.00	.00	.00	?	?
203	.00	.00	.00	.00	?	?
204	.00	.00	.00	.00	?	?
205	.43	10.41	-7.34	3.07	?	?
206	.00	.00	.00	.00	?	?
207	.00	.00	.00	.00	?	?
208	.00	.00	.00	.00	?	?
209	.38	23.33	-18.58	4.75	?	?
210	.00	.00	.00	.00	?	?

	amplitude_perc	chewing_side	Pig_code_sw	gender_sw	Pair_sw	Sw_episodes
176	.00	5.00	.	.	.	.
177	.00	2.00	.	.	.	.
178	-9.98	1.00	.	.	.	.
179	.00	2.00	.	.	.	.
180	.00	1.00	.	.	.	.
181	-25.96	2.00	.	.	.	.
182	.00	1.00	.	.	.	.
183	.00	2.00	.	.	.	.
184	.00	1.00	.	.	.	.
185	17.21	5.00	.	.	.	.
186	.00	5.00	.	.	.	.
187	18.75	5.00	.	.	.	.
188	.00	5.00	.	.	.	.
189	.00	2.00	.	.	.	.
190	.00	1.00	.	.	.	.
191	.00	2.00	.	.	.	.
192	.00	1.00	.	.	.	.
193	-22.79	2.00	.	.	.	.
194	.00	1.00	.	.	.	.
195	.00	2.00	.	.	.	.
196	.00	1.00	.	.	.	.
197	10.26	5.00	.	.	.	.
198	.00	5.00	.	.	.	.
199	13.03	5.00	.	.	.	.
200	.00	5.00	.	.	.	.
201	.00	2.00	.	.	.	.
202	.00	1.00	.	.	.	.
203	.00	2.00	.	.	.	.
204	.00	1.00	.	.	.	.
205	-29.49	2.00	.	.	.	.
206	.00	1.00	.	.	.	.
207	.00	2.00	.	.	.	.
208	.00	1.00	.	.	.	.
209	20.36	5.00	.	.	.	.
210	.00	5.00	.	.	.	.

Sono Mastication_Raw data.sav

	P_V_sw	Onset_sw	duration_sw	P_or_V_value_sw	Base_value_sw	Range
176	.	.	.	.	.	.
177	.	.	.	.	.	.
178	.	.	.	.	.	.
179	.	.	.	.	.	.
180	.	.	.	.	.	.
181	.	.	?	?	.	.
182	.	.	.	.	.	.
183	.	.	.	.	.	.
184	.	.	.	.	.	.
185	.	.	.	.	.	.
186	.	.	.	.	.	.
187	.	.	.	.	.	.
188	.	.	.	.	.	.
189	.	.	.	.	.	.
190	.	.	.	.	.	.
191	.	.	.	.	.	.
192	.	.	.	.	.	.
193	.	.	.	.	.	.
194	.	.	.	.	.	.
195	.	.	.	.	.	.
196	.	.	.	.	.	.
197	.	.	.	.	.	.
198	.	.	.	.	.	.
199	.	.	.	.	.	.
200	.	.	.	.	.	.
201	.	.	?	?	?	?
202	.	.	?	?	?	?
203	.	.	?	?	?	?
204	.	.	?	?	?	?
205	.	.	?	?	?	?
206	.	.	?	?	?	?
207	.	.	?	?	?	?
208	.	.	?	?	?	?
209	.	.	?	?	?	?
210	.	.	?	?	?	?

	onset_percent_sw	duration_percent_sw	Range_percent	Pause_before_sw	Pause_after_sw
176	.	.	.	.	.
177	.	.	.	.	.
178	.	.	.	.	.
179	.	.	.	.	.
180	.	.	.	.	.
181	.	.	.	.	.
182	.	.	.	.	.
183	.	.	.	.	.
184	.	.	.	.	.
185	.	.	.	.	.
186	.	.	.	.	.
187	.	.	.	.	.
188	.	.	.	.	.
189	.	.	.	.	.
190	.	.	.	.	.
191	.	.	.	.	.
192	.	.	.	.	.
193	.	.	.	.	.
194	.	.	.	.	.
195	.	.	.	.	.
196	.	.	.	.	.
197	.	.	.	.	.
198	.	.	.	.	.
199	.	.	.	.	.
200	.	.	.	.	.
201	.	.	.	.	.
202	.	.	.	.	.
203	.	.	.	.	.
204	.	.	.	.	.
205	.	.	.	.	.
206	.	.	.	.	.
207	.	.	.	.	.
208	.	.	.	.	.
209	.	.	.	.	.
210	.	.	.	.	.

Sono Mastication_Raw data.sav

	Pig_chw	Gender_chw	Pair_chw	Cycles	P_V_chw	Onset_chw
211	5773	2	7.00	8.00	1	.01
212	5773	2	8.00	8.00	0	.00
213	5773	2	9.00	8.00	0	.00
214	5773	2	10.00	8.00	0	.00
215	5773	2	11.00	8.00	0	.00
216	5773	2	12.00	8.00	0	.00
217	5773	2	1.00	9.00	2	-.27
218	5773	2	2.00	9.00	0	.00
219	5773	2	3.00	9.00	0	.00
220	5773	2	4.00	9.00	0	.00
221	5773	2	5.00	9.00	1	.00
222	5773	2	6.00	9.00	0	.00
223	5773	2	7.00	9.00	1	.01
224	5773	2	8.00	9.00	0	.00
225	5773	2	9.00	9.00	0	.00
226	5773	2	10.00	9.00	0	.00
227	5773	2	11.00	9.00	0	.00
228	5773	2	12.00	9.00	0	.00
229	5773	2	1.00	10.00	2	-.26
230	5773	2	2.00	10.00	0	.00
231	5773	2	3.00	10.00	0	.00
232	5773	2	4.00	10.00	0	.00
233	5773	2	5.00	10.00	1	.00
234	5773	2	6.00	10.00	0	.00
235	5773	2	7.00	10.00	1	.05
236	5773	2	8.00	10.00	0	.00
237	5773	2	9.00	10.00	2	-.19
238	5773	2	10.00	10.00	0	.00
239	5773	2	11.00	10.00	0	.00
240	5773	2	12.00	10.00	0	.00
241	?	?	?	?	?	?
242	?	?	?	?	?	?
243	?	?	?	?	?	?
244	?	?	?	?	?	?
245	?	?	?	?	?	?

	Duration_chw	P_or_V_value_chw	Base_value_chw	Amplitude	onset_percentage_chw	duration_percentage_chw
211	.51	28.48	-23.07	5.41	2.67	136.00
212	.00	.00	.00	.00	.00	.00
213	.00	.00	.00	.00	.00	.00
214	.00	.00	.00	.00	.00	.00
215	.00	.00	.00	.00	.00	.00
216	.00	.00	.00	.00	.00	.00
217	.44	-7.22	10.41	3.19	-35.24	89.32
218	.00	.00	.00	.00	.00	.00
219	.00	.00	.00	.00	.00	.00
220	.00	.00	.00	.00	.00	.00
221	.49	23.17	-18.60	4.57	.02	100.00
222	.00	.00	.00	.00	.00	.00
223	.49	28.46	-22.98	5.48	1.64	100.00
224	.00	.00	.00	.00	.00	.00
225	.00	.00	.00	.00	.00	.00
226	.00	.00	.00	.00	.00	.00
227	.00	.00	.00	.00	.00	.00
228	.00	.00	.00	.00	.00	.00
229	.39	-6.86	10.50	3.64	-32.19	77.69
230	.00	.00	.00	.00	.00	.00
231	.00	.00	.00	.00	.00	.00
232	.00	.00	.00	.00	.00	.00
233	.50	23.91	-18.72	5.19	.02	100.00
234	.00	.00	.00	.00	.00	.00
235	.46	28.84	-22.85	5.99	8.96	91.04
236	.00	.00	.00	.00	.00	.00
237	.50	-5.96	6.66	.70	.00	98.61
238	.00	.00	.00	.00	.00	.00
239	.00	.00	.00	.00	.00	.00
240	.00	.00	.00	.00	.00	.00
241	.46	-7.27	10.52	3.25	?	?
242	.00	.00	.00	.00	?	?
243	.00	.00	.00	.00	?	?
244	.00	.00	.00	.00	?	?
245	.45	24.14	-19.16	4.98	?	?

Sono Mastication_Raw data.sav

	amplitude_perc	chewing_side	Pig_code_sw	gender_sw	Pair_sw	Sw_episodes
211	19.00	5.00	.	.	.	.
212	.00	5.00	.	.	.	.
213	.00	2.00	.	.	.	.
214	.00	1.00	.	.	.	.
215	.00	2.00	.	.	.	.
216	.00	1.00	.	.	.	.
217	-44.18	4.00	.	.	.	.
218	.00	3.00	.	.	.	.
219	.00	4.00	.	.	.	.
220	.00	3.00	.	.	.	.
221	19.72	5.00	.	.	.	.
222	.00	5.00	.	.	.	.
223	19.26	5.00	.	.	.	.
224	.00	5.00	.	.	.	.
225	.00	4.00	.	.	.	.
226	.00	3.00	.	.	.	.
227	.00	4.00	.	.	.	.
228	.00	3.00	.	.	.	.
229	-53.06	4.00	.	.	.	.
230	.00	3.00	.	.	.	.
231	.00	4.00	.	.	.	.
232	.00	3.00	.	.	.	.
233	21.71	5.00	.	.	.	.
234	.00	5.00	.	.	.	.
235	20.77	5.00	.	.	.	.
236	.00	5.00	.	.	.	.
237	-11.75	4.00	.	.	.	.
238	.00	3.00	.	.	.	.
239	.00	4.00	.	.	.	.
240	.00	3.00	.	.	.	.
241	-44.70	4.00	.	.	.	.
242	.00	3.00	.	.	.	.
243	.00	4.00	.	.	.	.
244	.00	3.00	.	.	.	.
245	20.63	5.00	.	.	.	.

	P_V_sw	Onset_sw	duration_sw	P_or_V_value_sw	Base_value_sw	Range
211	.	.	.	.	.	.
212	.	.	.	.	.	.
213	.	.	.	.	.	.
214	.	.	.	.	.	.
215	.	.	.	.	.	.
216	.	.	.	.	.	.
217	.	.	.	.	.	.
218	.	.	.	.	.	.
219	.	.	.	.	.	.
220	.	.	.	.	.	.
221	.	.	.	.	.	.
222	.	.	.	.	.	.
223	.	.	.	.	.	.
224	.	.	.	.	.	.
225	.	.	.	.	.	.
226	.	.	.	.	.	.
227	.	.	.	.	.	.
228	.	.	.	.	.	.
229	.	.	.	.	.	.
230	.	.	.	.	.	.
231	.	.	.	.	.	.
232	.	.	.	.	.	.
233	.	.	.	.	.	.
234	.	.	.	.	.	.
235	.	.	.	.	.	.
236	.	.	.	.	.	.
237	.	.	.	.	.	.
238	.	.	.	.	.	.
239	.	.	.	.	.	.
240	.	.	.	.	.	.
241	.	.	?	?	?	?
242	.	.	?	?	?	?
243	.	.	?	?	?	?
244	.	.	?	?	?	?
245	.	.	?	?	?	?

	onset_percent_sw	duration_percent_sw	Range_percent	Pause_before_sw	Pause_after_sw
211	.	.	.	.	.
212	.	.	.	.	.
213	.	.	.	.	.
214	.	.	.	.	.
215	.	.	.	.	.
216	.	.	.	.	.
217	.	.	.	.	.
218	.	.	.	.	.
219	.	.	.	.	.
220	.	.	.	.	.
221	.	.	.	.	.
222	.	.	.	.	.
223	.	.	.	.	.
224	.	.	.	.	.
225	.	.	.	.	.
226	.	.	.	.	.
227	.	.	.	.	.
228	.	.	.	.	.
229	.	.	.	.	.
230	.	.	.	.	.
231	.	.	.	.	.
232	.	.	.	.	.
233	.	.	.	.	.
234	.	.	.	.	.
235	.	.	.	.	.
236	.	.	.	.	.
237	.	.	.	.	.
238	.	.	.	.	.
239	.	.	.	.	.
240	.	.	.	.	.
241	.	.	.	.	.
242	.	.	.	.	.
243	.	.	.	.	.
244	.	.	.	.	.
245	.	.	.	.	.

	Pig_chw	Gender_chw	Pair_chw	Cycles	P_V_chw	Onset_chw
246	5773	2	6.00	11.00	0	.00
247	5773	2	7.00	11.00	1	-.02
248	5773	2	8.00	11.00	0	.00
249	5773	2	9.00	11.00	0	.00
250	5773	2	10.00	11.00	0	.00
251	5773	2	11.00	11.00	0	.00
252	5773	2	12.00	11.00	0	.00
253	5773	2	1.00	12.00	2	-.28
254	5773	2	2.00	12.00	0	.00
255	5773	2	3.00	12.00	0	.00
256	5773	2	4.00	12.00	0	.00
257	5773	2	5.00	12.00	1	.00
258	5773	2	6.00	12.00	0	.00
259	5773	2	7.00	12.00	1	.01
260	5773	2	8.00	12.00	0	.00
261	5773	2	9.00	12.00	0	.00
262	5773	2	10.00	12.00	0	.00
263	5773	2	11.00	12.00	0	.00
264	5773	2	12.00	12.00	0	.00
265	5773	2	1.00	13.00	2	-.35
266	5773	2	2.00	13.00	0	.00
267	5773	2	3.00	13.00	0	.00
268	5773	2	4.00	13.00	0	.00
269	5773	2	5.00	13.00	1	.00
270	5773	2	6.00	13.00	0	.00
271	5773	2	7.00	13.00	1	.01
272	5773	2	8.00	13.00	0	.00
273	5773	2	9.00	13.00	0	.00
274	5773	2	10.00	13.00	0	.00
275	5773	2	11.00	13.00	0	.00
276	5773	2	12.00	13.00	0	.00
277	5773	2	1.00	14.00	2	-.40
278	5773	2	2.00	14.00	0	.00
279	5773	2	3.00	14.00	0	.00
280	5773	2	4.00	14.00	0	.00

	Duration_chw	P_or_V_value_c hw	Base_value_c hw	Amplitude	onset_percen tage_chw	duration_perc entage_chw
246	.00	.00	.00	.00	.00	.00
247	.45	29.73	-23.21	6.52	-4.89	100.00
248	.00	.00	.00	.00	.00	.00
249	.00	.00	.00	.00	.00	.00
250	.00	.00	.00	.00	.00	.00
251	.00	.00	.00	.00	.00	.00
252	.00	.00	.00	.00	.00	.00
253	.47	-7.47	11.48	4.01	-32.07	87.41
254	.00	.00	.00	.00	.00	.00
255	.00	.00	.00	.00	.00	.00
256	.00	.00	.00	.00	.00	.00
257	.53	24.08	-19.64	4.44	.02	100.00
258	.00	.00	.00	.00	.00	.00
259	.56	29.57	-23.92	5.65	1.88	104.32
260	.00	.00	.00	.00	.00	.00
261	.00	.00	.00	.00	.00	.00
262	.00	.00	.00	.00	.00	.00
263	.00	.00	.00	.00	.00	.00
264	.00	.00	.00	.00	.00	.00
265	.50	-7.77	10.93	3.16	.00	94.29
266	.00	.00	.00	.00	.00	.00
267	.00	.00	.00	.00	.00	.00
268	.00	.00	.00	.00	.00	.00
269	.53	24.83	-19.73	5.10	.02	100.00
270	.00	.00	.00	.00	.00	.00
271	.50	30.03	-23.63	6.40	1.33	94.29
272	.00	.00	.00	.00	.00	.00
273	.00	.00	.00	.00	.00	.00
274	.00	.00	.00	.00	.00	.00
275	.00	.00	.00	.00	.00	.00
276	.00	.00	.00	.00	.00	.00
277	.49	-7.77	10.96	3.19	-34.52	115.95
278	.00	.00	.00	.00	.00	.00
279	.00	.00	.00	.00	.00	.00
280	.00	.00	.00	.00	.00	.00

	amplitude_perc	chewing_side	Pig_code_sw	gender_sw	Pair_sw	Sw_episodes
246	.00	5.00	.	.	.	.
247	21.93	5.00	.	.	.	.
248	.00	5.00	.	.	.	.
249	.00	4.00	.	.	.	.
250	.00	3.00	.	.	.	.
251	.00	4.00	.	.	.	.
252	.00	3.00	.	.	.	.
253	-53.68	4.00	.	.	.	.
254	.00	3.00	.	.	.	.
255	.00	4.00	.	.	.	.
256	.00	3.00	.	.	.	.
257	18.44	5.00	.	.	.	.
258	.00	5.00	.	.	.	.
259	19.11	5.00	.	.	.	.
260	.00	5.00	.	.	.	.
261	.00	4.00	.	.	.	.
262	.00	3.00	.	.	.	.
263	.00	4.00	.	.	.	.
264	.00	3.00	.	.	.	.
265	-40.67	2.00	.	.	.	.
266	.00	1.00	.	.	.	.
267	.00	2.00	.	.	.	.
268	.00	1.00	.	.	.	.
269	20.54	5.00	.	.	.	.
270	.00	5.00	.	.	.	.
271	21.31	5.00	.	.	.	.
272	.00	5.00	.	.	.	.
273	.00	2.00	.	.	.	.
274	.00	1.00	.	.	.	.
275	.00	2.00	.	.	.	.
276	.00	1.00	.	.	.	.
277	-41.06	2.00	.	.	.	.
278	.00	1.00	.	.	.	.
279	.00	2.00	.	.	.	.
280	.00	1.00	.	.	.	.

	P_V_sw	Onset_sw	duration_sw	P_or_V_value_sw	Base_value_sw	Range
246	.	.	.	.	.	.
247	.	.	.	.	.	.
248	.	.	.	.	.	.
249	.	.	.	.	.	.
250	.	.	.	.	.	.
251	.	.	.	.	.	.
252	.	.	.	.	.	.
253	.	.	.	.	.	.
254	.	.	.	.	.	.
255	.	.	.	.	.	.
256	.	.	.	.	.	.
257	.	.	.	.	.	.
258	.	.	.	.	.	.
259	.	.	.	.	.	.
260	.	.	.	.	.	.
261	.	.	.	.	.	.
262	.	.	.	.	.	.
263	.	.	.	.	.	.
264	.	.	.	.	.	.
265	.	.	.	.	.	.
266	.	.	.	.	.	.
267	.	.	.	.	.	.
268	.	.	.	.	.	.
269	.	.	.	.	.	.
270	.	.	.	.	.	.
271	.	.	.	.	.	.
272	.	.	.	.	.	.
273	.	.	.	.	.	.
274	.	.	.	.	.	.
275	.	.	.	.	.	.
276	.	.	.	.	.	.
277	.	.	.	.	.	.
278	.	.	.	.	.	.
279	.	.	.	.	.	.
280	.	.	.	.	.	.

	onset_percent_sw	duration_percent_sw	Range_percent	Pause_before_sw	Pause_after_sw
246	.	.	.	.	.
247	.	.	.	.	.
248	.	.	.	.	.
249	.	.	.	.	.
250	.	.	.	.	.
251	.	.	.	.	.
252	.	.	.	.	.
253	.	.	.	.	.
254	.	.	.	.	.
255	.	.	.	.	.
256	.	.	.	.	.
257	.	.	.	.	.
258	.	.	.	.	.
259	.	.	.	.	.
260	.	.	.	.	.
261	.	.	.	.	.
262	.	.	.	.	.
263	.	.	.	.	.
264	.	.	.	.	.
265	.	.	.	.	.
266	.	.	.	.	.
267	.	.	.	.	.
268	.	.	.	.	.
269	.	.	.	.	.
270	.	.	.	.	.
271	.	.	.	.	.
272	.	.	.	.	.
273	.	.	.	.	.
274	.	.	.	.	.
275	.	.	.	.	.
276	.	.	.	.	.
277	.	.	.	.	.
278	.	.	.	.	.
279	.	.	.	.	.
280	.	.	.	.	.

	Pig_chw	Gender_chw	Pair_chw	Cycles	P_V_chw	Onset_chw
281	5773	2	5.00	14.00	1	.00
282	5773	2	6.00	14.00	0	.00
283	5773	2	7.00	14.00	1	.01
284	5773	2	8.00	14.00	0	.00
285	5773	2	9.00	14.00	2	-.30
286	5773	2	10.00	14.00	0	.00
287	5773	2	11.00	14.00	0	.00
288	5773	2	12.00	14.00	0	.00
289	5773	2	1.00	15.00	2	-.41
290	5773	2	2.00	15.00	0	.00
291	5773	2	3.00	15.00	0	.00
292	5773	2	4.00	15.00	0	.00
293	5773	2	5.00	15.00	1	.00
294	5773	2	6.00	15.00	0	.00
295	5773	2	7.00	15.00	1	-.02
296	5773	2	8.00	15.00	0	.00
297	5773	2	9.00	15.00	0	.00
298	5773	2	10.00	15.00	0	.00
299	5773	2	11.00	15.00	0	.00
300	5773	2	12.00	15.00	0	.00
301	5794	2	1.00	1.00	0	.00
302	5794	2	2.00	1.00	0	.00
303	5794	2	3.00	1.00	0	.00
304	5794	2	4.00	1.00	0	.00
305	5794	2	5.00	1.00	1	.00
306	5794	2	6.00	1.00	0	.00
307	5794	2	7.00	1.00	1	-.01
308	5794	2	8.00	1.00	1	.08
309	5794	2	9.00	1.00	0	.00
310	5794	2	10.00	1.00	2	-.22
311	5794	2	11.00	1.00	0	.00
312	5794	2	12.00	1.00	0	.00
313	5794	2	1.00	2.00	0	.00
314	5794	2	2.00	2.00	0	.00
315	5794	2	3.00	2.00	0	.00

	Duration_chw	P_or_V_value_chw	Base_value_chw	Amplitude	onset_percentage_chw	duration_percentage_chw
281	.42	24.70	-19.85	4.85	.02	100.00
282	.00	.00	.00	.00	.00	.00
283	.41	30.71	-23.75	6.96	2.38	98.10
284	.00	.00	.00	.00	.00	.00
285	.50	-5.72	6.43	.71	-31.43	119.52
286	.00	.00	.00	.00	.00	.00
287	.00	.00	.00	.00	.00	.00
288	.00	.00	.00	.00	.00	.00
289	.51	-7.67	11.25	3.58	.00	86.15
290	.00	.00	.00	.00	.00	.00
291	.00	.00	.00	.00	.00	.00
292	.00	.00	.00	.00	.00	.00
293	.59	24.45	-19.75	4.70	.02	100.00
294	.00	.00	.00	.00	.00	.00
295	.69	30.93	-23.67	7.26	-3.72	116.39
296	.00	.00	.00	.00	.00	.00
297	.00	.00	.00	.00	.00	.00
298	.00	.00	.00	.00	.00	.00
299	.00	.00	.00	.00	.00	.00
300	.00	.00	.00	.00	.00	.00
301	.00	.00	.00	.00	.00	.00
302	.00	.00	.00	.00	.00	.00
303	.00	.00	.00	.00	.00	.00
304	.00	.00	.00	.00	.00	.00
305	.88	18.43	-14.15	4.28	.11	100.00
306	.00	.00	.00	.00	.00	.00
307	.87	22.66	-18.05	4.61	-.79	98.30
308	.71	22.34	-20.35	1.99	9.39	80.54
309	.00	.00	.00	.00	.00	.00
310	.91	-12.31	14.73	2.42	-24.55	102.60
311	.00	.00	.00	.00	.00	.00
312	.00	.00	.00	.00	.00	.00
313	.00	.00	.00	.00	.00	.00
314	.00	.00	.00	.00	.00	.00
315	.00	.00	.00	.00	.00	.00

Sono Mastication_Raw data.sav

	amplitude_perc	chewing_side	Pig_code_sw	gender_sw	Pair_sw	Sw_episodes
281	19.64	5.00	.	.	.	.
282	.00	5.00	.	.	.	.
283	22.66	5.00	.	.	.	.
284	.00	5.00	.	.	.	.
285	-12.41	2.00	.	.	.	.
286	.00	1.00	.	.	.	.
287	.00	2.00	.	.	.	.
288	.00	1.00	.	.	.	.
289	-46.68	4.00	.	.	.	.
290	.00	3.00	.	.	.	.
291	.00	4.00	.	.	.	.
292	.00	3.00	.	.	.	.
293	19.22	5.00	.	.	.	.
294	.00	5.00	.	.	.	.
295	23.47	5.00	.	.	.	.
296	.00	5.00	.	.	.	.
297	.00	4.00	.	.	.	.
298	.00	3.00	.	.	.	.
299	.00	4.00	.	.	.	.
300	.00	.00	.	.	.	.
301	.00	4.00	.	.	.	.
302	.00	3.00	.	.	.	.
303	.00	4.00	.	.	.	.
304	.00	3.00	.	.	.	.
305	23.22	5.00	.	.	.	.
306	.00	5.00	.	.	.	.
307	20.34	5.00	.	.	.	.
308	8.91	5.00	.	.	.	.
309	.00	4.00	.	.	.	.
310	-19.66	3.00	.	.	.	.
311	.00	4.00	.	.	.	.
312	.00	3.00	.	.	.	.
313	.00	4.00	.	.	.	.
314	.00	3.00	.	.	.	.
315	.00	4.00	.	.	.	.

	P_V_sw	Onset_sw	duration_sw	P_or_V_value_sw	Base_value_sw	Range
281	.	.	.	.	.	.
282	.	.	.	.	.	.
283	.	.	.	.	.	.
284	.	.	.	.	.	.
285	.	.	.	.	.	.
286	.	.	.	.	.	.
287	.	.	.	.	.	.
288	.	.	.	.	.	.
289	.	.	.	.	.	.
290	.	.	.	.	.	.
291	.	.	.	.	.	.
292	.	.	.	.	.	.
293	.	.	.	.	.	.
294	.	.	.	.	.	.
295	.	.	.	.	.	.
296	.	.	.	.	.	.
297	.	.	.	.	.	.
298	.	.	.	.	.	.
299	.	.	.	.	.	.
300	.	.	.	.	.	.
301	.	.	.	.	.	.
302	.	.	.	.	.	.
303	.	.	.	.	.	.
304	.	.	.	.	.	.
305	.	.	.	.	.	.
306	.	.	.	.	.	.
307	.	.	.	.	.	.
308	.	.	.	.	.	.
309	.	.	.	.	.	.
310	.	.	.	.	.	.
311	.	.	.	.	.	.
312	.	.	.	.	.	.
313	.	.	.	.	.	.
314	.	.	.	.	.	.
315	.	.	.	.	.	.

	onset_percent_sw	duration_percent_sw	Range_percent	Pause_before_sw	Pause_after_sw
281	.	.	.	.	.
282	.	.	.	.	.
283	.	.	.	.	.
284	.	.	.	.	.
285	.	.	.	.	.
286	.	.	.	.	.
287	.	.	.	.	.
288	.	.	.	.	.
289	.	.	.	.	.
290	.	.	.	.	.
291	.	.	.	.	.
292	.	.	.	.	.
293	.	.	.	.	.
294	.	.	.	.	.
295	.	.	.	.	.
296	.	.	.	.	.
297	.	.	.	.	.
298	.	.	.	.	.
299	.	.	.	.	.
300	.	.	.	.	.
301	.	.	.	.	.
302	.	.	.	.	.
303	.	.	.	.	.
304	.	.	.	.	.
305	.	.	.	.	.
306	.	.	.	.	.
307	.	.	.	.	.
308	.	.	.	.	.
309	.	.	.	.	.
310	.	.	.	.	.
311	.	.	.	.	.
312	.	.	.	.	.
313	.	.	.	.	.
314	.	.	.	.	.
315	.	.	.	.	.

Sono Mastication_Raw data.sav

	Pig_chw	Gender_chw	Pair_chw	Cycles	P_V_chw	Onset_chw
316	5794	2	4.00	2.00	0	.00
317	5794	2	5.00	2.00	1	.00
318	5794	2	6.00	2.00	0	.00
319	5794	2	7.00	2.00	1	-.01
320	5794	2	8.00	2.00	1	.09
321	5794	2	9.00	2.00	0	.00
322	5794	2	10.00	2.00	2	-.18
323	5794	2	11.00	2.00	0	.00
324	5794	2	12.00	2.00	0	.00
325	5794	2	1.00	3.00	0	.00
326	5794	2	2.00	3.00	0	.00
327	5794	2	3.00	3.00	0	.00
328	5794	2	4.00	3.00	0	.00
329	5794	2	5.00	3.00	1	.00
330	5794	2	6.00	3.00	0	.00
331	5794	2	7.00	3.00	1	-.01
332	5794	2	8.00	3.00	1	.11
333	5794	2	9.00	3.00	0	.00
334	5794	2	10.00	3.00	2	-.20
335	5794	2	11.00	3.00	0	.00
336	5794	2	12.00	3.00	0	.00
337	5794	2	1.00	4.00	0	.00
338	5794	2	2.00	4.00	0	.00
339	5794	2	3.00	4.00	0	.00
340	5794	2	4.00	4.00	0	.00
341	?	?	?	?	?	?
342	?	?	?	?	?	?
343	?	?	?	?	?	?
344	?	?	?	?	?	?
345	?	?	?	?	?	?
346	?	?	?	?	?	?
347	?	?	?	?	?	?
348	?	?	?	?	?	?
349	?	?	?	?	?	?
350	?	?	?	?	?	?

	Duration_chw	P_or_V_value_chw	Base_value_chw	Amplitude	onset_percentage_chw	duration_percentage_chw
316	.00	.00	.00	.00	.00	.00
317	.76	18.57	-14.24	4.33	.13	100.00
318	.00	.00	.00	.00	.00	.00
319	.74	23.00	-18.10	4.90	-1.06	98.02
320	.58	22.30	-20.40	1.90	11.89	77.15
321	.00	.00	.00	.00	.00	.00
322	.73	-12.29	14.55	2.26	-23.78	96.96
323	.00	.00	.00	.00	.00	.00
324	.00	.00	.00	.00	.00	.00
325	.00	.00	.00	.00	.00	.00
326	.00	.00	.00	.00	.00	.00
327	.00	.00	.00	.00	.00	.00
328	.00	.00	.00	.00	.00	.00
329	.69	18.72	-14.85	3.87	.15	100.00
330	.00	.00	.00	.00	.00	.00
331	.60	23.25	-18.23	5.02	-1.02	86.94
332	.48	22.48	-20.53	1.95	15.24	69.67
333	.00	.00	.00	.00	.00	.00
334	.62	-12.95	15.20	2.25	-28.30	90.28
335	.00	.00	.00	.00	.00	.00
336	.00	.00	.00	.00	.00	.00
337	.00	.00	.00	.00	.00	.00
338	.00	.00	.00	.00	.00	.00
339	.00	.00	.00	.00	.00	.00
340	.00	.00	.00	.00	.00	.00
341	.80	18.77	-14.36	4.41	?	?
342	.00	.00	.00	.00	?	?
343	.86	22.96	-18.15	4.81	?	?
344	.61	22.28	-19.97	2.31	?	?
345	.00	.00	.00	.00	?	?
346	.84	-12.62	15.20	2.58	?	?
347	.00	.00	.00	.00	?	?
348	.00	.00	.00	.00	?	?
349	.00	.00	.00	.00	?	?
350	.00	.00	.00	.00	?	?

Sono Mastication_Raw data.sav

	amplitude_perc	chewing_side	Pig_code_sw	gender_sw	Pair_sw	Sw_episodes
316	.00	3.00	.	.	.	.
317	23.32	5.00	.	.	.	.
318	.00	5.00	.	.	.	.
319	21.30	5.00	.	.	.	.
320	8.52	5.00	.	.	.	.
321	.00	4.00	.	.	.	.
322	-18.39	3.00	.	.	.	.
323	.00	4.00	.	.	.	.
324	.00	3.00	.	.	.	.
325	.00	4.00	.	.	.	.
326	.00	3.00	.	.	.	.
327	.00	4.00	.	.	.	.
328	.00	3.00	.	.	.	.
329	20.67	5.00	.	.	.	.
330	.00	5.00	.	.	.	.
331	21.59	5.00	.	.	.	.
332	8.67	5.00	.	.	.	.
333	.00	4.00	.	.	.	.
334	-17.38	3.00	.	.	.	.
335	.00	4.00	.	.	.	.
336	.00	3.00	.	.	.	.
337	.00	4.00	.	.	.	.
338	.00	3.00	.	.	.	.
339	.00	4.00	.	.	.	.
340	.00	3.00	.	.	.	.
341	23.50	5.00	.	.	.	.
342	.00	5.00	.	.	.	.
343	20.95	5.00	.	.	.	.
344	10.37	5.00	.	.	.	.
345	.00	4.00	.	.	.	.
346	-20.44	3.00	.	.	.	.
347	.00	4.00	.	.	.	.
348	.00	3.00	.	.	.	.
349	.00	2.00	.	.	.	.
350	.00	1.00	.	.	.	.

	P_V_sw	Onset_sw	duration_sw	P_or_V_value_sw	Base_value_sw	Range
316	.	.	.	.	.	.
317	.	.	.	.	.	.
318	.	.	.	.	.	.
319	.	.	.	.	.	.
320	.	.	.	.	.	.
321	.	.	?	?	?	?
322	.	.	?	?	?	?
323	.	.	?	?	?	?
324	.	.	?	?	?	?
325	.	.	?	?	?	?
326	.	.	?	.	.	.
327	.	.	.	.	.	.
328	.	.	.	.	.	.
329	.	.	.	.	.	.
330	.	.	.	.	.	.
331	.	.	.	.	.	.
332	.	.	.	.	.	.
333	.	.	.	.	.	.
334	.	.	.	.	.	.
335	.	.	.	.	.	.
336	.	.	.	.	.	.
337	.	.	.	.	.	.
338	.	.	.	.	.	.
339	.	.	.	.	.	.
340	.	.	.	.	.	.
341	.	.	?	?	?	?
342	.	.	?	?	?	?
343	.	.	?	?	?	?
344	.	.	?	?	?	?
345	.	.	?	?	?	?
346	.	.	?	?	?	?
347	.	.	?	?	?	?
348	.	.	?	?	?	?
349	.	.	?	?	?	?
350	.	.	?	?	?	?

Sono Mastication_Raw data.sav

	onset_percent_sw	duration_percent_sw	Range_percent	Pause_before_sw	Pause_after_sw
316	.	.	.	.	.
317	.	.	.	.	.
318	.	.	.	.	.
319	.	.	.	.	.
320	.	.	.	.	.
321	.	.	.	.	.
322	.	.	.	.	.
323	.	.	.	.	.
324	.	.	.	.	.
325	.	.	.	.	.
326	.	.	.	.	.
327	.	.	.	.	.
328	.	.	.	.	.
329	.	.	.	.	.
330	.	.	.	.	.
331	.	.	.	.	.
332	.	.	.	.	.
333	.	.	.	.	.
334	.	.	.	.	.
335	.	.	.	.	.
336	.	.	.	.	.
337	.	.	.	.	.
338	.	.	.	.	.
339	.	.	.	.	.
340	.	.	.	.	.
341	.	.	.	.	.
342	.	.	.	.	.
343	.	.	.	.	.
344	.	.	.	.	.
345	.	.	.	.	.
346	.	.	.	.	.
347	.	.	.	.	.
348	.	.	.	.	.
349	.	.	.	.	.
350	.	.	.	.	.

Sono Mastication_Raw data.sav

	Pig_chw	Gender_chw	Pair_chw	Cycles	P_V_chw	Onset_chw
351	5794	2	3.00	5.00	0	.00
352	5794	2	4.00	5.00	0	.00
353	5794	2	5.00	5.00	1	.00
354	5794	2	6.00	5.00	0	.00
355	5794	2	7.00	5.00	1	-.02
356	5794	2	8.00	5.00	1	.07
357	5794	2	9.00	5.00	0	.00
358	5794	2	10.00	5.00	2	-.22
359	5794	2	11.00	5.00	0	.00
360	5794	2	12.00	5.00	0	.00
361	5794	2	1.00	6.00	0	.00
362	5794	2	2.00	6.00	0	.00
363	5794	2	3.00	6.00	0	.00
364	5794	2	4.00	6.00	0	.00
365	5794	2	5.00	6.00	1	.00
366	5794	2	6.00	6.00	0	.00
367	5794	2	7.00	6.00	1	-.01
368	5794	2	8.00	6.00	1	.08
369	5794	2	9.00	6.00	0	.00
370	5794	2	10.00	6.00	2	-.26
371	5794	2	11.00	6.00	0	.00
372	5794	2	12.00	6.00	0	.00
373	5794	2	1.00	7.00	0	.00
374	5794	2	2.00	7.00	0	.00
375	5794	2	3.00	7.00	0	.00
376	5794	2	4.00	7.00	0	.00
377	5794	2	5.00	7.00	1	.00
378	5794	2	6.00	7.00	0	.00
379	5794	2	7.00	7.00	1	.01
380	5794	2	8.00	7.00	1	.07
381	?	?	?	?	?	?
382	?	?	?	?	?	?
383	?	?	?	?	?	?
384	?	?	?	?	?	?
385	?	?	?	?	?	?

	Duration_chw	P_or_V_value_chw	Base_value_chw	Amplitude	onset_percentage_chw	duration_percentage_chw
351	.00	.00	.00	.00	.00	.00
352	.00	.00	.00	.00	.00	.00
353	.80	18.63	-13.92	4.71	.13	100.00
354	.00	.00	.00	.00	.00	.00
355	.73	23.15	-18.33	4.82	-2.74	91.52
356	.64	22.32	-20.22	2.10	8.48	79.43
357	.00	.00	.00	.00	.00	.00
358	.78	-13.07	14.93	1.86	-27.93	97.13
359	.00	.00	.00	.00	.00	.00
360	.00	.00	.00	.00	.00	.00
361	.00	.00	.00	.00	.00	.00
362	.00	.00	.00	.00	.00	.00
363	.00	.00	.00	.00	.00	.00
364	.00	.00	.00	.00	.00	.00
365	.76	18.69	-14.42	4.27	.13	100.00
366	.00	.00	.00	.00	.00	.00
367	.67	23.49	-18.10	5.39	-.92	88.22
368	.60	22.20	-20.21	1.99	9.82	78.40
369	.00	.00	.00	.00	.00	.00
370	.79	-13.41	15.20	1.79	-33.38	103.93
371	.00	.00	.00	.00	.00	.00
372	.00	.00	.00	.00	.00	.00
373	.00	.00	.00	.00	.00	.00
374	.00	.00	.00	.00	.00	.00
375	.00	.00	.00	.00	.00	.00
376	.00	.00	.00	.00	.00	.00
377	.85	21.04	-14.41	6.63	.12	100.00
378	.00	.00	.00	.00	.00	.00
379	.83	25.75	-18.28	7.47	1.18	98.23
380	.73	23.17	-20.17	3.00	7.91	86.66
381	.00	.00	.00	.00	?	?
382	.70	-13.12	15.38	2.26	?	?
383	.00	.00	.00	.00	?	?
384	.00	.00	.00	.00	?	?
385	.00	.00	.00	.00	?	?

Sono Mastication_Raw data.sav

	amplitude_perc	chewing_side	Pig_code_sw	gender_sw	Pair_sw	Sw_episodes
351	.00	2.00	.	.	.	.
352	.00	1.00	.	.	.	.
353	25.28	5.00	.	.	.	.
354	.00	5.00	.	.	.	.
355	20.82	5.00	.	.	.	.
356	9.41	5.00	.	.	.	.
357	.00	2.00	.	.	.	.
358	-14.23	1.00	.	.	.	.
359	.00	2.00	.	.	.	.
360	.00	1.00	.	.	.	.
361	.00	2.00	.	.	.	.
362	.00	1.00	.	.	.	.
363	.00	2.00	.	.	.	.
364	.00	1.00	.	.	.	.
365	22.85	5.00	.	.	.	.
366	.00	5.00	.	.	.	.
367	22.95	5.00	.	.	.	.
368	8.96	5.00	.	.	.	.
369	.00	2.00	.	.	.	.
370	-13.35	1.00	.	.	.	.
371	.00	2.00	.	.	.	.
372	.00	1.00	.	.	.	.
373	.00	2.00	.	.	.	.
374	.00	1.00	.	.	.	.
375	.00	2.00	.	.	.	.
376	.00	1.00	.	.	.	.
377	31.51	5.00	.	.	.	.
378	.00	5.00	.	.	.	.
379	29.01	5.00	.	.	.	.
380	12.95	5.00	.	.	.	.
381	.00	2.00	.	.	.	.
382	-17.23	1.00	.	.	.	.
383	.00	2.00	.	.	.	.
384	.00	1.00	.	.	.	.
385	.00	2.00	.	.	.	.

Sono Mastication_Raw data.sav

	P_V_sw	Onset_sw	duration_sw	P_or_V_value_sw	Base_value_sw	Range
351	.	.	.	.	.	.
352	.	.	.	.	.	.
353	.	.	.	.	.	.
354	.	.	.	.	.	.
355	.	.	.	.	.	.
356	.	.	.	.	.	.
357	.	.	.	.	.	.
358	.	.	.	.	.	.
359	.	.	.	.	.	.
360	.	.	.	.	.	.
361	.	.	.	.	.	.
362	.	.	.	.	.	.
363	.	.	.	.	.	.
364	.	.	.	.	.	.
365	.	.	.	.	.	.
366	.	.	.	.	.	.
367	.	.	.	.	.	.
368	.	.	.	.	.	.
369	.	.	.	.	.	.
370	.	.	.	.	.	.
371	.	.	.	.	.	.
372	.	.	.	.	.	.
373	.	.	.	.	.	.
374	.	.	.	.	.	.
375	.	.	.	.	.	.
376	.	.	.	.	.	.
377	.	.	.	.	.	.
378	.	.	.	.	.	.
379	.	.	.	.	.	.
380	.	.	.	.	.	.
381	.	.	?	?	?	?
382	.	.	?	?	?	?
383	.	.	?	?	?	?
384	.	.	?	?	?	?
385	.	.	?	?	?	?

	onset_percent_sw	duration_percent_sw	Range_percent	Pause_before_sw	Pause_after_sw
351	.	.	.	.	.
352	.	.	.	.	.
353	.	.	.	.	.
354	.	.	.	.	.
355	.	.	.	.	.
356	.	.	.	.	.
357	.	.	.	.	.
358	.	.	.	.	.
359	.	.	.	.	.
360	.	.	.	.	.
361	.	.	.	.	.
362	.	.	.	.	.
363	.	.	.	.	.
364	.	.	.	.	.
365	.	.	.	.	.
366	.	.	.	.	.
367	.	.	.	.	.
368	.	.	.	.	.
369	.	.	.	.	.
370	.	.	.	.	.
371	.	.	.	.	.
372	.	.	.	.	.
373	.	.	.	.	.
374	.	.	.	.	.
375	.	.	.	.	.
376	.	.	.	.	.
377	.	.	.	.	.
378	.	.	.	.	.
379	.	.	.	.	.
380	.	.	.	.	.
381	.	.	.	.	.
382	.	.	.	.	.
383	.	.	.	.	.
384	.	.	.	.	.
385	.	.	.	.	.

	Pig_chw	Gender_chw	Pair_chw	Cycles	P_V_chw	Onset_chw
386	5794	2	2.00	8.00	0	.00
387	5794	2	3.00	8.00	0	.00
388	5794	2	4.00	8.00	0	.00
389	5794	2	5.00	8.00	1	.00
390	5794	2	6.00	8.00	0	.00
391	5794	2	7.00	8.00	1	.01
392	5794	2	8.00	8.00	1	.01
393	5794	2	9.00	8.00	0	.00
394	5794	2	10.00	8.00	2	-.82
395	5794	2	11.00	8.00	0	.00
396	5794	2	12.00	8.00	0	.00
397	5794	2	1.00	1.00	0	.00
398	5794	2	2.00	1.00	0	.00
399	5794	2	3.00	1.00	0	.00
400	5794	2	4.00	1.00	0	.00
401	5794	2	5.00	1.00	1	.00
402	5794	2	6.00	1.00	0	.00
403	5794	2	7.00	1.00	1	-.02
404	5794	2	8.00	1.00	1	.08
405	5794	2	9.00	1.00	0	.00
406	5794	2	10.00	1.00	2	-.16
407	5794	2	11.00	1.00	0	.00
408	5794	2	12.00	1.00	0	.00
409	5794	2	1.00	2.00	0	.00
410	5794	2	2.00	2.00	0	.00
411	5794	2	3.00	2.00	0	.00
412	5794	2	4.00	2.00	0	.00
413	5794	2	5.00	2.00	1	.00
414	5794	2	6.00	2.00	0	.00
415	5794	2	7.00	2.00	1	.01
416	5794	2	8.00	2.00	1	.17
417	5794	2	9.00	2.00	0	.00
418	5794	2	10.00	2.00	2	-.14
419	5794	2	11.00	2.00	0	.00
420	5794	2	12.00	2.00	0	.00

	Duration_chw	P_or_V_value_c hw	Base_value_c hw	Amplitude	onset_percen tage_chw	duration_perc entage_chw
386	.00	.00	.00	.00	.00	.00
387	.00	.00	.00	.00	.00	.00
388	.00	.00	.00	.00	.00	.00
389	.80	18.80	-15.36	3.44	.13	100.00
390	.00	.00	.00	.00	.00	.00
391	.80	22.45	-17.79	4.66	1.25	100.00
392	.74	21.82	-20.13	1.69	1.25	92.52
393	.00	.00	.00	.00	.00	.00
394	.75	-12.49	15.23	2.74	-32.74	93.39
395	.00	.00	.00	.00	.00	.00
396	.00	.00	.00	.00	.00	.00
397	.00	.00	.00	.00	.00	.00
398	.00	.00	.00	.00	.00	.00
399	.00	.00	.00	.00	.00	.00
400	.00	.00	.00	.00	.00	.00
401	.56	19.52	-15.36	4.16	.18	100.00
402	.00	.00	.00	.00	.00	.00
403	.58	26.97	-20.43	6.54	-4.09	103.92
404	.48	23.24	-21.27	1.97	13.35	85.41
405	.00	.00	.00	.00	.00	.00
406	.63	-13.17	15.84	2.67	-29.18	111.92
407	.00	.00	.00	.00	.00	.00
408	.00	.00	.00	.00	.00	.00
409	.00	.00	.00	.00	.00	.00
410	.00	.00	.00	.00	.00	.00
411	.00	.00	.00	.00	.00	.00
412	.00	.00	.00	.00	.00	.00
413	.64	19.19	-15.94	3.25	.16	100.00
414	.00	.00	.00	.00	.00	.00
415	.62	26.98	-21.63	5.35	1.57	97.65
416	.47	22.97	-21.43	1.54	27.16	73.00
417	.00	.00	.00	.00	.00	.00
418	.65	-13.14	15.67	2.53	-22.29	102.36
419	.00	.00	.00	.00	.00	.00
420	.00	.00	.00	.00	.00	.00

Sono Mastication_Raw data.sav

	amplitude_perc	chewing_side	Pig_code_sw	gender_sw	Pair_sw	Sw_episodes
386	.00	1.00	.	.	.	.
387	.00	2.00	.	.	.	.
388	.00	1.00	.	.	.	.
389	18.30	5.00	.	.	.	.
390	.00	5.00	.	.	.	.
391	20.76	5.00	.	.	.	.
392	7.75	5.00	.	.	.	.
393	.00	2.00	.	.	.	.
394	-21.94	1.00	.	.	.	.
395	.00	2.00	.	.	.	.
396	.00	1.00	.	.	.	.
397	.00	2.00	.	.	.	.
398	.00	1.00	.	.	.	.
399	.00	2.00	.	.	.	.
400	.00	1.00	.	.	.	.
401	21.31	5.00	.	.	.	.
402	.00	5.00	.	.	.	.
403	24.25	5.00	.	.	.	.
404	8.48	5.00	.	.	.	.
405	.00	2.00	.	.	.	.
406	-20.27	1.00	.	.	.	.
407	.00	2.00	.	.	.	.
408	.00	1.00	.	.	.	.
409	.00	2.00	.	.	.	.
410	.00	1.00	.	.	.	.
411	.00	2.00	.	.	.	.
412	.00	1.00	.	.	.	.
413	16.94	5.00	.	.	.	.
414	.00	5.00	.	.	.	.
415	19.83	5.00	.	.	.	.
416	6.70	5.00	.	.	.	.
417	.00	2.00	.	.	.	.
418	-19.25	1.00	.	.	.	.
419	.00	2.00	.	.	.	.
420	.00	1.00	.	.	.	.

Sono Mastication_Raw data.sav

	P_V_sw	Onset_sw	duration_sw	P_or_V_value_sw	Base_value_sw	Range
386	.	.	.	.	.	.
387	.	.	.	.	.	.
388	.	.	.	.	.	.
389	.	.	.	.	.	.
390	.	.	.	.	.	.
391	.	.	.	.	.	.
392	.	.	.	.	.	.
393	.	.	.	.	.	.
394	.	.	.	.	.	.
395	.	.	.	.	.	.
396	.	.	.	.	.	.
397	.	.	.	.	.	.
398	.	.	.	.	.	.
399	.	.	.	.	.	.
400	.	.	.	.	.	.
401	.	.	.	.	.	.
402	.	.	.	.	.	.
403	.	.	.	.	.	.
404	.	.	.	.	.	.
405	.	.	.	.	.	.
406	.	.	.	.	.	.
407	.	.	.	.	.	.
408	.	.	.	.	.	.
409	.	.	.	.	.	.
410	.	.	.	.	.	.
411	.	.	.	.	.	.
412	.	.	.	.	.	.
413	.	.	.	.	.	.
414	.	.	.	.	.	.
415	.	.	.	.	.	.
416	.	.	.	.	.	.
417	.	.	.	.	.	.
418	.	.	.	.	.	.
419	.	.	.	.	.	.
420	.	.	.	.	.	.

	onset_percent_sw	duration_percent_sw	Range_percent	Pause_before_sw	Pause_after_sw
386	.	.	.	.	.
387	.	.	.	.	.
388	.	.	.	.	.
389	.	.	.	.	.
390	.	.	.	.	.
391	.	.	.	.	.
392	.	.	.	.	.
393	.	.	.	.	.
394	.	.	.	.	.
395	.	.	.	.	.
396	.	.	.	.	.
397	.	.	.	.	.
398	.	.	.	.	.
399	.	.	.	.	.
400	.	.	.	.	.
401	.	.	.	.	.
402	.	.	.	.	.
403	.	.	.	.	.
404	.	.	.	.	.
405	.	.	.	.	.
406	.	.	.	.	.
407	.	.	.	.	.
408	.	.	.	.	.
409	.	.	.	.	.
410	.	.	.	.	.
411	.	.	.	.	.
412	.	.	.	.	.
413	.	.	.	.	.
414	.	.	.	.	.
415	.	.	.	.	.
416	.	.	.	.	.
417	.	.	.	.	.
418	.	.	.	.	.
419	.	.	.	.	.
420	.	.	.	.	.

	Pig_chw	Gender_chw	Pair_chw	Cycles	P_V_chw	Onset_chw
421	5794	2	1.00	3.00	0	.00
422	5794	2	2.00	3.00	0	.00
423	5794	2	3.00	3.00	0	.00
424	5794	2	4.00	3.00	0	.00
425	5794	2	5.00	3.00	1	.00
426	5794	2	6.00	3.00	0	.00
427	5794	2	7.00	3.00	1	.02
428	5794	2	8.00	3.00	1	.09
429	5794	2	9.00	3.00	0	.00
430	5794	2	10.00	3.00	2	-.20
431	5794	2	11.00	3.00	0	.00
432	5794	2	12.00	3.00	0	.00
433	5794	2	1.00	4.00	0	.00
434	5794	2	2.00	4.00	0	.00
435	5794	2	3.00	4.00	0	.00
436	5794	2	4.00	4.00	0	.00
437	5794	2	5.00	4.00	1	.00
438	5794	2	6.00	4.00	0	.00
439	5794	2	7.00	4.00	1	.00
440	5794	2	8.00	4.00	1	.05
441	5794	2	9.00	4.00	0	.00
442	5794	2	10.00	4.00	2	-.18
443	5794	2	11.00	4.00	0	.00
444	5794	2	12.00	4.00	0	.00
445	5794	2	1.00	5.00	0	.00
446	5794	2	2.00	5.00	0	.00
447	5794	2	3.00	5.00	0	.00
448	5794	2	4.00	5.00	0	.00
449	5794	2	5.00	5.00	1	.00
450	5794	2	6.00	5.00	0	.00
451	5794	2	7.00	5.00	1	-.03
452	5794	2	8.00	5.00	1	.05
453	5794	2	9.00	5.00	0	.00
454	5794	2	10.00	5.00	2	-.20
455	5794	2	11.00	5.00	0	.00

	Duration_chw	P_or_V_value_c hw	Base_value_c hw	Amplitude	onset_percen tage_chw	duration_perc entage_chw
421	.00	.00	.00	.00	.00	.00
422	.00	.00	.00	.00	.00	.00
423	.00	.00	.00	.00	.00	.00
424	.00	.00	.00	.00	.00	.00
425	.61	19.17	-15.16	4.01	.17	100.00
426	.00	.00	.00	.00	.00	.00
427	.58	26.35	-20.45	5.90	2.47	96.21
428	.51	22.92	-20.86	2.06	14.83	84.02
429	.00	.00	.00	.00	.00	.00
430	.68	-13.52	15.86	2.34	-32.13	112.36
431	.00	.00	.00	.00	.00	.00
432	.00	.00	.00	.00	.00	.00
433	.00	.00	.00	.00	.00	.00
434	.00	.00	.00	.00	.00	.00
435	.00	.00	.00	.00	.00	.00
436	.00	.00	.00	.00	.00	.00
437	.61	18.64	-15.08	3.56	.17	100.00
438	.00	.00	.00	.00	.00	.00
439	.56	26.12	-21.07	5.05	-.33	92.59
440	.50	23.22	-21.26	1.96	8.40	82.70
441	.00	.00	.00	.00	.00	.00
442	.68	-13.36	15.95	2.59	-29.98	112.36
443	.00	.00	.00	.00	.00	.00
444	.00	.00	.00	.00	.00	.00
445	.00	.00	.00	.00	.00	.00
446	.00	.00	.00	.00	.00	.00
447	.00	.00	.00	.00	.00	.00
448	.00	.00	.00	.00	.00	.00
449	.84	19.16	-14.43	4.73	.12	100.00
450	.00	.00	.00	.00	.00	.00
451	.84	26.67	-20.62	6.05	-2.98	100.00
452	.69	23.38	-20.51	2.87	6.32	82.12
453	.00	.00	.00	.00	.00	.00
454	.84	-12.61	15.67	3.06	-24.08	100.00
455	.00	.00	.00	.00	.00	.00

	amplitude_perc	chewing_side	Pig_code_sw	gender_sw	Pair_sw	Sw_episodes
421	.00	2.00	.	.	.	.
422	.00	1.00	.	.	.	.
423	.00	2.00	.	.	.	.
424	.00	1.00	.	.	.	.
425	20.92	5.00	.	.	.	.
426	.00	5.00	.	.	.	.
427	22.39	5.00	.	.	.	.
428	8.99	5.00	.	.	.	.
429	.00	2.00	.	.	.	.
430	-17.31	1.00	.	.	.	.
431	.00	2.00	.	.	.	.
432	.00	1.00	.	.	.	.
433	.00	2.00	.	.	.	.
434	.00	1.00	.	.	.	.
435	.00	2.00	.	.	.	.
436	.00	1.00	.	.	.	.
437	19.10	5.00	.	.	.	.
438	.00	5.00	.	.	.	.
439	19.33	5.00	.	.	.	.
440	8.44	5.00	.	.	.	.
441	.00	2.00	.	.	.	.
442	-19.39	1.00	.	.	.	.
443	.00	2.00	.	.	.	.
444	.00	1.00	.	.	.	.
445	.00	2.00	.	.	.	.
446	.00	1.00	.	.	.	.
447	.00	2.00	.	.	.	.
448	.00	1.00	.	.	.	.
449	24.69	5.00	.	.	.	.
450	.00	5.00	.	.	.	.
451	22.69	5.00	.	.	.	.
452	12.28	5.00	.	.	.	.
453	.00	2.00	.	.	.	.
454	-24.27	1.00	.	.	.	.
455	.00	2.00	.	.	.	.

	P_V_sw	Onset_sw	duration_sw	P_or_V_value_sw	Base_value_sw	Range
421	.	.	.	.	.	.
422	.	.	.	.	.	.
423	.	.	.	.	.	.
424	.	.	.	.	.	.
425	.	.	.	.	.	.
426	.	.	.	.	.	.
427	.	.	.	.	.	.
428	.	.	.	.	.	.
429	.	.	.	.	.	.
430	.	.	.	.	.	.
431	.	.	.	.	.	.
432	.	.	.	.	.	.
433	.	.	.	.	.	.
434	.	.	.	.	.	.
435	.	.	.	.	.	.
436	.	.	.	.	.	.
437	.	.	.	.	.	.
438	.	.	.	.	.	.
439	.	.	.	.	.	.
440	.	.	.	.	.	.
441	.	.	?	?	?	?
442	.	.	?	?	?	?
443	.	.	?	?	?	?
444	.	.	?	?	?	?
445	.	.	?	?	?	?
446	.	.	?	?	?	?
447	.	.	.	.	.	.
448	.	.	.	.	.	.
449	.	.	.	.	.	.
450	.	.	.	.	.	.
451	.	.	.	.	.	.
452	.	.	.	.	.	.
453	.	.	.	.	.	.
454	.	.	.	.	.	.
455	.	.	.	.	.	.

	onset_percent_sw	duration_percent_sw	Range_percent	Pause_before_sw	Pause_after_sw
421	.	.	.	.	.
422	.	.	.	.	.
423	.	.	.	.	.
424	.	.	.	.	.
425	.	.	.	.	.
426	.	.	.	.	.
427	.	.	.	.	.
428	.	.	.	.	.
429	.	.	.	.	.
430	.	.	.	.	.
431	.	.	.	.	.
432	.	.	.	.	.
433	.	.	.	.	.
434	.	.	.	.	.
435	.	.	.	.	.
436	.	.	.	.	.
437	.	.	.	.	.
438	.	.	.	.	.
439	.	.	.	.	.
440	.	.	.	.	.
441	.	.	.	.	.
442	.	.	.	.	.
443	.	.	.	.	.
444	.	.	.	.	.
445	.	.	.	.	.
446	.	.	.	.	.
447	.	.	.	.	.
448	.	.	.	.	.
449	.	.	.	.	.
450	.	.	.	.	.
451	.	.	.	.	.
452	.	.	.	.	.
453	.	.	.	.	.
454	.	.	.	.	.
455	.	.	.	.	.

Sono Mastication_Raw data.sav

	Pig_chw	Gender_chw	Pair_chw	Cycles	P_V_chw	Onset_chw
456	5794	2	12.00	5.00	0	.00
457	5794	2	1.00	6.00	0	.00
458	5794	2	2.00	6.00	0	.00
459	5794	2	3.00	6.00	0	.00
460	5794	2	4.00	6.00	0	.00
461	5794	2	5.00	6.00	1	.00
462	5794	2	6.00	6.00	0	.00
463	5794	2	7.00	6.00	1	-.07
464	5794	2	8.00	6.00	1	-.01
465	5794	2	9.00	6.00	0	.00
466	5794	2	10.00	6.00	2	-.26
467	5794	2	11.00	6.00	0	.00
468	5794	2	12.00	6.00	0	.00
469	5794	2	1.00	7.00	0	.00
470	5794	2	2.00	7.00	0	.00
471	5794	2	3.00	7.00	0	.00
472	5794	2	4.00	7.00	0	.00
473	5794	2	5.00	7.00	1	.00
474	5794	2	6.00	7.00	0	.00
475	5794	2	7.00	7.00	1	.02
476	5794	2	8.00	7.00	1	.10
477	5794	2	9.00	7.00	0	.00
478	5794	2	10.00	7.00	2	-.18
479	5794	2	11.00	7.00	0	.00
480	5794	2	12.00	7.00	0	.00
481	?	?	?	?	?	?
482	?	?	?	?	?	?
483	?	?	?	?	?	?
484	?	?	?	?	?	?
485	?	?	?	?	?	?
486	?	?	?	?	?	?
487	?	?	?	?	?	?
488	?	?	?	?	?	?
489	?	?	?	?	?	?
490	?	?	?	?	?	?

	Duration_chw	P_or_V_value_chw	Base_value_chw	Amplitude	onset_percentage_chw	duration_percentage_chw
456	.00	.00	.00	.00	.00	.00
457	.00	.00	.00	.00	.00	.00
458	.00	.00	.00	.00	.00	.00
459	.00	.00	.00	.00	.00	.00
460	.00	.00	.00	.00	.00	.00
461	.50	17.79	-14.36	3.43	.20	100.00
462	.00	.00	.00	.00	.00	.00
463	.53	24.80	-20.24	4.56	-14.54	104.58
464	.41	22.96	-21.08	1.88	-1.20	82.07
465	.00	.00	.00	.00	.00	.00
466	.61	-13.27	15.13	1.86	-31.79	122.31
467	.00	.00	.00	.00	.00	.00
468	.00	.00	.00	.00	.00	.00
469	.00	.00	.00	.00	.00	.00
470	.00	.00	.00	.00	.00	.00
471	.00	.00	.00	.00	.00	.00
472	.00	.00	.00	.00	.00	.00
473	.82	20.80	-14.87	5.93	.12	100.00
474	.00	.00	.00	.00	.00	.00
475	.82	26.34	-20.13	6.21	2.69	100.00
476	.69	24.05	-21.06	2.99	11.87	84.33
477	.00	.00	.00	.00	.00	.00
478	.59	-13.22	15.55	2.33	-22.03	72.46
479	.00	.00	.00	.00	.00	.00
480	.00	.00	.00	.00	.00	.00
481	.00	.00	.00	.00	?	?
482	.00	.00	.00	.00	?	?
483	.00	.00	.00	.00	?	?
484	.00	.00	.00	.00	?	?
485	.38	17.64	-15.70	1.94	?	?
486	.00	.00	.00	.00	?	?
487	.33	21.35	-19.13	2.22	?	?
488	.00	.00	.00	.00	?	?
489	.00	.00	.00	.00	?	?
490	.47	-13.12	14.77	1.65	?	?

	amplitude_perc	chewing_side	Pig_code_sw	gender_sw	Pair_sw	Sw_episodes
456	.00	1.00	.	.	.	.
457	.00	2.00	.	.	.	.
458	.00	1.00	.	.	.	.
459	.00	2.00	.	.	.	.
460	.00	1.00	.	.	.	.
461	19.28	5.00	.	.	.	.
462	.00	5.00	.	.	.	.
463	18.39	5.00	.	.	.	.
464	8.19	5.00	.	.	.	.
465	.00	2.00	.	.	.	.
466	-14.02	1.00	.	.	.	.
467	.00	2.00	.	.	.	.
468	.00	1.00	.	.	.	.
469	.00	2.00	.	.	.	.
470	.00	1.00	.	.	.	.
471	.00	2.00	.	.	.	.
472	.00	1.00	.	.	.	.
473	28.51	5.00	.	.	.	.
474	.00	5.00	.	.	.	.
475	23.58	5.00	.	.	.	.
476	12.43	5.00	.	.	.	.
477	.00	2.00	.	.	.	.
478	-17.63	1.00	.	.	.	.
479	.00	2.00	.	.	.	.
480	.00	1.00	.	.	.	.
481	.00	2.00	.	.	.	.
482	.00	1.00	.	.	.	.
483	.00	2.00	.	.	.	.
484	.00	1.00	.	.	.	.
485	11.00	5.00	.	.	.	.
486	.00	5.00	.	.	.	.
487	10.40	5.00	.	.	.	.
488	.00	5.00	.	.	.	.
489	.00	2.00	.	.	.	.
490	-12.58	1.00	.	.	.	.

	P_V_sw	Onset_sw	duration_sw	P_or_V_value_sw	Base_value_sw	Range
456	.	.	.	.	.	.
457	.	.	.	.	.	.
458	.	.	.	.	.	.
459	.	.	.	.	.	.
460	.	.	.	.	.	.
461	.	.	.	.	.	.
462	.	.	.	.	.	.
463	.	.	.	.	.	.
464	.	.	.	.	.	.
465	.	.	.	.	.	.
466	.	.	.	.	.	.
467	.	.	.	.	.	.
468	.	.	.	.	.	.
469	.	.	.	.	.	.
470	.	.	.	.	.	.
471	.	.	.	.	.	.
472	.	.	.	.	.	.
473	.	.	.	.	.	.
474	.	.	.	.	.	.
475	.	.	.	.	.	.
476	.	.	.	.	.	.
477	.	.	.	.	.	.
478	.	.	.	.	.	.
479	.	.	.	.	.	.
480	.	.	.	.	.	.
481	.	.	?	?	?	?
482	.	.	?	?	?	?
483	.	.	?	?	?	?
484	.	.	?	?	?	?
485	.	.	?	?	?	?
486	.	.	?	?	?	?
487	.	.	?	?	?	?
488	.	.	?	?	?	?
489	.	.	?	?	?	?
490	.	.	?	?	?	?

	onset_percent_sw	duration_percent_sw	Range_percent	Pause_before_sw	Pause_after_sw
456	.	.	.	.	.
457	.	.	.	.	.
458	.	.	.	.	.
459	.	.	.	.	.
460	.	.	.	.	.
461	.	.	.	.	.
462	.	.	.	.	.
463	.	.	.	.	.
464	.	.	.	.	.
465	.	.	.	.	.
466	.	.	.	.	.
467	.	.	.	.	.
468	.	.	.	.	.
469	.	.	.	.	.
470	.	.	.	.	.
471	.	.	.	.	.
472	.	.	.	.	.
473	.	.	.	.	.
474	.	.	.	.	.
475	.	.	.	.	.
476	.	.	.	.	.
477	.	.	.	.	.
478	.	.	.	.	.
479	.	.	.	.	.
480	.	.	.	.	.
481	.	.	.	.	.
482	.	.	.	.	.
483	.	.	.	.	.
484	.	.	.	.	.
485	.	.	.	.	.
486	.	.	.	.	.
487	.	.	.	.	.
488	.	.	.	.	.
489	.	.	.	.	.
490	.	.	.	.	.

Sono Mastication_Raw data.sav

	Pig_chw	Gender_chw	Pair_chw	Cycles	P_V_chw	Onset_chw
491	5794	2	11.00	8.00	0	.00
492	5794	2	12.00	8.00	0	.00
493	5794	2	1.00	9.00	0	.00
494	5794	2	2.00	9.00	0	.00
495	5794	2	3.00	9.00	0	.00
496	5794	2	4.00	9.00	0	.00
497	5794	2	5.00	9.00	1	.00
498	5794	2	6.00	9.00	0	.00
499	5794	2	7.00	9.00	1	.02
500	5794	2	8.00	9.00	0	.00
501	5794	2	9.00	9.00	0	.00
502	5794	2	10.00	9.00	2	-.13
503	5794	2	11.00	9.00	0	.00
504	5794	2	12.00	9.00	0	.00
505	5794	2	1.00	10.00	0	.00
506	5794	2	2.00	10.00	0	.00
507	5794	2	3.00	10.00	0	.00
508	5794	2	4.00	10.00	0	.00
509	5794	2	5.00	10.00	1	.00
510	5794	2	6.00	10.00	0	.00
511	5794	2	7.00	10.00	0	.00
512	5794	2	8.00	10.00	1	.07
513	5794	2	9.00	10.00	0	.00
514	5794	2	10.00	10.00	2	-.14
515	5794	2	11.00	10.00	0	.00
516	5794	2	12.00	10.00	0	.00
517	5794	2	1.00	11.00	0	.00
518	5794	2	2.00	11.00	0	.00
519	5794	2	3.00	11.00	0	.00
520	5794	2	4.00	11.00	0	.00
521	?	?	?	?	?	?
522	?	?	?	?	?	?
523	?	?	?	?	?	?
524	?	?	?	?	?	?
525	?	?	?	?	?	?

Sono Mastication_Raw data.sav

	Duration_chw	P_or_V_value_chw	Base_value_chw	Amplitude	onset_percentage_chw	duration_percentage_chw
491	.00	.00	.00	.00	.00	.00
492	.00	.00	.00	.00	.00	.00
493	.00	.00	.00	.00	.00	.00
494	.00	.00	.00	.00	.00	.00
495	.00	.00	.00	.00	.00	.00
496	.00	.00	.00	.00	.00	.00
497	.47	18.17	-16.08	2.09	.21	100.00
498	.00	.00	.00	.00	.00	.00
499	.39	22.45	-19.22	3.23	4.66	83.05
500	.00	.00	.00	.00	.00	.00
501	.00	.00	.00	.00	.00	.00
502	.50	-13.21	15.02	1.81	-26.91	106.36
503	.00	.00	.00	.00	.00	.00
504	.00	.00	.00	.00	.00	.00
505	.00	.00	.00	.00	.00	.00
506	.00	.00	.00	.00	.00	.00
507	.00	.00	.00	.00	.00	.00
508	.00	.00	.00	.00	.00	.00
509	.69	19.43	-14.60	4.83	.15	100.00
510	.00	.00	.00	.00	.00	.00
511	.00	.00	.00	.00	.00	.00
512	.56	22.63	-20.69	1.94	9.87	80.55
513	.00	.00	.00	.00	.00	.00
514	.66	-12.37	15.47	3.10	-20.61	95.65
515	.00	.00	.00	.00	.00	.00
516	.00	.00	.00	.00	.00	.00
517	.00	.00	.00	.00	.00	.00
518	.00	.00	.00	.00	.00	.00
519	.00	.00	.00	.00	.00	.00
520	.00	.00	.00	.00	.00	.00
521	.67	19.70	-15.04	4.66	?	?
522	.00	.00	.00	.00	?	?
523	.60	24.50	-20.16	4.34	?	?
524	.52	23.10	-20.89	2.21	?	?
525	.00	.00	.00	.00	?	?

Sono Mastication_Raw data.sav

	amplitude_perc	chewing_side	Pig_code_sw	gender_sw	Pair_sw	Sw_episodes
491	.00	2.00	.	.	.	.
492	.00	1.00	.	.	.	.
493	.00	4.00	.	.	.	.
494	.00	3.00	.	.	.	.
495	.00	4.00	.	.	.	.
496	.00	3.00	.	.	.	.
497	11.50	5.00	.	.	.	.
498	.00	5.00	.	.	.	.
499	14.39	5.00	.	.	.	.
500	.00	5.00	.	.	.	.
501	.00	4.00	.	.	.	.
502	-13.70	3.00	.	.	.	.
503	.00	4.00	.	.	.	.
504	.00	3.00	.	.	.	.
505	.00	4.00	.	.	.	.
506	.00	3.00	.	.	.	.
507	.00	4.00	.	.	.	.
508	.00	3.00	.	.	.	.
509	24.86	5.00	.	.	.	.
510	.00	5.00	.	.	.	.
511	.00	5.00	.	.	.	.
512	8.57	5.00	.	.	.	.
513	.00	4.00	.	.	.	.
514	-25.06	3.00	.	.	.	.
515	.00	4.00	.	.	.	.
516	.00	3.00	.	.	.	.
517	.00	2.00	.	.	.	.
518	.00	1.00	.	.	.	.
519	.00	2.00	.	.	.	.
520	.00	1.00	.	.	.	.
521	23.66	5.00	.	.	.	.
522	.00	5.00	.	.	.	.
523	17.71	5.00	.	.	.	.
524	9.57	5.00	.	.	.	.
525	.00	2.00	.	.	.	.

	P_V_sw	Onset_sw	duration_sw	P_or_V_value_sw	Base_value_sw	Range
491	.	.	.	.	.	.
492	.	.	.	.	.	.
493	.	.	.	.	.	.
494	.	.	.	.	.	.
495	.	.	.	.	.	.
496	.	.	.	.	.	.
497	.	.	.	.	.	.
498	.	.	.	.	.	.
499	.	.	.	.	.	.
500	.	.	.	.	.	.
501	.	.	.	.	.	.
502	.	.	.	.	.	.
503	.	.	.	.	.	.
504	.	.	.	.	.	.
505	.	.	.	.	.	.
506	.	.	.	.	.	.
507	.	.	.	.	.	.
508	.	.	.	.	.	.
509	.	.	.	.	.	.
510	.	.	.	.	.	.
511	.	.	.	.	.	.
512	.	.	.	.	.	.
513	.	.	.	.	.	.
514	.	.	.	.	.	.
515	.	.	.	.	.	.
516	.	.	.	.	.	.
517	.	.	.	.	.	.
518	.	.	.	.	.	.
519	.	.	.	.	.	.
520	.	.	.	.	.	.
521	.	.	?	?	?	?
522	.	.	?	?	?	?
523	.	.	?	?	?	?
524	.	.	?	?	?	?
525	.	.	?	?	?	?

Sono Mastication_Raw data.sav

	onset_percent_sw	duration_percent_sw	Range_percent	Pause_before_sw	Pause_after_sw
491	.	.	.	.	.
492	.	.	.	.	.
493	.	.	.	.	.
494	.	.	.	.	.
495	.	.	.	.	.
496	.	.	.	.	.
497	.	.	.	.	.
498	.	.	.	.	.
499	.	.	.	.	.
500	.	.	.	.	.
501	.	.	.	.	.
502	.	.	.	.	.
503	.	.	.	.	.
504	.	.	.	.	.
505	.	.	.	.	.
506	.	.	.	.	.
507	.	.	.	.	.
508	.	.	.	.	.
509	.	.	.	.	.
510	.	.	.	.	.
511	.	.	.	.	.
512	.	.	.	.	.
513	.	.	.	.	.
514	.	.	.	.	.
515	.	.	.	.	.
516	.	.	.	.	.
517	.	.	.	.	.
518	.	.	.	.	.
519	.	.	.	.	.
520	.	.	.	.	.
521	.	.	.	.	.
522	.	.	.	.	.
523	.	.	.	.	.
524	.	.	.	.	.
525	.	.	.	.	.

	Pig_chw	Gender_chw	Pair_chw	Cycles	P_V_chw	Onset_chw
526	5794	2	10.00	11.00	2	.17
527	5794	2	11.00	11.00	0	.00
528	5794	2	12.00	11.00	0	.00
529	5794	2	1.00	12.00	0	.00
530	5794	2	2.00	12.00	0	.00
531	5794	2	3.00	12.00	0	.00
532	5794	2	4.00	12.00	0	.00
533	5794	2	5.00	12.00	1	.00
534	5794	2	6.00	12.00	0	.00
535	5794	2	7.00	12.00	1	-.01
536	5794	2	8.00	12.00	1	.06
537	5794	2	9.00	12.00	0	.00
538	5794	2	10.00	12.00	2	-.08
539	5794	2	11.00	12.00	0	.00
540	5794	2	12.00	12.00	0	.00
541	5794	2	1.00	13.00	0	.00
542	5794	2	2.00	13.00	0	.00
543	5794	2	3.00	13.00	0	.00
544	5794	2	4.00	13.00	0	.00
545	5794	2	5.00	13.00	1	.00
546	5794	2	6.00	13.00	0	.00
547	5794	2	7.00	13.00	1	.05
548	5794	2	8.00	13.00	0	.00
549	5794	2	9.00	13.00	0	.00
550	5794	2	10.00	13.00	2	.05
551	5794	2	11.00	13.00	0	.00
552	5794	2	12.00	13.00	0	.00
553	6487	2	1.00	1.00	0	.00
554	6487	2	2.00	1.00	2	-.01
555	6487	2	3.00	1.00	0	.00
556	6487	2	4.00	1.00	0	.00
557	6487	2	5.00	1.00	1	.00
558	6487	2	6.00	1.00	0	.00
559	6487	2	7.00	1.00	0	.00
560	6487	2	8.00	1.00	0	.00

	Duration_chw	P_or_V_value_c hw	Base_value_c hw	Amplitude	onset_percen tage_chw	duration_perc entage_chw
526	.64	-12.54	15.34	2.80	-25.52	94.51
527	.00	.00	.00	.00	.00	.00
528	.00	.00	.00	.00	.00	.00
529	.00	.00	.00	.00	.00	.00
530	.00	.00	.00	.00	.00	.00
531	.00	.00	.00	.00	.00	.00
532	.00	.00	.00	.00	.00	.00
533	.46	19.15	-14.39	4.76	.22	100.00
534	.00	.00	.00	.00	.00	.00
535	.44	23.75	-19.18	4.57	-1.53	95.62
536	.38	23.12	-21.09	2.03	13.13	83.59
537	.00	.00	.00	.00	.00	.00
538	.44	-13.39	14.44	1.05	-16.41	96.72
539	.00	.00	.00	.00	.00	.00
540	.00	.00	.00	.00	.00	.00
541	.00	.00	.00	.00	.00	.00
542	.00	.00	.00	.00	.00	.00
543	.00	.00	.00	.00	.00	.00
544	.00	.00	.00	.00	.00	.00
545	.46	18.22	-16.19	2.03	.22	100.00
546	.00	.00	.00	.00	.00	.00
547	.35	21.58	-20.32	1.26	9.85	77.02
548	.00	.00	.00	.00	.00	.00
549	.00	.00	.00	.00	.00	.00
550	.29	-14.11	15.63	1.52	11.38	63.90
551	.00	.00	.00	.00	.00	.00
552	.00	.00	.00	.00	.00	.00
553	.00	.00	.00	.00	.00	.00
554	.56	38.81	-29.59	-9.22	-1.26	101.44
555	.00	.00	.00	.00	.00	.00
556	.00	.00	.00	.00	.00	.00
557	.55	30.49	-26.79	3.70	.18	100.00
558	.00	.00	.00	.00	.00	.00
559	.00	.00	.00	.00	.00	.00
560	.00	.00	.00	.00	.00	.00

Sono Mastication_Raw data.sav

	amplitude_perc	chewing_side	Pig_code_sw	gender_sw	Pair_sw	Sw_episodes
526	-22.33	1.00	.	.	.	.
527	.00	2.00	.	.	.	.
528	.00	1.00	.	.	.	.
529	.00	2.00	.	.	.	.
530	.00	1.00	.	.	.	.
531	.00	2.00	.	.	.	.
532	.00	1.00	.	.	.	.
533	24.86	5.00	.	.	.	.
534	.00	5.00	.	.	.	.
535	19.24	5.00	.	.	.	.
536	8.78	5.00	.	.	.	.
537	.00	2.00	.	.	.	.
538	-7.84	1.00	.	.	.	.
539	.00	2.00	.	.	.	.
540	.00	1.00	.	.	.	.
541	.00	2.00	.	.	.	.
542	.00	1.00	.	.	.	.
543	.00	2.00	.	.	.	.
544	.00	1.00	.	.	.	.
545	11.14	5.00	.	.	.	.
546	.00	5.00	.	.	.	.
547	5.84	5.00	.	.	.	.
548	.00	5.00	.	.	.	.
549	.00	2.00	.	.	.	.
550	-10.77	1.00	.	.	.	.
551	.00	2.00	.	.	.	.
552	.00	1.00	.	.	.	.
553	.00	4.00	.	.	.	.
554	-23.76	3.00	.	.	.	.
555	.00	4.00	.	.	.	.
556	.00	3.00	.	.	.	.
557	12.14	5.00	.	.	.	.
558	.00	5.00	.	.	.	.
559	.00	5.00	.	.	.	.
560	.00	5.00	.	.	.	.

	P_V_sw	Onset_sw	duration_sw	P_or_V_value_sw	Base_value_sw	Range
526	.	.	.	.	.	.
527	.	.	.	.	.	.
528	.	.	.	.	.	.
529	.	.	.	.	.	.
530	.	.	.	.	.	.
531	.	.	.	.	.	.
532	.	.	.	.	.	.
533	.	.	.	.	.	.
534	.	.	.	.	.	.
535	.	.	.	.	.	.
536	.	.	.	.	.	.
537	.	.	.	.	.	.
538	.	.	.	.	.	.
539	.	.	.	.	.	.
540	.	.	.	.	.	.
541	.	.	.	.	.	.
542	.	.	.	.	.	.
543	.	.	.	.	.	.
544	.	.	.	.	.	.
545	.	.	.	.	.	.
546	.	.	.	.	.	.
547	.	.	.	.	.	.
548	.	.	.	.	.	.
549	.	.	.	.	.	.
550	.	.	.	.	.	.
551	.	.	.	.	.	.
552	.	.	.	.	.	.
553	.	.	.	.	.	.
554	.	.	.	.	.	.
555	.	.	.	.	.	.
556	.	.	.	.	.	.
557	.	.	.	.	.	.
558	.	.	.	.	.	.
559	.	.	.	.	.	.
560	.	.	.	.	.	.

	onset_percent_sw	duration_percent_sw	Range_percent	Pause_before_sw	Pause_after_sw
526	.	.	.	.	.
527	.	.	.	.	.
528	.	.	.	.	.
529	.	.	.	.	.
530	.	.	.	.	.
531	.	.	.	.	.
532	.	.	.	.	.
533	.	.	.	.	.
534	.	.	.	.	.
535	.	.	.	.	.
536	.	.	.	.	.
537	.	.	.	.	.
538	.	.	.	.	.
539	.	.	.	.	.
540	.	.	.	.	.
541	.	.	.	.	.
542	.	.	.	.	.
543	.	.	.	.	.
544	.	.	.	.	.
545	.	.	.	.	.
546	.	.	.	.	.
547	.	.	.	.	.
548	.	.	.	.	.
549	.	.	.	.	.
550	.	.	.	.	.
551	.	.	.	.	.
552	.	.	.	.	.
553	.	.	.	.	.
554	.	.	.	.	.
555	.	.	.	.	.
556	.	.	.	.	.
557	.	.	.	.	.
558	.	.	.	.	.
559	.	.	.	.	.
560	.	.	.	.	.

Sono Mastication_Raw data.sav

	Pig_chw	Gender_chw	Pair_chw	Cycles	P_V_chw	Onset_chw
561	6487	2	9.00	1.00	2	-.03
562	6487	2	10.00	1.00	2	-.02
563	6487	2	11.00	1.00	0	.00
564	6487	2	12.00	1.00	0	.00
565	6487	2	1.00	2.00	0	.00
566	6487	2	2.00	2.00	1	.00
567	6487	2	3.00	2.00	0	.00
568	6487	2	4.00	2.00	0	.00
569	6487	2	5.00	2.00	1	.00
570	6487	2	6.00	2.00	0	.00
571	6487	2	7.00	2.00	0	.00
572	6487	2	8.00	2.00	0	.00
573	6487	2	9.00	2.00	2	-.02
574	6487	2	10.00	2.00	2	.01
575	6487	2	11.00	2.00	0	.00
576	6487	2	12.00	2.00	0	.00
577	6487	2	1.00	3.00	0	.00
578	6487	2	2.00	3.00	2	.01
579	6487	2	3.00	3.00	0	.00
580	6487	2	4.00	3.00	0	.00
581	6487	2	5.00	3.00	1	.00
582	6487	2	6.00	3.00	0	.00
583	6487	2	7.00	3.00	0	.00
584	6487	2	8.00	3.00	0	.00
585	6487	2	9.00	3.00	2	-.01
586	6487	2	10.00	3.00	2	.01
587	6487	2	11.00	3.00	0	.00
588	6487	2	12.00	3.00	0	.00
589	6487	2	1.00	4.00	0	.00
590	6487	2	2.00	4.00	2	.01
591	6487	2	3.00	4.00	0	.00
592	6487	2	4.00	4.00	0	.00
593	6487	2	5.00	4.00	1	.00
594	6487	2	6.00	4.00	0	.00
595	6487	2	7.00	4.00	0	.00

	Duration_chw	P_or_V_value_chw	Base_value_chw	Amplitude	onset_percentage_chw	duration_percentage_chw
561	.56	-13.80	17.58	3.78	-5.42	101.44
562	.57	35.44	-30.04	5.40	-2.71	102.71
563	.00	.00	.00	.00	.00	.00
564	.00	.00	.00	.00	.00	.00
565	.00	.00	.00	.00	.00	.00
566	.58	37.70	-30.65	-7.05	.00	98.80
567	.00	.00	.00	.00	.00	.00
568	.00	.00	.00	.00	.00	.00
569	.58	30.64	-27.48	3.16	.17	100.00
570	.00	.00	.00	.00	.00	.00
571	.00	.00	.00	.00	.00	.00
572	.00	.00	.00	.00	.00	.00
573	.56	-13.02	17.39	4.37	-2.57	96.23
574	.58	38.08	-31.15	6.93	1.71	100.00
575	.00	.00	.00	.00	.00	.00
576	.00	.00	.00	.00	.00	.00
577	.00	.00	.00	.00	.00	.00
578	.61	37.49	-30.54	-6.95	1.17	101.34
579	.00	.00	.00	.00	.00	.00
580	.00	.00	.00	.00	.00	.00
581	.60	30.84	-27.07	3.77	.17	100.00
582	.00	.00	.00	.00	.00	.00
583	.00	.00	.00	.00	.00	.00
584	.00	.00	.00	.00	.00	.00
585	.60	-13.14	17.27	4.13	-1.34	100.00
586	.59	37.75	-31.12	6.63	1.17	98.83
587	.00	.00	.00	.00	.00	.00
588	.00	.00	.00	.00	.00	.00
589	.00	.00	.00	.00	.00	.00
590	.58	38.87	-30.28	-8.59	1.18	98.65
591	.00	.00	.00	.00	.00	.00
592	.00	.00	.00	.00	.00	.00
593	.59	30.74	-26.79	3.95	.17	100.00
594	.00	.00	.00	.00	.00	.00
595	.00	.00	.00	.00	.00	.00

Sono Mastication_Raw data.sav

	amplitude_perc	chewing_side	Pig_code_sw	gender_sw	Pair_sw	Sw_episodes
561	-27.39	4.00	.	.	.	.
562	15.24	3.00	.	.	.	.
563	.00	4.00	.	.	.	.
564	.00	3.00	.	.	.	.
565	.00	4.00	.	.	.	.
566	-18.70	3.00	.	.	.	.
567	.00	4.00	.	.	.	.
568	.00	3.00	.	.	.	.
569	10.31	5.00	.	.	.	.
570	.00	5.00	.	.	.	.
571	.00	5.00	.	.	.	.
572	.00	5.00	.	.	.	.
573	-33.56	4.00	.	.	.	.
574	-18.20	3.00	.	.	.	.
575	.00	4.00	.	.	.	.
576	.00	3.00	.	.	.	.
577	.00	2.00	.	.	.	.
578	-18.54	1.00	.	.	.	.
579	.00	2.00	.	.	.	.
580	.00	1.00	.	.	.	.
581	12.22	5.00	.	.	.	.
582	.00	5.00	.	.	.	.
583	.00	5.00	.	.	.	.
584	.00	5.00	.	.	.	.
585	-31.43	2.00	.	.	.	.
586	-17.56	1.00	.	.	.	.
587	.00	2.00	.	.	.	.
588	.00	1.00	.	.	.	.
589	.00	2.00	.	.	.	.
590	-22.10	1.00	.	.	.	.
591	.00	2.00	.	.	.	.
592	.00	1.00	.	.	.	.
593	12.85	5.00	.	.	.	.
594	.00	5.00	.	.	.	.
595	.00	5.00	.	.	.	.

Sono Mastication_Raw data.sav

	P_V_sw	Onset_sw	duration_sw	P_or_V_value_sw	Base_value_sw	Range
561	.	.	.	.	.	.
562	.	.	.	.	.	.
563	.	.	.	.	.	.
564	.	.	.	.	.	.
565	.	.	.	.	.	.
566	.	.	.	.	.	.
567	.	.	.	.	.	.
568	.	.	.	.	.	.
569	.	.	.	.	.	.
570	.	.	.	.	.	.
571	.	.	.	.	.	.
572	.	.	.	.	.	.
573	.	.	.	.	.	.
574	.	.	.	.	.	.
575	.	.	.	.	.	.
576	.	.	.	.	.	.
577	.	.	.	.	.	.
578	.	.	.	.	.	.
579	.	.	.	.	.	.
580	.	.	.	.	.	.
581	.	.	?	?	?	?
582	.	.	?	?	.	.
583	.	.	.	.	.	.
584	.	.	.	.	.	.
585	.	.	.	.	.	.
586	.	.	.	.	.	.
587	.	.	.	.	.	.
588	.	.	.	.	.	.
589	.	.	.	.	.	.
590	.	.	.	.	.	.
591	.	.	.	.	.	.
592	.	.	.	.	.	.
593	.	.	.	.	.	.
594	.	.	.	.	.	.
595	.	.	.	.	.	.

Sono Mastication_Raw data.sav

	onset_percent_sw	duration_percent_sw	Range_percent	Pause_before_sw	Pause_after_sw
561	.	.	.	.	.
562	.	.	.	.	.
563	.	.	.	.	.
564	.	.	.	.	.
565	.	.	.	.	.
566	.	.	.	.	.
567	.	.	.	.	.
568	.	.	.	.	.
569	.	.	.	.	.
570	.	.	.	.	.
571	.	.	.	.	.
572	.	.	.	.	.
573	.	.	.	.	.
574	.	.	.	.	.
575	.	.	.	.	.
576	.	.	.	.	.
577	.	.	.	.	.
578	.	.	.	.	.
579	.	.	.	.	.
580	.	.	.	.	.
581	.	.	.	.	.
582	.	.	.	.	.
583	.	.	.	.	.
584	.	.	.	.	.
585	.	.	.	.	.
586	.	.	.	.	.
587	.	.	.	.	.
588	.	.	.	.	.
589	.	.	.	.	.
590	.	.	.	.	.
591	.	.	.	.	.
592	.	.	.	.	.
593	.	.	.	.	.
594	.	.	.	.	.
595	.	.	.	.	.

Sono Mastication_Raw data.sav

	Pig_chw	Gender_chw	Pair_chw	Cycles	P_V_chw	Onset_chw
596	6487	2	8.00	4.00	0	.00
597	6487	2	9.00	4.00	2	-.02
598	6487	2	10.00	4.00	2	.01
599	6487	2	11.00	4.00	0	.00
600	6487	2	12.00	4.00	0	.00
601	6487	2	1.00	5.00	0	.00
602	6487	2	2.00	5.00	2	.01
603	6487	2	3.00	5.00	0	.00
604	6487	2	4.00	5.00	0	.00
605	6487	2	5.00	5.00	1	.00
606	6487	2	6.00	5.00	0	.00
607	6487	2	7.00	5.00	0	.00
608	6487	2	8.00	5.00	0	.00
609	6487	2	9.00	5.00	2	-.02
610	6487	2	10.00	5.00	2	.01
611	6487	2	11.00	5.00	0	.00
612	6487	2	12.00	5.00	0	.00
613	6487	2	1.00	6.00	0	.00
614	6487	2	2.00	6.00	2	.01
615	6487	2	3.00	6.00	0	.00
616	6487	2	4.00	6.00	0	.00
617	6487	2	5.00	6.00	1	.00
618	6487	2	6.00	6.00	0	.00
619	6487	2	7.00	6.00	0	.00
620	6487	2	8.00	6.00	0	.00
621	?	?	?	?	?	?
622	?	?	?	?	?	?
623	?	?	?	?	?	?
624	?	?	?	?	?	?
625	?	?	?	?	?	?
626	?	?	?	?	?	?
627	?	?	?	?	?	?
628	?	?	?	?	?	?
629	?	?	?	?	?	?
630	?	?	?	?	?	?

	Duration_chw	P_or_V_value_chw	Base_value_chw	Amplitude	onset_percentage_chw	duration_percentage_chw
596	.00	.00	.00	.00	.00	.00
597	.60	-12.79	17.17	4.38	-3.89	101.18
598	.59	39.17	-30.86	8.31	1.69	100.00
599	.00	.00	.00	.00	.00	.00
600	.00	.00	.00	.00	.00	.00
601	.00	.00	.00	.00	.00	.00
602	.59	36.69	-30.03	-6.66	1.18	100.00
603	.00	.00	.00	.00	.00	.00
604	.00	.00	.00	.00	.00	.00
605	.59	30.39	-26.55	3.84	.17	100.00
606	.00	.00	.00	.00	.00	.00
607	.00	.00	.00	.00	.00	.00
608	.00	.00	.00	.00	.00	.00
609	.60	-13.69	17.10	3.41	-2.53	101.18
610	.60	36.91	-30.64	6.27	1.18	101.18
611	.00	.00	.00	.00	.00	.00
612	.00	.00	.00	.00	.00	.00
613	.00	.00	.00	.00	.00	.00
614	.56	38.40	-30.25	-8.15	1.80	100.00
615	.00	.00	.00	.00	.00	.00
616	.00	.00	.00	.00	.00	.00
617	.56	30.60	-26.76	3.84	.18	100.00
618	.00	.00	.00	.00	.00	.00
619	.00	.00	.00	.00	.00	.00
620	.00	.00	.00	.00	.00	.00
621	.56	-13.01	17.07	4.06	?	?
622	.55	39.09	-30.90	8.19	?	?
623	.00	.00	.00	.00	?	?
624	.00	.00	.00	.00	?	?
625	.00	.00	.00	.00	?	?
626	.54	36.10	-29.13	-6.97	?	?
627	.00	.00	.00	.00	?	?
628	.00	.00	.00	.00	?	?
629	.53	30.23	-26.58	3.65	?	?
630	.00	.00	.00	.00	?	?

Sono Mastication_Raw data.sav

	amplitude_perc	chewing_side	Pig_code_sw	gender_sw	Pair_sw	Sw_episodes
596	.00	5.00	.	.	.	.
597	-34.25	2.00	.	.	.	.
598	-21.22	1.00	.	.	.	.
599	.00	2.00	.	.	.	.
600	.00	1.00	.	.	.	.
601	.00	4.00	.	.	.	.
602	-18.15	3.00	.	.	.	.
603	.00	4.00	.	.	.	.
604	.00	3.00	.	.	.	.
605	12.64	5.00	.	.	.	.
606	.00	5.00	.	.	.	.
607	.00	5.00	.	.	.	.
608	.00	5.00	.	.	.	.
609	-24.91	4.00	.	.	.	.
610	-16.99	3.00	.	.	.	.
611	.00	4.00	.	.	.	.
612	.00	3.00	.	.	.	.
613	.00	4.00	.	.	.	.
614	-21.22	3.00	.	.	.	.
615	.00	4.00	.	.	.	.
616	.00	3.00	.	.	.	.
617	12.55	5.00	.	.	.	.
618	.00	5.00	.	.	.	.
619	.00	5.00	.	.	.	.
620	.00	5.00	.	.	.	.
621	-31.21	4.00	.	.	.	.
622	-20.95	3.00	.	.	.	.
623	.00	4.00	.	.	.	.
624	.00	3.00	.	.	.	.
625	.00	2.00	.	.	.	.
626	-19.31	1.00	.	.	.	.
627	.00	2.00	.	.	.	.
628	.00	1.00	.	.	.	.
629	12.07	5.00	.	.	.	.
630	.00	5.00	.	.	.	.

Sono Mastication_Raw data.sav

	P_V_sw	Onset_sw	duration_sw	P_or_V_value_sw	Base_value_sw	Range
596	.	.	.	.	.	.
597	.	.	.	.	.	.
598	.	.	.	.	.	.
599	.	.	.	.	.	.
600	.	.	.	.	.	.
601	.	.	.	.	.	.
602	.	.	.	.	.	.
603	.	.	.	.	.	.
604	.	.	.	.	.	.
605	.	.	.	.	.	.
606	.	.	.	.	.	.
607	.	.	.	.	.	.
608	.	.	.	.	.	.
609	.	.	.	.	.	.
610	.	.	.	.	.	.
611	.	.	.	.	.	.
612	.	.	.	.	.	.
613	.	.	.	.	.	.
614	.	.	.	.	.	.
615	.	.	.	.	.	.
616	.	.	.	.	.	.
617	.	.	.	.	.	.
618	.	.	.	.	.	.
619	.	.	.	.	.	.
620	.	.	.	.	.	.
621	.	.	?	?	?	?
622	.	.	?	?	?	?
623	.	.	?	?	?	?
624	.	.	?	?	?	?
625	.	.	?	?	?	?
626	.	.	?	?	?	?
627	.	.	?	?	?	?
628	.	.	?	?	?	?
629	.	.	?	?	?	?
630	.	.	?	?	?	?

	onset_percent_sw	duration_percent_sw	Range_percent	Pause_before_sw	Pause_after_sw
596	.	.	.	.	.
597	.	.	.	.	.
598	.	.	.	.	.
599	.	.	.	.	.
600	.	.	.	.	.
601	.	.	.	.	.
602	.	.	.	.	.
603	.	.	.	.	.
604	.	.	.	.	.
605	.	.	.	.	.
606	.	.	.	.	.
607	.	.	.	.	.
608	.	.	.	.	.
609	.	.	.	.	.
610	.	.	.	.	.
611	.	.	.	.	.
612	.	.	.	.	.
613	.	.	.	.	.
614	.	.	.	.	.
615	.	.	.	.	.
616	.	.	.	.	.
617	.	.	.	.	.
618	.	.	.	.	.
619	.	.	.	.	.
620	.	.	.	.	.
621	.	.	.	.	.
622	.	.	.	.	.
623	.	.	.	.	.
624	.	.	.	.	.
625	.	.	.	.	.
626	.	.	.	.	.
627	.	.	.	.	.
628	.	.	.	.	.
629	.	.	.	.	.
630	.	.	.	.	.

	Pig_chw	Gender_chw	Pair_chw	Cycles	P_V_chw	Onset_chw
631	6487	2	7.00	7.00	0	.00
632	6487	2	8.00	7.00	0	.00
633	6487	2	9.00	7.00	2	-.03
634	6487	2	10.00	7.00	2	.01
635	6487	2	11.00	7.00	0	.00
636	6487	2	12.00	7.00	0	.00
637	6487	2	1.00	8.00	0	.00
638	6487	2	2.00	8.00	2	.01
639	6487	2	3.00	8.00	0	.00
640	6487	2	4.00	8.00	0	.00
641	6487	2	5.00	8.00	1	.00
642	6487	2	6.00	8.00	0	.00
643	6487	2	7.00	8.00	0	.00
644	6487	2	8.00	8.00	0	.00
645	6487	2	9.00	8.00	2	-.02
646	6487	2	10.00	8.00	2	.01
647	6487	2	11.00	8.00	0	.00
648	6487	2	12.00	8.00	0	.00
649	6487	2	1.00	9.00	0	.00
650	6487	2	2.00	9.00	2	-.01
651	6487	2	3.00	9.00	0	.00
652	6487	2	4.00	9.00	0	.00
653	6487	2	5.00	9.00	1	.00
654	6487	2	6.00	9.00	0	.00
655	6487	2	7.00	9.00	0	.00
656	6487	2	8.00	9.00	0	.00
657	6487	2	9.00	9.00	2	-.03
658	6487	2	10.00	9.00	2	.01
659	6487	2	11.00	9.00	0	.00
660	6487	2	12.00	9.00	0	.00
661	?	?	?	?	?	?
662	?	?	?	?	?	?
663	?	?	?	?	?	?
664	?	?	?	?	?	?
665	?	?	?	?	?	?

Sono Mastication_Raw data.sav

	Duration_chw	P_or_V_value_chw	Base_value_chw	Amplitude	onset_percentage_chw	duration_percentage_chw
631	.00	.00	.00	.00	.00	.00
632	.00	.00	.00	.00	.00	.00
633	.56	-13.69	17.59	3.90	-5.56	102.78
634	.54	36.45	-29.92	6.53	1.85	100.00
635	.00	.00	.00	.00	.00	.00
636	.00	.00	.00	.00	.00	.00
637	.00	.00	.00	.00	.00	.00
638	.57	39.17	-30.43	-8.74	1.41	100.00
639	.00	.00	.00	.00	.00	.00
640	.00	.00	.00	.00	.00	.00
641	.57	30.93	-27.40	3.53	.18	100.00
642	.00	.00	.00	.00	.00	.00
643	.00	.00	.00	.00	.00	.00
644	.00	.00	.00	.00	.00	.00
645	.57	-12.58	17.14	4.56	-2.64	100.00
646	.56	39.92	-31.36	8.56	1.41	97.54
647	.00	.00	.00	.00	.00	.00
648	.00	.00	.00	.00	.00	.00
649	.00	.00	.00	.00	.00	.00
650	.59	36.95	-28.98	-7.97	-1.35	100.00
651	.00	.00	.00	.00	.00	.00
652	.00	.00	.00	.00	.00	.00
653	.59	30.18	-26.64	3.54	.17	100.00
654	.00	.00	.00	.00	.00	.00
655	.00	.00	.00	.00	.00	.00
656	.00	.00	.00	.00	.00	.00
657	.60	-13.40	17.69	4.29	-5.07	101.18
658	.60	37.33	-29.82	7.51	1.35	101.18
659	.00	.00	.00	.00	.00	.00
660	.00	.00	.00	.00	.00	.00
661	.00	.00	.00	.00	?	?
662	.64	37.19	-28.76	-8.43	?	?
663	.00	.00	.00	.00	?	?
664	.00	.00	.00	.00	?	?
665	.63	29.87	-26.55	3.32	?	?

Sono Mastication_Raw data.sav

	amplitude_perc	chewing_side	Pig_code_sw	gender_sw	Pair_sw	Sw_episodes
631	.00	5.00	.	.	.	.
632	.00	5.00	.	.	.	.
633	-28.49	2.00	.	.	.	.
634	-17.92	1.00	.	.	.	.
635	.00	2.00	.	.	.	.
636	.00	1.00	.	.	.	.
637	.00	2.00	.	.	.	.
638	-22.31	1.00	.	.	.	.
639	.00	2.00	.	.	.	.
640	.00	1.00	.	.	.	.
641	11.41	5.00	.	.	.	.
642	.00	5.00	.	.	.	.
643	.00	5.00	.	.	.	.
644	.00	5.00	.	.	.	.
645	-36.25	2.00	.	.	.	.
646	-21.44	1.00	.	.	.	.
647	.00	2.00	.	.	.	.
648	.00	1.00	.	.	.	.
649	.00	2.00	.	.	.	.
650	-21.57	1.00	.	.	.	.
651	.00	2.00	.	.	.	.
652	.00	1.00	.	.	.	.
653	11.73	5.00	.	.	.	.
654	.00	5.00	.	.	.	.
655	.00	5.00	.	.	.	.
656	.00	5.00	.	.	.	.
657	-32.02	2.00	.	.	.	.
658	-20.12	1.00	.	.	.	.
659	.00	2.00	.	.	.	.
660	.00	1.00	.	.	.	.
661	.00	2.00	.	.	.	.
662	-22.67	1.00	.	.	.	.
663	.00	2.00	.	.	.	.
664	.00	1.00	.	.	.	.
665	11.12	5.00	.	.	.	.

Sono Mastication_Raw data.sav

	P_V_sw	Onset_sw	duration_sw	P_or_V_value_sw	Base_value_sw	Range
631	.	.	.	.	.	.
632	.	.	.	.	.	.
633	.	.	.	.	.	.
634	.	.	.	.	.	.
635	.	.	.	.	.	.
636	.	.	.	.	.	.
637	.	.	.	.	.	.
638	.	.	.	.	.	.
639	.	.	.	.	.	.
640	.	.	.	.	.	.
641	.	.	.	.	.	.
642	.	.	.	.	.	.
643	.	.	.	.	.	.
644	.	.	.	.	.	.
645	.	.	.	.	.	.
646	.	.	.	.	.	.
647	.	.	.	.	.	.
648	.	.	.	.	.	.
649	.	.	.	.	.	.
650	.	.	.	.	.	.
651	.	.	.	.	.	.
652	.	.	.	.	.	.
653	.	.	.	.	.	.
654	.	.	.	.	.	.
655	.	.	.	.	.	.
656	.	.	.	.	.	.
657	.	.	.	.	.	.
658	.	.	.	.	.	.
659	.	.	.	.	.	.
660	.	.	.	.	.	.
661	.	.	?	?	?	?
662	.	.	?	?	?	?
663	.	.	?	?	?	?
664	.	.	?	?	?	?
665	.	.	?	?	?	?

	onset_percent_sw	duration_percent_sw	Range_percent	Pause_before_sw	Pause_after_sw
631	.	.	.	.	.
632	.	.	.	.	.
633	.	.	.	.	.
634	.	.	.	.	.
635	.	.	.	.	.
636	.	.	.	.	.
637	.	.	.	.	.
638	.	.	.	.	.
639	.	.	.	.	.
640	.	.	.	.	.
641	.	.	.	.	.
642	.	.	.	.	.
643	.	.	.	.	.
644	.	.	.	.	.
645	.	.	.	.	.
646	.	.	.	.	.
647	.	.	.	.	.
648	.	.	.	.	.
649	.	.	.	.	.
650	.	.	.	.	.
651	.	.	.	.	.
652	.	.	.	.	.
653	.	.	.	.	.
654	.	.	.	.	.
655	.	.	.	.	.
656	.	.	.	.	.
657	.	.	.	.	.
658	.	.	.	.	.
659	.	.	.	.	.
660	.	.	.	.	.
661	.	.	.	.	.
662	.	.	.	.	.
663	.	.	.	.	.
664	.	.	.	.	.
665	.	.	.	.	.

Sono Mastication_Raw data.sav

	Pig_chw	Gender_chw	Pair_chw	Cycles	P_V_chw	Onset_chw
666	6487	2	6.00	10.00	0	.00
667	6487	2	7.00	10.00	0	.00
668	6487	2	8.00	10.00	0	.00
669	6487	2	9.00	10.00	2	-.02
670	6487	2	10.00	10.00	2	.01
671	6487	2	11.00	10.00	0	.00
672	6487	2	12.00	10.00	0	.00
673	6487	2	1.00	11.00	0	.00
674	6487	2	2.00	11.00	2	.02
675	6487	2	3.00	11.00	0	.00
676	6487	2	4.00	11.00	0	.00
677	6487	2	5.00	11.00	1	.00
678	6487	2	6.00	11.00	0	.00
679	6487	2	7.00	11.00	0	.00
680	6487	2	8.00	11.00	0	.00
681	6487	2	9.00	11.00	2	-.01
682	6487	2	10.00	11.00	2	.02
683	6487	2	11.00	11.00	0	.00
684	6487	2	12.00	11.00	0	.00
685	6487	2	1.00	14.00	0	.00
686	6487	2	2.00	14.00	2	.01
687	6487	2	3.00	14.00	0	.00
688	6487	2	4.00	14.00	0	.00
689	6487	2	5.00	14.00	1	.00
690	6487	2	6.00	14.00	0	.00
691	6487	2	7.00	14.00	0	.00
692	6487	2	8.00	14.00	0	.00
693	6487	2	9.00	14.00	2	-.03
694	6487	2	10.00	14.00	2	.01
695	6487	2	11.00	14.00	0	.00
696	6487	2	12.00	14.00	0	.00
697	6238	1	1.00	1.00	0	.00
698	6238	1	2.00	1.00	0	.00
699	6238	1	3.00	1.00	2	-.04
700	6238	1	4.00	1.00	1	.05

	Duration_chw	P_or_V_value_c hw	Base_value_c hw	Amplitude	onset_percen tage_chw	duration_perc entage_chw
666	.00	.00	.00	.00	.00	.00
667	.00	.00	.00	.00	.00	.00
668	.00	.00	.00	.00	.00	.00
669	.64	-12.86	17.59	4.73	-2.39	101.27
670	.64	37.88	-29.56	8.32	1.59	101.27
671	.00	.00	.00	.00	.00	.00
672	.00	.00	.00	.00	.00	.00
673	.00	.00	.00	.00	.00	.00
674	.55	38.89	-29.88	-9.01	2.74	100.00
675	.00	.00	.00	.00	.00	.00
676	.00	.00	.00	.00	.00	.00
677	.55	29.43	-26.99	2.44	.18	100.00
678	.00	.00	.00	.00	.00	.00
679	.00	.00	.00	.00	.00	.00
680	.00	.00	.00	.00	.00	.00
681	.54	-12.73	17.33	4.60	-1.46	98.72
682	.54	39.55	-30.78	8.77	2.74	98.72
683	.00	.00	.00	.00	.00	.00
684	.00	.00	.00	.00	.00	.00
685	.00	.00	.00	.00	.00	.00
686	.56	36.82	-28.62	-8.20	1.23	98.77
687	.00	.00	.00	.00	.00	.00
688	.00	.00	.00	.00	.00	.00
689	.57	30.05	-27.23	2.82	.18	100.00
690	.00	.00	.00	.00	.00	.00
691	.00	.00	.00	.00	.00	.00
692	.00	.00	.00	.00	.00	.00
693	.58	-12.85	17.80	4.95	-5.27	102.64
694	.57	37.62	-29.74	7.88	1.76	100.00
695	.00	.00	.00	.00	.00	.00
696	.00	.00	.00	.00	.00	.00
697	.00	.00	.00	.00	.00	.00
698	.00	.00	.00	.00	.00	.00
699	.61	-12.19	14.28	2.09	-6.25	103.72
700	.47	17.63	-16.46	1.17	8.78	78.55

Sono Mastication_Raw data.sav

	amplitude_perc	chewing_side	Pig_code_sw	gender_sw	Pair_sw	Sw_episodes
666	.00	5.00	.	.	.	.
667	.00	5.00	.	.	.	.
668	.00	5.00	.	.	.	.
669	-36.78	2.00	.	.	.	.
670	-21.96	1.00	.	.	.	.
671	.00	2.00	.	.	.	.
672	.00	1.00	.	.	.	.
673	.00	4.00	.	.	.	.
674	-23.17	3.00	.	.	.	.
675	.00	4.00	.	.	.	.
676	.00	3.00	.	.	.	.
677	8.29	5.00	.	.	.	.
678	.00	5.00	.	.	.	.
679	.00	5.00	.	.	.	.
680	.00	5.00	.	.	.	.
681	-36.14	4.00	.	.	.	.
682	-22.17	3.00	.	.	.	.
683	.00	4.00	.	.	.	.
684	.00	3.00	.	.	.	.
685	.00	2.00	.	.	.	.
686	-22.27	1.00	.	.	.	.
687	.00	2.00	.	.	.	.
688	.00	1.00	.	.	.	.
689	9.38	5.00	.	.	.	.
690	.00	5.00	.	.	.	.
691	.00	5.00	.	.	.	.
692	.00	5.00	.	.	.	.
693	-38.52	2.00	.	.	.	.
694	-20.95	1.00	.	.	.	.
695	.00	2.00	.	.	.	.
696	.00	1.00	.	.	.	.
697	.00	4.00	.	.	.	.
698	.00	3.00	.	.	.	.
699	-17.15	4.00	.	.	.	.
700	6.64	3.00	.	.	.	.

	P_V_sw	Onset_sw	duration_sw	P_or_V_value_sw	Base_value_sw	Range
666	.	.	.	.	.	.
667	.	.	.	.	.	.
668	.	.	.	.	.	.
669	.	.	.	.	.	.
670	.	.	.	.	.	.
671	.	.	.	.	.	.
672	.	.	.	.	.	.
673	.	.	.	.	.	.
674	.	.	.	.	.	.
675	.	.	.	.	.	.
676	.	.	.	.	.	.
677	.	.	.	.	.	.
678	.	.	.	.	.	.
679	.	.	.	.	.	.
680	.	.	.	.	.	.
681	.	.	.	.	.	.
682	.	.	.	.	.	.
683	.	.	.	.	.	.
684	.	.	.	.	.	.
685	.	.	.	.	.	.
686	.	.	.	.	.	.
687	.	.	.	.	.	.
688	.	.	.	.	.	.
689	.	.	.	.	.	.
690	.	.	.	.	.	.
691	.	.	.	.	.	.
692	.	.	.	.	.	.
693	.	.	.	.	.	.
694	.	.	.	.	.	.
695	.	.	.	.	.	.
696	.	.	.	.	.	.
697	.	.	.	.	.	.
698	.	.	.	.	.	.
699	.	.	.	.	.	.
700	.	.	.	.	.	.

	onset_percent_sw	duration_percent_sw	Range_percent	Pause_before_sw	Pause_after_sw
666	.	.	.	.	.
667	.	.	.	.	.
668	.	.	.	.	.
669	.	.	.	.	.
670	.	.	.	.	.
671	.	.	.	.	.
672	.	.	.	.	.
673	.	.	.	.	.
674	.	.	.	.	.
675	.	.	.	.	.
676	.	.	.	.	.
677	.	.	.	.	.
678	.	.	.	.	.
679	.	.	.	.	.
680	.	.	.	.	.
681	.	.	.	.	.
682	.	.	.	.	.
683	.	.	.	.	.
684	.	.	.	.	.
685	.	.	.	.	.
686	.	.	.	.	.
687	.	.	.	.	.
688	.	.	.	.	.
689	.	.	.	.	.
690	.	.	.	.	.
691	.	.	.	.	.
692	.	.	.	.	.
693	.	.	.	.	.
694	.	.	.	.	.
695	.	.	.	.	.
696	.	.	.	.	.
697	.	.	.	.	.
698	.	.	.	.	.
699	.	.	.	.	.
700	.	.	.	.	.

Sono Mastication_Raw data.sav

	Pig_chw	Gender_chw	Pair_chw	Cycles	P_V_chw	Onset_chw
701	6238	1	5.00	1.00	1	.00
702	6238	1	6.00	1.00	1	-.01
703	6238	1	7.00	1.00	0	.00
704	6238	1	8.00	1.00	0	.00
705	6238	1	9.00	1.00	0	.00
706	6238	1	10.00	1.00	0	.00
707	6238	1	11.00	1.00	0	.00
708	6238	1	12.00	1.00	2	-.10
709	6238	1	1.00	2.00	0	.00
710	6238	1	2.00	2.00	0	.00
711	6238	1	3.00	2.00	2	-.05
712	6238	1	4.00	2.00	1	.02
713	6238	1	5.00	2.00	1	.00
714	6238	1	6.00	2.00	1	-.03
715	6238	1	7.00	2.00	0	.00
716	6238	1	8.00	2.00	0	.00
717	6238	1	9.00	2.00	0	.00
718	6238	1	10.00	2.00	0	.00
719	6238	1	11.00	2.00	1	-.02
720	6238	1	12.00	2.00	2	-.02
721	6238	1	1.00	3.00	0	.00
722	6238	1	2.00	3.00	0	.00
723	6238	1	3.00	3.00	2	-.05
724	6238	1	4.00	3.00	1	-.09
725	6238	1	5.00	3.00	1	.00
726	6238	1	6.00	3.00	1	.02
727	6238	1	7.00	3.00	0	.00
728	6238	1	8.00	3.00	0	.00
729	6238	1	9.00	3.00	0	.00
730	6238	1	10.00	3.00	0	.00
731	6238	1	11.00	3.00	1	.11
732	6238	1	12.00	3.00	2	-.14
733	6238	1	1.00	4.00	0	.00
734	6238	1	2.00	4.00	0	.00
735	6238	1	3.00	4.00	2	-.06

Sono Mastication_Raw data.sav

	Duration_chw	P_or_V_value_chw	Base_value_chw	Amplitude	onset_percentage_chw	duration_percentage_chw
701	.59	23.86	-21.24	2.62	.17	100.00
702	.61	28.27	-24.73	3.54	-1.35	102.53
703	.00	.00	.00	.00	.00	.00
704	.00	.00	.00	.00	.00	.00
705	.00	.00	.00	.00	.00	.00
706	.00	.00	.00	.00	.00	.00
707	.00	.00	.00	.00	.00	.00
708	.63	-13.69	15.85	2.16	-16.39	106.25
709	.00	.00	.00	.00	.00	.00
710	.00	.00	.00	.00	.00	.00
711	.69	-12.83	13.54	.71	-8.27	109.54
712	.60	17.14	-16.25	.89	3.66	95.23
713	.67	24.80	-21.72	3.08	.16	106.04
714	.65	29.17	-26.15	3.02	-4.77	103.66
715	.00	.00	.00	.00	.00	.00
716	.00	.00	.00	.00	.00	.00
717	.00	.00	.00	.00	.00	.00
718	.00	.00	.00	.00	.00	.00
719	.68	4.77	-4.22	.55	-3.50	108.43
720	.46	-14.78	15.17	.39	-3.50	72.66
721	.00	.00	.00	.00	.00	.00
722	.00	.00	.00	.00	.00	.00
723	.53	-13.09	13.61	.52	-10.39	104.31
724	.53	18.22	-16.99	1.23	-17.65	102.94
725	.51	23.52	-21.91	1.61	.20	100.00
726	.52	28.63	-27.35	1.28	4.31	101.37
727	.00	.00	.00	.00	.00	.00
728	.00	.00	.00	.00	.00	.00
729	.00	.00	.00	.00	.00	.00
730	.00	.00	.00	.00	.00	.00
731	.42	4.76	-4.10	.66	20.59	82.35
732	.48	-15.20	15.76	.56	-28.04	94.12
733	.00	.00	.00	.00	.00	.00
734	.00	.00	.00	.00	.00	.00
735	.49	-12.74	13.60	.86	-9.77	79.32

Sono Mastication_Raw data.sav

	amplitude_perc	chewing_side	Pig_code_sw	gender_sw	Pair_sw	Sw_episodes
701	10.98	5.00	.	.	.	.
702	12.52	5.00	.	.	.	.
703	.00	5.00	.	.	.	.
704	.00	5.00	.	.	.	.
705	.00	4.00	.	.	.	.
706	.00	3.00	.	.	.	.
707	.00	4.00	.	.	.	.
708	-15.78	3.00	.	.	.	.
709	.00	4.00	.	.	.	.
710	.00	3.00	.	.	.	.
711	-5.53	4.00	.	.	.	.
712	5.19	3.00	.	.	.	.
713	12.42	5.00	.	.	.	.
714	10.35	5.00	.	.	.	.
715	.00	5.00	.	.	.	.
716	.00	5.00	.	.	.	.
717	.00	4.00	.	.	.	.
718	.00	3.00	.	.	.	.
719	11.53	4.00	.	.	.	.
720	-2.64	3.00	.	.	.	.
721	.00	2.00	.	.	.	.
722	.00	1.00	.	.	.	.
723	-3.97	2.00	.	.	.	.
724	6.75	1.00	.	.	.	.
725	6.85	5.00	.	.	.	.
726	4.47	5.00	.	.	.	.
727	.00	5.00	.	.	.	.
728	.00	5.00	.	.	.	.
729	.00	2.00	.	.	.	.
730	.00	1.00	.	.	.	.
731	13.87	2.00	.	.	.	.
732	-3.68	1.00	.	.	.	.
733	.00	2.00	.	.	.	.
734	.00	1.00	.	.	.	.
735	-6.75	2.00	.	.	.	.

	P_V_sw	Onset_sw	duration_sw	P_or_V_value_sw	Base_value_sw	Range
701	.	.	.	.	.	.
702	.	.	.	.	.	.
703	.	.	.	.	.	.
704	.	.	.	.	.	.
705	.	.	.	.	.	.
706	.	.	.	.	.	.
707	.	.	.	.	.	.
708	.	.	.	.	.	.
709	.	.	.	.	.	.
710	.	.	.	.	.	.
711	.	.	.	.	.	.
712	.	.	.	.	.	.
713	.	.	.	.	.	.
714	.	.	.	.	.	.
715	.	.	.	.	.	.
716	.	.	.	.	.	.
717	.	.	.	.	.	.
718	.	.	.	.	.	.
719	.	.	.	.	.	.
720	.	.	.	.	.	.
721	.	.	.	.	.	.
722	.	.	.	.	.	.
723	.	.	.	.	.	.
724	.	.	.	.	.	.
725	.	.	.	.	.	.
726	.	.	.	.	.	.
727	.	.	.	.	.	.
728	.	.	.	.	.	.
729	.	.	.	.	.	.
730	.	.	.	.	.	.
731	.	.	.	.	.	.
732	.	.	.	.	.	.
733	.	.	.	.	.	.
734	.	.	.	.	.	.
735	.	.	.	.	.	.

	onset_percent_sw	duration_percent_sw	Range_percent	Pause_before_sw	Pause_after_sw
701	.	.	.	.	.
702	.	.	.	.	.
703	.	.	.	.	.
704	.	.	.	.	.
705	.	.	.	.	.
706	.	.	.	.	.
707	.	.	.	.	.
708	.	.	.	.	.
709	.	.	.	.	.
710	.	.	.	.	.
711	.	.	.	.	.
712	.	.	.	.	.
713	.	.	.	.	.
714	.	.	.	.	.
715	.	.	.	.	.
716	.	.	.	.	.
717	.	.	.	.	.
718	.	.	.	.	.
719	.	.	.	.	.
720	.	.	.	.	.
721	.	.	.	.	.
722	.	.	.	.	.
723	.	.	.	.	.
724	.	.	.	.	.
725	.	.	.	.	.
726	.	.	.	.	.
727	.	.	.	.	.
728	.	.	.	.	.
729	.	.	.	.	.
730	.	.	.	.	.
731	.	.	.	.	.
732	.	.	.	.	.
733	.	.	.	.	.
734	.	.	.	.	.
735	.	.	.	.	.

Sono Mastication_Raw data.sav

	Pig_chw	Gender_chw	Pair_chw	Cycles	P_V_chw	Onset_chw
736	6238	1	4.00	4.00	1	.02
737	6238	1	5.00	4.00	1	.00
738	6238	1	6.00	4.00	1	.01
739	6238	1	7.00	4.00	0	.00
740	6238	1	8.00	4.00	0	.00
741	6238	1	9.00	4.00	0	.00
742	6238	1	10.00	4.00	0	.00
743	6238	1	11.00	4.00	1	-.03
744	6238	1	12.00	4.00	2	-.13
745	6238	1	1.00	5.00	0	.00
746	6238	1	2.00	5.00	0	.00
747	6238	1	3.00	5.00	2	-.05
748	6238	1	4.00	5.00	1	.02
749	6238	1	5.00	5.00	1	.00
750	6238	1	6.00	5.00	1	.03
751	6238	1	7.00	5.00	0	.00
752	6238	1	8.00	5.00	0	.00
753	6238	1	9.00	5.00	0	.00
754	6238	1	10.00	5.00	0	.00
755	6238	1	11.00	5.00	1	.01
756	6238	1	12.00	5.00	2	-.13
757	6238	1	1.00	6.00	0	.00
758	6238	1	2.00	6.00	0	.00
759	6238	1	3.00	6.00	2	-.03
760	6238	1	4.00	6.00	1	.02
761	?	?	?	?	?	?
762	?	?	?	?	?	?
763	?	?	?	?	?	?
764	?	?	?	?	?	?
765	?	?	?	?	?	?
766	?	?	?	?	?	?
767	?	?	?	?	?	?
768	?	?	?	?	?	?
769	?	?	?	?	?	?
770	?	?	?	?	?	?

	Duration_chw	P_or_V_value_chw	Base_value_chw	Amplitude	onset_percentage_chw	duration_percentage_chw
736	.34	16.41	-16.18	.23	2.44	54.89
737	.47	24.39	-21.86	2.53	.22	68.99
738	.45	28.90	-26.44	2.46	1.63	73.29
739	.00	.00	.00	.00	.00	.00
740	.00	.00	.00	.00	.00	.00
741	.00	.00	.00	.00	.00	.00
742	.00	.00	.00	.00	.00	.00
743	.51	4.99	-4.36	.63	-5.98	101.59
744	.51	-14.19	15.50	1.31	-25.50	101.59
745	.00	.00	.00	.00	.00	.00
746	.00	.00	.00	.00	.00	.00
747	.53	-12.50	13.83	1.33	-10.56	104.58
748	.39	16.48	-15.57	.91	4.38	77.69
749	.50	24.51	-22.03	2.48	.20	100.00
750	.47	29.16	-26.95	2.21	5.98	94.02
751	.00	.00	.00	.00	.00	.00
752	.00	.00	.00	.00	.00	.00
753	.00	.00	.00	.00	.00	.00
754	.00	.00	.00	.00	.00	.00
755	.51	5.09	-4.40	.69	1.39	101.59
756	.58	-13.82	15.19	1.37	-25.50	114.94
757	.00	.00	.00	.00	.00	.00
758	.00	.00	.00	.00	.00	.00
759	.61	-12.13	14.17	2.04	-4.94	101.15
760	.61	17.68	-15.50	2.18	2.47	100.00
761	.61	25.36	-22.14	3.22	?	?
762	.64	29.84	-27.03	2.81	?	?
763	.00	.00	.00	.00	?	?
764	.00	.00	.00	.00	?	?
765	.00	.00	.00	.00	?	?
766	.00	.00	.00	.00	?	?
767	.60	5.02	-4.24	.78	?	?
768	.61	-13.81	15.88	2.07	?	?
769	.00	.00	.00	.00	?	?
770	.00	.00	.00	.00	?	?

Sono Mastication_Raw data.sav

	amplitude_perc	chewing_side	Pig_code_sw	gender_sw	Pair_sw	Sw_episodes
736	1.40	1.00	.	.	.	.
737	10.37	5.00	.	.	.	.
738	8.51	5.00	.	.	.	.
739	.00	5.00	.	.	.	.
740	.00	5.00	.	.	.	.
741	.00	2.00	.	.	.	.
742	.00	1.00	.	.	.	.
743	12.63	2.00	.	.	.	.
744	-9.23	1.00	.	.	.	.
745	.00	2.00	.	.	.	.
746	.00	1.00	.	.	.	.
747	-10.64	2.00	.	.	.	.
748	5.52	1.00	.	.	.	.
749	10.12	5.00	.	.	.	.
750	7.58	5.00	.	.	.	.
751	.00	5.00	.	.	.	.
752	.00	5.00	.	.	.	.
753	.00	2.00	.	.	.	.
754	.00	1.00	.	.	.	.
755	13.56	2.00	.	.	.	.
756	-9.91	1.00	.	.	.	.
757	.00	2.00	.	.	.	.
758	.00	1.00	.	.	.	.
759	-16.82	2.00	.	.	.	.
760	12.33	1.00	.	.	.	.
761	12.70	5.00	.	.	.	.
762	9.42	5.00	.	.	.	.
763	.00	5.00	.	.	.	.
764	.00	5.00	.	.	.	.
765	.00	2.00	.	.	.	.
766	.00	1.00	.	.	.	.
767	15.54	2.00	.	.	.	.
768	-14.99	1.00	.	.	.	.
769	.00	4.00	.	.	.	.
770	.00	3.00	.	.	.	.

	P_V_sw	Onset_sw	duration_sw	P_or_V_value_sw	Base_value_sw	Range
736	.	.	.	.	.	.
737	.	.	.	.	.	.
738	.	.	.	.	.	.
739	.	.	.	.	.	.
740	.	.	.	.	.	.
741	.	.	.	.	.	.
742	.	.	.	.	.	.
743	.	.	.	.	.	.
744	.	.	.	.	.	.
745	.	.	.	.	.	.
746	.	.	.	.	.	.
747	.	.	.	.	.	.
748	.	.	.	.	.	.
749	.	.	.	.	.	.
750	.	.	.	.	.	.
751	.	.	.	.	.	.
752	.	.	.	.	.	.
753	.	.	.	.	.	.
754	.	.	.	.	.	.
755	.	.	.	.	.	.
756	.	.	.	.	.	.
757	.	.	.	.	.	.
758	.	.	.	.	.	.
759	.	.	.	.	.	.
760	.	.	.	.	.	.
761	.	.	?	?	?	?
762	.	.	?	?	?	?
763	.	.	?	?	?	?
764	.	.	?	?	?	?
765	.	.	?	?	?	?
766	.	.	?	?	?	?
767	.	.	?	?	?	?
768	.	.	?	?	?	?
769	.	.	?	?	?	?
770	.	.	?	?	?	?

	onset_percent_sw	duration_percent_sw	Range_percent	Pause_before_sw	Pause_after_sw
736	.	.	.	.	.
737	.	.	.	.	.
738	.	.	.	.	.
739	.	.	.	.	.
740	.	.	.	.	.
741	.	.	.	.	.
742	.	.	.	.	.
743	.	.	.	.	.
744	.	.	.	.	.
745	.	.	.	.	.
746	.	.	.	.	.
747	.	.	.	.	.
748	.	.	.	.	.
749	.	.	.	.	.
750	.	.	.	.	.
751	.	.	.	.	.
752	.	.	.	.	.
753	.	.	.	.	.
754	.	.	.	.	.
755	.	.	.	.	.
756	.	.	.	.	.
757	.	.	.	.	.
758	.	.	.	.	.
759	.	.	.	.	.
760	.	.	.	.	.
761	.	.	.	.	.
762	.	.	.	.	.
763	.	.	.	.	.
764	.	.	.	.	.
765	.	.	.	.	.
766	.	.	.	.	.
767	.	.	.	.	.
768	.	.	.	.	.
769	.	.	.	.	.
770	.	.	.	.	.

Sono Mastication_Raw data.sav

	Pig_chw	Gender_chw	Pair_chw	Cycles	P_V_chw	Onset_chw
771	6238	1	3.00	7.00	2	-.06
772	6238	1	4.00	7.00	1	-.02
773	6238	1	5.00	7.00	1	.00
774	6238	1	6.00	7.00	1	-.01
775	6238	1	7.00	7.00	0	.00
776	6238	1	8.00	7.00	0	.00
777	6238	1	9.00	7.00	0	.00
778	6238	1	10.00	7.00	0	.00
779	6238	1	11.00	7.00	1	-.05
780	6238	1	12.00	7.00	2	-.07
781	6238	1	1.00	8.00	0	.00
782	6238	1	2.00	8.00	0	.00
783	6238	1	3.00	8.00	2	-.14
784	6238	1	4.00	8.00	1	-.03
785	6238	1	5.00	8.00	1	.00
786	6238	1	6.00	8.00	1	-.01
787	6238	1	7.00	8.00	0	.00
788	6238	1	8.00	8.00	0	.00
789	6238	1	9.00	8.00	0	.00
790	6238	1	10.00	8.00	0	.00
791	6238	1	11.00	8.00	1	-.08
792	6238	1	12.00	8.00	2	.13
793	6238	1	1.00	9.00	0	.00
794	6238	1	2.00	9.00	0	.00
795	6238	1	3.00	9.00	2	.02
796	6238	1	4.00	9.00	1	-.08
797	6238	1	5.00	9.00	1	.00
798	6238	1	6.00	9.00	1	.01
799	6238	1	7.00	9.00	0	.00
800	6238	1	8.00	9.00	0	.00
801	?	?	?	?	?	?
802	?	?	?	?	?	?
803	?	?	?	?	?	?
804	?	?	?	?	?	?
805	?	?	?	?	?	?

Sono Mastication_Raw data.sav

	Duration_chw	P_or_V_value_chw	Base_value_chw	Amplitude	onset_percentage_chw	duration_percentage_chw
771	.65	-12.14	14.18	2.04	-8.25	89.68
772	.70	17.87	-16.30	1.57	-3.03	96.84
773	.73	25.79	-22.33	3.46	.14	100.00
774	.69	29.35	-27.11	2.24	-.96	94.77
775	.00	.00	.00	.00	.00	.00
776	.00	.00	.00	.00	.00	.00
777	.00	.00	.00	.00	.00	.00
778	.00	.00	.00	.00	.00	.00
779	.65	4.76	-4.18	.58	-7.15	89.68
780	.66	-13.58	15.82	2.24	-9.35	90.65
781	.00	.00	.00	.00	.00	.00
782	.00	.00	.00	.00	.00	.00
783	.70	-12.59	14.68	2.09	-20.76	101.16
784	.68	17.93	-16.53	1.40	-4.35	98.98
785	.69	25.65	-21.68	3.97	.15	100.00
786	.73	29.90	-26.36	3.54	-1.16	106.53
787	.00	.00	.00	.00	.00	.00
788	.00	.00	.00	.00	.00	.00
789	.00	.00	.00	.00	.00	.00
790	.00	.00	.00	.00	.00	.00
791	.83	4.82	-4.03	.79	-10.89	120.76
792	.38	-14.51	15.11	.60	19.45	54.43
793	.00	.00	.00	.00	.00	.00
794	.00	.00	.00	.00	.00	.00
795	.48	-12.62	13.24	.62	2.99	95.62
796	.48	17.50	-16.31	1.19	-16.34	95.62
797	.50	22.68	-20.17	2.51	.20	100.00
798	.41	27.97	-24.88	3.09	1.99	82.07
799	.00	.00	.00	.00	.00	.00
800	.00	.00	.00	.00	.00	.00
801	.00	.00	.00	.00	?	?
802	.00	.00	.00	.00	?	?
803	.47	4.74	-4.19	.55	?	?
804	.00	.00	.00	.00	?	?
805	.00	.00	.00	.00	?	?

Sono Mastication_Raw data.sav

	amplitude_perc	chewing_side	Pig_code_sw	gender_sw	Pair_sw	Sw_episodes
771	-16.80	4.00	.	.	.	.
772	8.79	3.00	.	.	.	.
773	13.42	5.00	.	.	.	.
774	7.63	5.00	.	.	.	.
775	.00	5.00	.	.	.	.
776	.00	5.00	.	.	.	.
777	.00	4.00	.	.	.	.
778	.00	3.00	.	.	.	.
779	12.19	4.00	.	.	.	.
780	-16.50	3.00	.	.	.	.
781	.00	4.00	.	.	.	.
782	.00	3.00	.	.	.	.
783	-16.60	4.00	.	.	.	.
784	7.81	3.00	.	.	.	.
785	15.48	5.00	.	.	.	.
786	11.84	5.00	.	.	.	.
787	.00	5.00	.	.	.	.
788	.00	5.00	.	.	.	.
789	.00	4.00	.	.	.	.
790	.00	3.00	.	.	.	.
791	16.39	4.00	.	.	.	.
792	-4.14	3.00	.	.	.	.
793	.00	2.00	.	.	.	.
794	.00	1.00	.	.	.	.
795	-4.91	2.00	.	.	.	.
796	6.80	1.00	.	.	.	.
797	11.07	5.00	.	.	.	.
798	11.05	5.00	.	.	.	.
799	.00	5.00	.	.	.	.
800	.00	5.00	.	.	.	.
801	.00	2.00	.	.	.	.
802	.00	1.00	.	.	.	.
803	11.60	2.00	.	.	.	.
804	.00	1.00	.	.	.	.
805	.00	2.00	.	.	.	.

	P_V_sw	Onset_sw	duration_sw	P_or_V_value_sw	Base_value_sw	Range
771	.	.	.	.	.	.
772	.	.	.	.	.	.
773	.	.	.	.	.	.
774	.	.	.	.	.	.
775	.	.	.	.	.	.
776	.	.	.	.	.	.
777	.	.	.	.	.	.
778	.	.	.	.	.	.
779	.	.	.	.	.	.
780	.	.	.	.	.	.
781	.	.	.	.	.	.
782	.	.	.	.	.	.
783	.	.	.	.	.	.
784	.	.	.	.	.	.
785	.	.	.	.	.	.
786	.	.	.	.	.	.
787	.	.	.	.	.	.
788	.	.	.	.	.	.
789	.	.	.	.	.	.
790	.	.	.	.	.	.
791	.	.	.	.	.	.
792	.	.	.	.	.	.
793	.	.	.	.	.	.
794	.	.	.	.	.	.
795	.	.	.	.	.	.
796	.	.	.	.	.	.
797	.	.	.	.	.	.
798	.	.	.	.	.	.
799	.	.	.	.	.	.
800	.	.	.	.	.	.
801	.	.	?	?	?	?
802	.	.	?	?	?	?
803	.	.	?	?	?	?
804	.	.	?	?	?	?
805	.	.	?	?	?	?

	onset_percent_sw	duration_percent_sw	Range_percent	Pause_before_sw	Pause_after_sw
771	.	.	.	.	.
772	.	.	.	.	.
773	.	.	.	.	.
774	.	.	.	.	.
775	.	.	.	.	.
776	.	.	.	.	.
777	.	.	.	.	.
778	.	.	.	.	.
779	.	.	.	.	.
780	.	.	.	.	.
781	.	.	.	.	.
782	.	.	.	.	.
783	.	.	.	.	.
784	.	.	.	.	.
785	.	.	.	.	.
786	.	.	.	.	.
787	.	.	.	.	.
788	.	.	.	.	.
789	.	.	.	.	.
790	.	.	.	.	.
791	.	.	.	.	.
792	.	.	.	.	.
793	.	.	.	.	.
794	.	.	.	.	.
795	.	.	.	.	.
796	.	.	.	.	.
797	.	.	.	.	.
798	.	.	.	.	.
799	.	.	.	.	.
800	.	.	.	.	.
801	.	.	.	.	.
802	.	.	.	.	.
803	.	.	.	.	.
804	.	.	.	.	.
805	.	.	.	.	.

Sono Mastication_Raw data.sav

	Pig_chw	Gender_chw	Pair_chw	Cycles	P_V_chw	Onset_chw
806	6238	1	2.00	10.00	0	.00
807	6238	1	3.00	10.00	2	.05
808	6238	1	4.00	10.00	1	-.02
809	6238	1	5.00	10.00	1	.00
810	6238	1	6.00	10.00	1	.03
811	6238	1	7.00	10.00	0	.00
812	6238	1	8.00	10.00	0	.00
813	6238	1	9.00	10.00	0	.00
814	6238	1	10.00	10.00	0	.00
815	6238	1	11.00	10.00	1	-.04
816	6238	1	12.00	10.00	2	-.08
817	6238	1	1.00	11.00	0	.00
818	6238	1	2.00	11.00	0	.00
819	6238	1	3.00	11.00	2	-.04
820	6238	1	4.00	11.00	1	.04
821	6238	1	5.00	11.00	1	.00
822	6238	1	6.00	11.00	1	.01
823	6238	1	7.00	11.00	0	.00
824	6238	1	8.00	11.00	0	.00
825	6238	1	9.00	11.00	0	.00
826	6238	1	10.00	11.00	0	.00
827	6238	1	11.00	11.00	1	.01
828	6238	1	12.00	11.00	2	.00
829	6238	1	1.00	12.00	0	.00
830	6238	1	2.00	12.00	0	.00
831	6238	1	3.00	12.00	2	-.05
832	6238	1	4.00	12.00	1	.01
833	6238	1	5.00	12.00	1	.00
834	6238	1	6.00	12.00	1	-.01
835	6238	1	7.00	12.00	0	.00
836	6238	1	8.00	12.00	0	.00
837	6238	1	9.00	12.00	0	.00
838	6238	1	10.00	12.00	0	.00
839	6238	1	11.00	12.00	1	-.04
840	6238	1	12.00	12.00	2	-.08

	Duration_chw	P_or_V_value_chw	Base_value_chw	Amplitude	onset_percentage_chw	duration_percentage_chw
806	.00	.00	.00	.00	.00	.00
807	.46	-12.36	13.15	.79	8.57	87.05
808	.48	17.00	-16.64	.36	-4.19	91.43
809	.53	22.91	-20.48	2.43	.19	100.00
810	.50	27.62	-25.64	1.98	5.71	94.29
811	.00	.00	.00	.00	.00	.00
812	.00	.00	.00	.00	.00	.00
813	.00	.00	.00	.00	.00	.00
814	.00	.00	.00	.00	.00	.00
815	.58	5.02	-4.54	.48	-7.05	109.91
816	.56	-12.98	13.90	.92	-15.81	107.05
817	.00	.00	.00	.00	.00	.00
818	.00	.00	.00	.00	.00	.00
819	.70	-13.01	14.40	1.39	-5.52	101.16
820	.68	17.19	-16.21	.98	5.52	98.98
821	.69	23.37	-21.12	2.25	.15	100.00
822	.68	28.12	-25.93	2.19	1.45	98.98
823	.00	.00	.00	.00	.00	.00
824	.00	.00	.00	.00	.00	.00
825	.00	.00	.00	.00	.00	.00
826	.00	.00	.00	.00	.00	.00
827	.67	5.03	-4.43	.60	1.31	96.81
828	.60	-13.02	14.38	1.36	-.15	86.94
829	.00	.00	.00	.00	.00	.00
830	.00	.00	.00	.00	.00	.00
831	.61	-13.15	14.51	1.36	-7.51	101.34
832	.51	17.11	-16.40	.71	1.34	85.14
833	.60	23.23	-20.94	2.29	.17	100.00
834	.60	28.05	-26.16	1.89	-1.17	100.00
835	.00	.00	.00	.00	.00	.00
836	.00	.00	.00	.00	.00	.00
837	.00	.00	.00	.00	.00	.00
838	.00	.00	.00	.00	.00	.00
839	.63	4.82	-4.49	.33	-6.18	105.01
840	.65	-13.15	14.66	1.51	-13.69	108.85

Sono Mastication_Raw data.sav

	amplitude_perc	chewing_side	Pig_code_sw	gender_sw	Pair_sw	Sw_episodes
806	.00	1.00	.	.	.	.
807	-6.39	2.00	.	.	.	.
808	2.12	1.00	.	.	.	.
809	10.61	5.00	.	.	.	.
810	7.17	5.00	.	.	.	.
811	.00	5.00	.	.	.	.
812	.00	5.00	.	.	.	.
813	.00	2.00	.	.	.	.
814	.00	1.00	.	.	.	.
815	9.56	2.00	.	.	.	.
816	-7.09	1.00	.	.	.	.
817	.00	2.00	.	.	.	.
818	.00	1.00	.	.	.	.
819	-10.68	2.00	.	.	.	.
820	5.70	1.00	.	.	.	.
821	9.63	5.00	.	.	.	.
822	7.79	5.00	.	.	.	.
823	.00	5.00	.	.	.	.
824	.00	5.00	.	.	.	.
825	.00	2.00	.	.	.	.
826	.00	1.00	.	.	.	.
827	11.93	2.00	.	.	.	.
828	-10.45	1.00	.	.	.	.
829	.00	2.00	.	.	.	.
830	.00	1.00	.	.	.	.
831	-10.34	2.00	.	.	.	.
832	4.15	1.00	.	.	.	.
833	9.86	5.00	.	.	.	.
834	6.74	5.00	.	.	.	.
835	.00	5.00	.	.	.	.
836	.00	5.00	.	.	.	.
837	.00	2.00	.	.	.	.
838	.00	1.00	.	.	.	.
839	6.85	2.00	.	.	.	.
840	-11.48	1.00	.	.	.	.

	P_V_sw	Onset_sw	duration_sw	P_or_V_value_sw	Base_value_sw	Range
806	.	.	.	.	.	.
807	.	.	.	.	.	.
808	.	.	.	.	.	.
809	.	.	.	.	.	.
810	.	.	.	.	.	.
811	.	.	.	.	.	.
812	.	.	.	.	.	.
813	.	.	.	.	.	.
814	.	.	.	.	.	.
815	.	.	.	.	.	.
816	.	.	.	.	.	.
817	.	.	.	.	.	.
818	.	.	.	.	.	.
819	.	.	.	.	.	.
820	.	.	.	.	.	.
821	.	.	.	.	.	.
822	.	.	.	.	.	.
823	.	.	.	.	.	.
824	.	.	.	.	.	.
825	.	.	.	.	.	.
826	.	.	.	.	.	.
827	.	.	.	.	.	.
828	.	.	.	.	.	.
829	.	.	.	.	.	.
830	.	.	.	.	.	.
831	.	.	.	.	.	.
832	.	.	.	.	.	.
833	.	.	.	.	.	.
834	.	.	.	.	.	.
835	.	.	.	.	.	.
836	.	.	.	.	.	.
837	.	.	.	.	.	.
838	.	.	.	.	.	.
839	.	.	.	.	.	.
840	.	.	.	.	.	.

	onset_percent_sw	duration_percent_sw	Range_percent	Pause_before_sw	Pause_after_sw
806	.	.	.	.	.
807	.	.	.	.	.
808	.	.	.	.	.
809	.	.	.	.	.
810	.	.	.	.	.
811	.	.	.	.	.
812	.	.	.	.	.
813	.	.	.	.	.
814	.	.	.	.	.
815	.	.	.	.	.
816	.	.	.	.	.
817	.	.	.	.	.
818	.	.	.	.	.
819	.	.	.	.	.
820	.	.	.	.	.
821	.	.	.	.	.
822	.	.	.	.	.
823	.	.	.	.	.
824	.	.	.	.	.
825	.	.	.	.	.
826	.	.	.	.	.
827	.	.	.	.	.
828	.	.	.	.	.
829	.	.	.	.	.
830	.	.	.	.	.
831	.	.	.	.	.
832	.	.	.	.	.
833	.	.	.	.	.
834	.	.	.	.	.
835	.	.	.	.	.
836	.	.	.	.	.
837	.	.	.	.	.
838	.	.	.	.	.
839	.	.	.	.	.
840	.	.	.	.	.

Sono Mastication_Raw data.sav

	Pig_chw	Gender_chw	Pair_chw	Cycles	P_V_chw	Onset_chw
841	6238	1	1.00	13.00	0	.00
842	6238	1	2.00	13.00	2	-.16
843	6238	1	3.00	13.00	2	-.08
844	6238	1	4.00	13.00	1	.01
845	6238	1	5.00	13.00	1	.00
846	6238	1	6.00	13.00	1	.06
847	6238	1	7.00	13.00	0	.00
848	6238	1	8.00	13.00	0	.00
849	6238	1	9.00	13.00	0	.00
850	6238	1	10.00	13.00	0	.00
851	6238	1	11.00	13.00	1	-.08
852	6238	1	12.00	13.00	2	-.09
853	6238	1	1.00	14.00	0	.00
854	6238	1	2.00	14.00	2	.05
855	6238	1	3.00	14.00	2	-.05
856	6238	1	4.00	14.00	1	-.09
857	6238	1	5.00	14.00	1	.00
858	6238	1	6.00	14.00	1	.02
859	6238	1	7.00	14.00	0	.00
860	6238	1	8.00	14.00	0	.00
861	6238	1	9.00	14.00	0	.00
862	6238	1	10.00	14.00	0	.00
863	6238	1	11.00	14.00	1	-.05
864	6238	1	12.00	14.00	2	-.11

Sono Mastication_Raw data.sav

	Duration_chw	P_or_V_value_chw	Base_value_chw	Amplitude	onset_percentage_chw	duration_percentage_chw
841	.00	.00	.00	.00	.00	.00
842	.59	-11.99	12.48	-.49	-25.24	95.18
843	.64	-12.56	13.15	.59	-13.18	103.54
844	.55	15.90	-15.58	.32	1.61	87.94
845	.62	23.16	-21.11	2.05	.16	100.00
846	.49	26.97	-25.60	1.37	9.65	78.30
847	.00	.00	.00	.00	.00	.00
848	.00	.00	.00	.00	.00	.00
849	.00	.00	.00	.00	.00	.00
850	.00	.00	.00	.00	.00	.00
851	.61	5.07	-4.78	.29	-12.06	98.71
852	.61	-12.71	13.08	.37	-14.47	98.71
853	.00	.00	.00	.00	.00	.00
854	.27	-12.16	12.23	-.07	9.53	57.20
855	.47	-12.73	13.18	.45	-11.23	98.52
856	.35	15.93	-15.64	.29	-19.07	74.58
857	.47	22.67	-20.87	1.80	.21	100.00
858	.47	26.68	-25.74	.94	3.18	98.52
859	.00	.00	.00	.00	.00	.00
860	.00	.00	.00	.00	.00	.00
861	.00	.00	.00	.00	.00	.00
862	.00	.00	.00	.00	.00	.00
863	.47	4.93	-4.78	.15	-9.53	100.00
864	.47	-12.82	13.16	.34	-23.94	98.52

Sono Mastication_Raw data.sav

	amplitude_perc	chewing_side	Pig_code_sw	gender_sw	Pair_sw	Sw_episodes
841	.00	4.00	.	.	.	.
842	-4.09	3.00	.	.	.	.
843	-4.70	4.00	.	.	.	.
844	2.01	3.00	.	.	.	.
845	11.12	5.00	.	.	.	.
846	5.08	5.00	.	.	.	.
847	.00	5.00	.	.	.	.
848	.00	5.00	.	.	.	.
849	.00	4.00	.	.	.	.
850	.00	3.00	.	.	.	.
851	5.72	4.00	.	.	.	.
852	-2.91	3.00	.	.	.	.
853	.00	4.00	.	.	.	.
854	-.58	3.00	.	.	.	.
855	-3.54	4.00	.	.	.	.
856	1.82	3.00	.	.	.	.
857	7.94	5.00	.	.	.	.
858	3.52	5.00	.	.	.	.
859	.00	5.00	.	.	.	.
860	.00	5.00	.	.	.	.
861	.00	4.00	.	.	.	.
862	.00	3.00	.	.	.	.
863	90.47	4.00	.	.	.	.
864	-74.58	3.00	.	.	.	.

Sono Mastication_Raw data.sav

	P_V_sw	Onset_sw	duration_sw	P_or_V_value_sw	Base_value_sw	Range
841	.	.	.	.	.	.
842	.	.	.	.	.	.
843	.	.	.	.	.	.
844	.	.	.	.	.	.
845	.	.	.	.	.	.
846	.	.	.	.	.	.
847	.	.	.	.	.	.
848	.	.	.	.	.	.
849	.	.	.	.	.	.
850	.	.	.	.	.	.
851	.	.	.	.	.	.
852	.	.	.	.	.	.
853	.	.	.	.	.	.
854	.	.	.	.	.	.
855	.	.	.	.	.	.
856	.	.	.	.	.	.
857	.	.	.	.	.	.
858	.	.	.	.	.	.
859	.	.	.	.	.	.
860	.	.	.	.	.	.
861	.	.	.	.	.	.
862	.	.	.	.	.	.
863	.	.	.	.	.	.
864	.	.	.	.	.	.

Sono Mastication_Raw data.sav

	onset_percent_sw	duration_percent_sw	Range_percent	Pause_before_sw	Pause_after_sw
841	.	.	.	.	.
842	.	.	.	.	.
843	.	.	.	.	.
844	.	.	.	.	.
845	.	.	.	.	.
846	.	.	.	.	.
847	.	.	.	.	.
848	.	.	.	.	.
849	.	.	.	.	.
850	.	.	.	.	.
851	.	.	.	.	.
852	.	.	.	.	.
853	.	.	.	.	.
854	.	.	.	.	.
855	.	.	.	.	.
856	.	.	.	.	.
857	.	.	.	.	.
858	.	.	.	.	.
859	.	.	.	.	.
860	.	.	.	.	.
861	.	.	.	.	.
862	.	.	.	.	.
863	.	.	.	.	.
864	.	.	.	.	.
