# Supplementary material for: The dynamic shape changes of the tongue base during respiration, chewing and swallowing
Source: PLoS One. 2025 Apr 14;20(4):e0315885. doi: 10.1371/journal.pone.0315885 (PMC11996074; doi:10.1371/journal.pone.0315885)
Supplement: Attachment — Submitted filename: Responses to the comments from Editors and Reviewers.docx [file pone.0315885.s005.docx]

**Responses to the comments from Editors and Reviewers**

Journal Requirements:
Please review your reference list to ensure that it is complete and correct. If you have cited papers that have been retracted, please include the rationale for doing so in the manuscript text, or remove these references and replace them with relevant current references. Any changes to the reference list should be mentioned in the rebuttal letter that accompanies your revised manuscript. If you need to cite a retracted article, indicate the article’s retracted status in the References list and also include a citation and full reference for the retraction notice.

**The reference list was reviewed, and it is complete and correct.**

1. If the authors have adequately addressed your comments raised in a previous round of review and you feel that this manuscript is now acceptable for publication, you may indicate that here to bypass the “Comments to the Author” section, enter your conflict of interest statement in the “Confidential to Editor” section, and submit your "Accept" recommendation.

Reviewer #1: (No Response)

Reviewer #2: All comments have been addressed

**Thank you for your positive evaluation.**

2. Is the manuscript technically sound, and do the data support the conclusions?

The manuscript must describe a technically sound piece of scientific research with data that supports the conclusions. Experiments must have been conducted rigorously, with appropriate controls, replication, and sample sizes. The conclusions must be drawn appropriately based on the data presented.

Reviewer #1: Yes

Reviewer #2: Yes

**Thank you for your positive evaluation.**

3. Has the statistical analysis been performed appropriately and rigorously?

Reviewer #1: Yes

Reviewer #2: Yes

**Thank you for your positive evaluation.**

4. Have the authors made all data underlying the findings in their manuscript fully available?

The [PLOS Data policy](https://urldefense.com/v3/__http:/track.editorialmanager.com/CL0/http:*2F*2Fwww.plosone.org*2Fstatic*2Fpolicies.action*23sharing/1/010f019511ceb422-ad8c07e3-52bc-4e5c-b806-5d42e548a849-000000/m3LUDNQXfSj9SnlclAZXSfJrVTF7qYbuID29jTKleXI=198__;JSUlJSU!!K-Hz7m0Vt54!kQkApqBXMmsSDDdJKiLqMStZtKJs8uQBrqvbgl0rLd-EMBkfvuPAB4ddDBHqHy6O7vtvLDa6187o-Q$) requires authors to make all data underlying the findings described in their manuscript fully available without restriction, with rare exception (please refer to the Data Availability Statement in the manuscript PDF file). The data should be provided as part of the manuscript or its supporting information, or deposited to a public repository. For example, in addition to summary statistics, the data points behind means, medians and variance measures should be available. If there are restrictions on publicly sharing data—e.g. participant privacy or use of data from a third party—those must be specified.

Reviewer #1: Yes

Reviewer #2: Yes

**Thank you for your positive evaluation.**

5. Is the manuscript presented in an intelligible fashion and written in standard English?

PLOS ONE does not copyedit accepted manuscripts, so the language in submitted articles must be clear, correct, and unambiguous. Any typographical or grammatical errors should be corrected at revision, so please note any specific errors here.

Reviewer #1: Yes

Reviewer #2: Yes

**Thank you for your positive evaluation.**

6. Review Comments to the Author

Please use the space provided to explain your answers to the questions above. You may also include additional comments for the author, including concerns about dual publication, research ethics, or publication ethics. (Please upload your review as an attachment if it exceeds 20,000 characters)

Reviewer #1: The authors responded appropriately to all of my comments. The manuscript has been greatly improved and is now in good condition. I would like to suggest a very minor correction.
1. Fig 2C EMG Scale: Please insert a space between the 500 µV value and the unit.

**A space was made between 500 and µV.**

2. P7 line19 “Respiration was recorded for 2 minutes under sedated sleep (Fig 2A). Then,the minipig was allowed to wake up for unrestrained feeding (Fig 2B) with regular pelletmixed with barium sulfate suspension (Vet-Paque, Jorgensen Laboratories Inc. USA).”
I think (Fig 2A) is a mistake for (Fig 2B), and (Fig 2B) is a mistake for (Fig 2C).

**Really appreciate the reviewer #1 to indicate these errors. These Figure citations were corrected in page 7.**
