# Supplementary material for: The dynamic shape changes of the tongue base during respiration, chewing and swallowing
Source: PLoS One. 2025 Apr 14;20(4):e0315885. doi: 10.1371/journal.pone.0315885 (PMC11996074; doi:10.1371/journal.pone.0315885)
Supplement: Attachment — Submitted filename: Response to Reviewers.docx [file pone.0315885.s004.docx]

**Responses to Journal Requirements and Reviewers’ Comments**

***Journal Requirements***

**1.** When submitting your revision, we need you to address these additional requirements. Please ensure that your manuscript meets PLOS ONE's style requirements, including those for file naming. The PLOS ONE style templates can be found at 
[https://journals.plos.org/plosone/s/file?id=wjVg/PLOSOne_formatting_sample_main_body.pdf](https://urldefense.com/v3/__https:/journals.plos.org/plosone/s/file?id=wjVg*PLOSOne_formatting_sample_main_body.pdf__;Lw!!K-Hz7m0Vt54!l5clGlCgq09E330o7K0YhH0iUH9nxem81k_pyt2m4qUMOqy-ZAHegAoVqrJJvzlenN2vtPPdy6XV8w$) and 
[https://journals.plos.org/plosone/s/file?id=ba62/PLOSOne_formatting_sample_title_authors_affiliations.pdf](https://urldefense.com/v3/__https:/journals.plos.org/plosone/s/file?id=ba62*PLOSOne_formatting_sample_title_authors_affiliations.pdf__;Lw!!K-Hz7m0Vt54!l5clGlCgq09E330o7K0YhH0iUH9nxem81k_pyt2m4qUMOqy-ZAHegAoVqrJJvzlenN2vtPNNWbU2xg$)

***Response:*** The protocol for Sonocrystal implantation is based on a previous publication from our group. We added the reference accordingly on page 6. The main body and title page were revised following the formatting instructions and templates.

**2.** Thank you for stating the following financial disclosure: 
NIH/NIDCR R01DE028864 to ZJL.

Please state what role the funders took in the study.  If the funders had no role, please state: ""The funders had no role in study design, data collection and analysis, decision to publish, or preparation of the manuscript."" 
If this statement is not correct you must amend it as needed. 
Please include this amended Role of Funder statement in your cover letter; we will change the online submission form on your behalf.

***Response:*** We provided accurate funding information. The sentence of Funding statement was amended to state that the funders had no role in study design, data collection and analysis, decision to publish, or preparation of the manuscript, and this statement is included in the cover letter.

**3.** Thank you for stating the following in the Acknowledgments Section of your manuscript: 
The authors would like to thank Sydney Honnlee, Sophia Devore, and Elliot Willis of the
University of Washington for their help with animal experiments and data collection. This
study was supported by grant R01DE028864 from NIH/NIDCR (Z.J.L).

We note that you have provided funding information that is not currently declared in your Funding Statement. However, funding information should not appear in the Acknowledgments section or other areas of your manuscript. We will only publish funding information present in the Funding Statement section of the online submission form. 
Please remove any funding-related text from the manuscript and let us know how you would like to update your Funding Statement. Currently, your Funding Statement reads as follows: 
NIH/NIDCR R01DE028864 to ZJL.

Please include your amended statements within your cover letter; we will change the online submission form on your behalf.

***Response:*** Funding information was removed from Acknowledgements and the Role of Funder Statement was added to the cover letter.

**4.** In the online submission form, you indicated that the datasets generated and/or analyzed during the current study are available from the corresponding author upon reasonable request.
 All PLOS journals now require all data underlying the findings described in their manuscript to be freely available to other researchers, either 1. In a public repository, 2. Within the manuscript itself, or 3. Uploaded as supplementary information.
This policy applies to all data except where public deposition would breach compliance with the protocol approved by your research ethics board. If your data cannot be made publicly available for ethical or legal reasons (e.g., public availability would compromise patient privacy), please explain your reasons for resubmission and your exemption request will be escalated for approval.

***Response:*** Thank you for your clarification. We made available the raw data on respiration, chewing and swallowing as supplementary information.

**5.** Please include your tables as part of your main manuscript and remove the individual files. Please note that supplementary tables (should remain/ be uploaded) as separate ""supporting information"" files.

***Response:*** Table 1 is now the part of the main body of the manuscript on page 10.

Additional Editor Comments :

Your paper has been reviewed. The comments of the reviewers are included at the bottom of this letter. The reviewers have recommended major revisions to your manuscript. Therefore, I invite you to revise and resubmit your manuscript as fast as possible.
Please carefully address the issues raised in the comments.

***Response:*** We appreciate all comments from the two thorough reviewers. Please find our responses as follows:

***Reviewer#1***

**1.** Is the manuscript technically sound, and do the data support the conclusions?
The manuscript must describe a technically sound piece of scientific research with data that supports the conclusions. Experiments must have been conducted rigorously, with appropriate controls, replication, and sample sizes. The conclusions must be drawn appropriately based on the data presented.

Reviewer #1: Partly

***Response****:* We appreciate the feedback. We added multiple editions throughout the manuscript to improve the technical soundness and scientific merits.

**2.** Has the statistical analysis been performed appropriately and rigorously?

Reviewer #1: Yes

***Response****:* We appreciate this positive feedback.

**3.** Have the authors made all data underlying the findings in their manuscript fully available?

The [PLOS Data policy](https://urldefense.com/v3/__http:/track.editorialmanager.com/CL0/http:*2F*2Fwww.plosone.org*2Fstatic*2Fpolicies.action*23sharing/1/010f019466399708-003a502b-8f98-4a77-867e-9c78e6c3b5c8-000000/DwTcToQJbssluGzg1UG-3RtysxmIddES5ZTfoMfEdTE=193__;JSUlJSU!!K-Hz7m0Vt54!l5clGlCgq09E330o7K0YhH0iUH9nxem81k_pyt2m4qUMOqy-ZAHegAoVqrJJvzlenN2vtPPbJ7lXmg$) requires authors to make all data underlying the findings described in their manuscript fully available without restriction, with rare exception (please refer to the Data Availability Statement in the manuscript PDF file). The data should be provided as part of the manuscript or its supporting information or deposited to a public repository. For example, in addition to summary statistics, the data points behind means, medians and variance measures should be available. If there are restrictions on publicly sharing data—e.g. participant privacy or use of data from a third party—those must be specified.

Reviewer #1: No

***Response:*** We appreciate the feedback. The raw data files obtained from the respiration, chewing and swallowing recordings are available as supporting information (S1 and S2 files).

**4.** Is the manuscript presented in an intelligible fashion and written in standard English?

PLOS ONE does not copyedit accepted manuscripts, so the language in submitted articles must be clear, correct, and unambiguous. Any typographical or grammatical errors should be corrected at revision, so please note any specific errors here.

Reviewer #1: Yes

***Response:*** We appreciate this positive feedback.

**5.** **Comments to the Author**

Please use the space provided to explain your answers to the questions above. You may also include additional comments for the author, including concerns about dual publication, research ethics, or publication ethics. (Please upload your review as an attachment if it exceeds 20,000 characters).

In this study, authors examined the deformational changes of the tongue base during respiration, mastication and swallowing in three dimensions by embedding ultrasonic piezoelectric (SONO) crystals in the tongue base of Yucatan minipigs. It is an interesting research topic, and the data could be important for understanding the mechanisms of respiration and swallowing disorders. However, it is difficult to judge the validity of the data because the quality of the figures overall is low and the explanations in the manuscript are insufficient. To improve these points, I think it is necessary to make significant revisions to the manuscript.

***Response:*** Many thanks for these comments. We revised all figures for accuracy and quality.

**Important points**

**1.** It is unclear how the starting timing of the peaks and valleys was determined in the waveform of the lengths, thicknesses and widths at the tongue base. In particular, the peak and valley occur repeatedly in succession during chewing. In such cases, it is important to clearly indicate where the starting timing is.

***Response:*** Thank you for the comment. Fig. 2A was added to show a sample of raw Sonometric recording which indicates the reference crystal pair (ADW, underlined) and the detection of onsets, peaks, valleys, and durations in all other crystal pairs. These standards were applied for all Sonometric signals on respiration, chewing, and swallowing.

**2.** The resolution of the EMG waveform is very low, making it difficult to clearly identify muscle activity. In particular, in the recording of the chewing episode shown in Fig 2B, it is difficult to find muscle activity that coincides with the rhythm of chewing, i.e. the timing of the opening and closing of the jaw. Is this typical muscle activity of the stylohyoid and masseter muscles while chewing in minipigs? According to the description in Materials and methods, it seems that this study is analyzing the deformation of the tongue base in the jaw opening phase and the jaw closing phase separately, so it is necessary to indicate some kind of indicator that shows the timing of the jaw opening phase and the jaw closing phase. Based on the following description in the manuscript, ‘The beginning of the cycle was detected at the jaw opening phase when the synchronized burst activity from EMG recordings, i.e., activation of the anterior digastric muscle, indicated elongation of the reference pair (ADW). (P12 line 7)’, I propose to show the EMG of the anterior digastric muscle at high resolution.

***Response:*** We highly appreciate these comments. We improved the EMG signals in Figure 2C. We added the activities of the digastric muscle for the detection of jaw opening, the masseter for jaw closing and power stroke of chewing, and the middle pharyngeal constrictor for the swallowing episode. In addition, we improve the quality of the videofluoroscopy (Figure 2D) to show the sonocrystals and the bolus with more clarity.

**3.** The authors recorded respiration under sedated sleep. I'm concerned that the sedation may affect the movement of the tongue base. Since chewing and swallowing can be recorded while awake, is there a reason why only respiration was recorded under sedated sleep? Please show the validity of recording under sedated sleep.

***Response:*** We are grateful for this comment. The minipig is active on awaking when not on feeding, thus it would be very difficult to measure the pure respiratory deformations of the tongue base. Therefore, the minipig was placed under sedation for the respiratory recording. Several literatures have indicated that the confounding effect of sedation and anesthesia on respiration has been proven to be minor (Oliven A, Odeh M, Geitini L, Oliven R, Steinfeld U, Schwartz AR, et al. Effect of co-activation of tongue protrusor and retractor muscles on pharyngeal lumen and airflow in sleep apnea patients. J Appl Physiol 2007; Oliven A, O'Hearn DJ, Boudewyns A, Odeh M, De Backer W, van de Heyning P, et al. Upper airway response to electrical stimulation of the genioglossus in obstructive sleep apnea. J Appl Physiol 2003;95(5):2023-9; Mak KH, Wang YT, Cheong TH, Poh SC. The effect of oral midazolam and diazepam on respiration in normal subjects. Eur Respir J 1993;6(1):42-7). On the other hand, we did record the respiratory features during chewing and swallowing, which was published elsewhere (Rosero-Salazar D., Honnlee S., and Liu Z.J.*: Tongue, palatal, hyoid and pharyngeal muscle activity during chewing, swallowing, and respiration. *Arch. Oral Biol*. 157: e105845, 2024).

**4.** The manuscript contains the following sentence: ‘Sample sizes for animals, analyzed cycles, and available SONO crystal pairs are summarized in Table 1.’ However, Table 1 is not included in the manuscript.

***Response:*** We appreciate this comment. Table 1 is now included in the manuscript on page 10.

**5.** Fig 4 indicates that, during chewing, the RPT shows the peak wave, while the LPT shows the valley wave. Does this mean that during the jaw opening phase of chewing, regardless of whether it is on the ipsilateral or contralateral side, the thickness of the right posterior region increases, while the thickness of the left posterior region decreases? This also applies to the relationship between RVL and LVL. If the data in Figs 3-5, which are not mentioned in the manuscript, are the combined data of eight animals, then it is thought that there is a certain tendency in the deformational changes in the left and right sides of the tongue base during chewing in minipigs. Describe and discuss the morphological changes on the left and right sides during chewing.

***Response:*** We appreciate such careful reading of our data. Fig. 4B do show that the ventral left and right lengths (RVL and LVL) and right and left posterior thicknesses had opposite deformational changes regardless of chewing side. These may indicate a left tipping of the tongue base during chewing, thus result in left elongation and posterior right thickening. These explanation was added to the results (p14) and discussion (p17).

**Minor points**

**1.** P6 line7
Is it correct to use a mouth mask to record breathing and a nasal catheter to record chewing? I wonder if the order is the other way round.

***Response:*** We appreciate this comment. The mouth mask ensures light sedation using isoflurane 2-3% mixed with oxygen when the data was recorded during respiration. However, chewing and swallowing data was recorded when animal was awake and underwent unrestraint feeding. Clearly, mouth mask could not be used during feeding. Therefore, mouth mask was replaced by a nasal catheter to monitor respiratory airflow during chewing and swallowing. A short explanation about this was added on page 5.

**2.** P11 line15 … valleys or shortening were observed in the posterior widths.
Fig. 2 seems to show that both PVW and PDW have peaks in the inspiratory phase, rather than valleys. Is this sentence in the manuscript correct?

***Response:*** We appreciate this comment. We improved all figures in terms of accuracy, clarity, and quality. The sentence in the manuscript is correct.


**3.** P12 line13 … the durations of deformational changes were …
In relation to the starting timing, please specify how authors determined the duration.

***Response:*** Thank you very much for this comment. Figs. 2B and 2C show in boxes the total cycle lengths for respiration (2B) and chewing (2C), The middle-dotted lines indicate the beginning of expiratory (2B) and jaw closing (2C) phases. The time scale is in second.

**4.** Fig 1
Add a scale to the top left color image.

***Response:*** Thank you very much for this comment. The image was enlarged to show the scale with a total of 10 mm. The note was added to the legend of this figure.

**5.** Fig 2
It is not clear which waveform the black arrow in the EMG in Figure B is pointing to. Please make this clear. In addition, indicate where the jaw opening phase is.
To clarify what is shown in the video fluoroscopy image in Figure C, indicate the names of the main structures in the image.
It is difficult to identify where the implanted SONO crystals are located within the white circle. Please indicate this more clearly.

***Response:*** Thank you for the feedback. Fig. 2 was improved for all aspects mentioned. Each part of this and other figures were built separately for the better quality.

**6.** Figs 3-5
The position of the box-and-whisker plots and the axis label are misaligned. In addition, the lines showing significant differences are also misaligned, so it is unclear which data is significant. Please arrange them.

***Response:*** Thank you for the feedback. The arrangements and editions were added accordingly.

**Reviewer# 2**

**1.** Is the manuscript technically sound, and do the data support the conclusions?

The manuscript must describe a technically sound piece of scientific research with data that supports the conclusions. Experiments must have been conducted rigorously, with appropriate controls, replication, and sample sizes. The conclusions must be drawn appropriately based on the data presented.

Reviewer #2: Yes

***Response:*** We appreciate the positive feedback.

**2.** Has the statistical analysis been performed appropriately and rigorously?

Reviewer #2: Yes

***Response:*** We appreciate the positive feedback.

**3.** Have the authors made all data underlying the findings in their manuscript fully available?

The [PLOS Data policy](https://urldefense.com/v3/__http:/track.editorialmanager.com/CL0/http:*2F*2Fwww.plosone.org*2Fstatic*2Fpolicies.action*23sharing/1/010f019466399708-003a502b-8f98-4a77-867e-9c78e6c3b5c8-000000/DwTcToQJbssluGzg1UG-3RtysxmIddES5ZTfoMfEdTE=193__;JSUlJSU!!K-Hz7m0Vt54!l5clGlCgq09E330o7K0YhH0iUH9nxem81k_pyt2m4qUMOqy-ZAHegAoVqrJJvzlenN2vtPPbJ7lXmg$) requires authors to make all data underlying the findings described in their manuscript fully available without restriction, with rare exception (please refer to the Data Availability Statement in the manuscript PDF file). The data should be provided as part of the manuscript or its supporting information or deposited to a public repository. For example, in addition to summary statistics, the data points behind means, medians and variance measures should be available. If there are restrictions on publicly sharing data—e.g. participant privacy or use of data from a third party—those must be specified.

Reviewer #2: Yes

***Response:*** Thank you for the positive feedback.

**4.** Is the manuscript presented in an intelligible fashion and written in standard English?

PLOS ONE does not copyedit accepted manuscripts, so the language in submitted articles must be clear, correct, and unambiguous. Any typographical or grammatical errors should be corrected at revision, so please note any specific errors here.

Reviewer #2: Yes

***Response:*** Thank you for the positive feedback.

**5. Comments to the Author**

Please use the space provided to explain your answers to the questions above. You may also include additional comments for the author, including concerns about dual publication, research ethics, or publication ethics. (Please upload your review as an attachment if it exceeds 20,000 characters)

Liu and colleagues investigated the movement of the tongue base by applying ultrasonic piezoelectric (SONO) crystals. The synchronization of EMG and videofluoroscopy provided new insights into the tongue base movement during breathing, chewing and swallowing. The strength of this manuscript primarily is that it conducted movement analysis from new perspective (method) as a functional study of chewing and swallowing. It was very interesting, and I would like to comment from a procedural and clinical perspective.

***Response:*** We highly appreciate these nice comments.

**Minor comments**

**1.** Please provide more details on the location of the SONO crystal implant. We would like more information, such as the definition of the base of the tongue.
***Response:*** We appreciate the comments. A few lines were added in the introduction and methods sections to complement this aspect. The Fig. 1 legend also provides a thorough description on the implantation.

**2.** Please indicate whether the animal was trained or fasted before recording.

***Response:*** Thank you for this comment. Page 4 indicates the frequency of the training, and the fasting period before recording. A few lines were added for clarification.

**3.** We believe that it would be better if the definition of swallowing was based not only on SG but also on the activity of the infrahyoid muscles and swallowing apnea.

***Response:*** Thank you for this valuable comment. The swallowing episode occurred simultaneously with activity burst of the middle pharyngeal constrictor (MC). The x-ray video fluoroscopy also showed this activity when the bolus was passing through the pharynx to the esophagus (Fig. 2D). We skipped the swallowing apnea in this manuscript as it is well described in our previous publication (Rosero-Salazar D., Honnlee S., and Liu Z.J.*: Tongue, palatal, hyoid and pharyngeal muscle activity during chewing, swallowing, and respiration. *Arch. Oral Biol*. 157: e105845, 2024).

**4.** Please write about the test food for chewing and swallowing recordings. I think that tongue movement changes depending on the amount of food eaten in one bite. What are your thoughts about this?

***Response:*** Thank you for this comment. Since the animal was fed unrestrainedly, the amount of food was not able to be controlled. However, the amount of ingested and chewed food should have been kept constant as a routine masticatory function. This feature was confirmed with similar bolus size for swallowing viewed from synchronized x-ray video fluoroscopy. We added this explanation on page 6.

**5.** Was the decision on the chewing side based only on EMG and videofluoroscopy? Didn't you record both masseter muscles?

***Response:*** Thank you for this comment. We only inserted EMG electrodes in the left side of the selected muscles including masseter and digastric muscles. The chewing side was determined by EMG activity of these two jaw muscles as we published previously (Kayalioglu M, Shcherbatyy V, Seifi A, Liu ZJ. Roles of intrinsic and extrinsic tongue muscles in feeding: electromyographic study in pigs. Arch Oral Biol. 2007;52(8):786-96.) A line was added on page 5 to explain this aspect.

**6.** Are there any differences in breathing, chewing, and swallowing behaviors between different species? Please describe any chewing or swallowing behaviors unique to mini pigs.

***Response:*** We appreciate this comment. The uniqueness of the minipig model we used is the similarity human in terms of masticatory apparatus and function, thus this model is suitable for later studies in swallowing and breathing disorders. Given the biological variations, we believe both breathing and masticatory behaviors are different across different species.

**7.** Did you investigate the changes in the shape of the tongue base in response to the flow of the food bolus in the oral cavity?

***Response:*** We did investigate the shape changes of the tongue base in response to the bolus movements into esophagus.

**8.** It is thought that the shape of the tongue base may change depending on the posture of the mini pig during recording, such as when it is facing down or forward. What are your thoughts on this?

***Response:*** We appreciate this comment. The minipig was fed unrestrainedly which means it was freely moving on the feeding table. To the best of our knowledge, the movements of the head or body may not relate to the shape changes of the tongue base during feeding.

**9.** Did you investigate the changes in the shape of the tongue base during liquid drinking?

***Response:*** Although our group did examine the shape changes of tongue body during water drinking using the same minipig model (Shcherbatyy V, Liu ZJ. Internal kinematics of the tongue during feeding in pigs. Anat Rec (Hoboken). 2007;290(10):1288-99), the same study was not performed in this study as unrestrained feeding with water or liquid is very difficult and the water restriction before the procedures are not allowed for the sake of animal welfare.
